# Non-invasive tumor microenvironment evaluation and treatment response prediction in gastric cancer using deep learning radiomics

**DOI:** 10.1016/j.xcrm.2023.101146

**Published:** 2023-08-08

**Authors:** Yuming Jiang, Kangneng Zhou, Zepang Sun, Hongyu Wang, Jingjing Xie, Taojun Zhang, Shengtian Sang, Md Tauhidul Islam, Jen-Yeu Wang, Chuanli Chen, Qingyu Yuan, Sujuan Xi, Tuanjie Li, Yikai Xu, Wenjun Xiong, Wei Wang, Guoxin Li, Ruijiang Li

**Affiliations:** 1 Department of General Surgery & Guangdong Provincial Key Laboratory of Precision Medicine for Gastrointestinal Tumor, Nanfang Hospital, Southern Medical University, Guangzhou, China; 2 Department of Radiation Oncology, Stanford University School of Medicine, Stanford, CA, USA; 3 School of Computer and Communication Engineering, University of Science and Technology Beijing, Beijing 100083, China; 4 Graduate Group of Epidemiology, University of California Davis, Davis, CA, USA; 5 Department of Medical Imaging Center, Nanfang Hospital, Southern Medical University, Guangzhou, China; 6 The Reproductive Medical Center, The Seventh Affiliated Hospital of Sun Yat-sen University, Shenzhen, China; 7 Department of Gastrointestinal Surgery, Guangdong Provincial Hospital of Chinese Medicine, The Second Affiliated Hospital of Guangzhou University of Chinese Medicine, Guangzhou, China; 8 Department of Gastric Surgery, and State Key Laboratory of Oncology in South China, Collaborative Innovation Center for Cancer Medicine, Sun Yat-sen University Cancer Center, Guangzhou, China

**Keywords:** tumor microenvironment, immunotherapy, deep learning, CT image, gastric cancer, radiomics, treatment response

## Abstract

The tumor microenvironment (TME) plays a critical role in disease progression and is a key determinant of therapeutic response in cancer patients. Here, we propose a noninvasive approach to predict the TME status from radiological images by combining radiomics and deep learning analyses. Using multi-institution cohorts of 2,686 patients with gastric cancer, we show that the radiological model accurately predicted the TME status and is an independent prognostic factor beyond clinicopathologic variables. The model further predicts the benefit from adjuvant chemotherapy for patients with localized disease. In patients treated with checkpoint blockade immunotherapy, the model predicts clinical response and further improves predictive accuracy when combined with existing biomarkers. Our approach enables noninvasive assessment of the TME, which opens the door for longitudinal monitoring and tracking response to cancer therapy. Given the routine use of radiologic imaging in oncology, our approach can be extended to many other solid tumor types.

## Highlights

• Deep learning radiomics enable noninvasive evaluation of tumor microenvironment
• Imaging signature predicts prognosis and chemotherapy benefit in gastric cancer
• Imaging signature predicts clinical response to immune checkpoint inhibitors

## Graphical abstract

**Figure:**
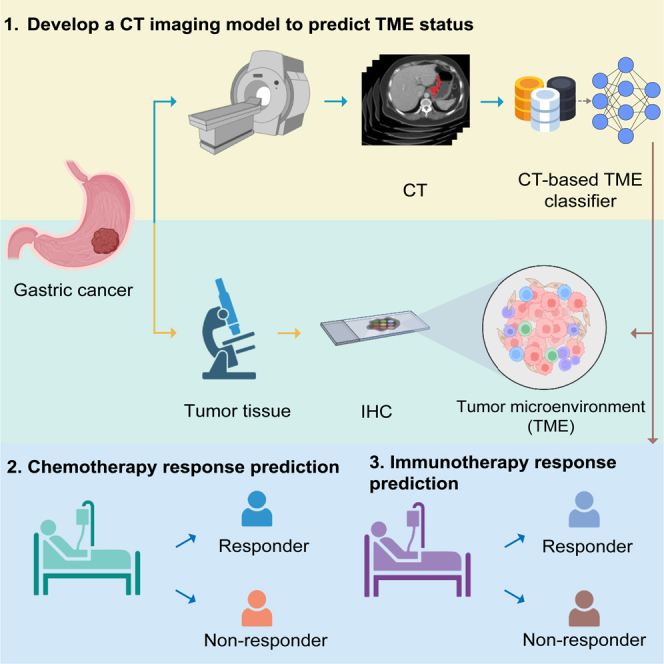

## Teaser

Jiang et al. develop an imaging signature by combined deep learning and radiomic analyses that allows noninvasive evaluation of the tumor microenvironment in gastric cancer. The imaging signature predicts the benefit from adjuvant chemotherapy as well as from clinical response to immunotherapy, demonstrating its potential for personalizing treatment of gastric cancer.

## Introduction

Gastric cancer (GC) is a highly prevalent malignancy and is the leading cause of cancer-related deaths worldwide.^1^ Currently, the most important factor in risk stratification and treatment decisions is the TNM staging system.^2^ However, large variations in treatment response and outcomes are observed for patients with disease of identical stage, suggesting that the current prognostic model could not provide complete predictive information.^2^^,^^3^ Therefore, an improved stratification of GC is needed to more accurately predict patient prognosis and treatment response.

Extensive research on the tumor microenvironment (TME) has shed new light on the molecularly based classification of cancers.^3^^,^^4^^,^^5^^,^^6^^,^^7^^,^^8^ Based on the quantification of various cell subpopulations in the TME, several biomarkers have been shown to be associated with prognosis and with response to chemotherapy and immunotherapy such as immune checkpoint inhibitors.^3^^,^^7^ In GC, patients whose tumors are infiltrated by cytotoxic CD8^+^ T lymphocytes have a prolonged survival, while those with a high amount of neutrophils have a poor prognosis.^3^^,^^7^^,^^9^

The gold standard for TME evaluation is based on histopathology. However, this approach suffers from the fundamental limitation of sampling bias due to intratumor heterogeneity^10^ and is also limited by insufficient tumor tissue available in practice. Therefore, a noninvasive means to assess the TME would be valuable, especially in the neoadjuvant therapy setting or metastatic disease.

Radiological imaging allows visualization of the entire tumor and is routinely used for diagnosis, staging, evaluation for treatment response, and follow up of patients with cancer. Sophisticated imaging analysis can reveal the link between subtle radiological phenotypes and specific aspects of the underlying pathobiology including the TME.^11^ Two broad approaches have been explored: (1) radiomics analysis with hand-crafted image features^12^^,^^13^^,^^14^ and (2) deep learning, which can automatically learn feature representations from images.^15^^,^^16^^,^^17^^,^^18^^,^^19^^,^^20^ While most applications are focused on clinical diagnosis, emerging studies have shown the feasibility of using deep learning to predict biological features from medical images.^21^

We previously developed and validated a machine learning classifier of the overall TME status based on immunohistochemistry assessment of eight immune and stroma features, which predicted survival and benefit from adjuvant chemotherapy in GC.^7^ In this study, we aim to develop a noninvasive imaging-based model of the TME classifier by combining deep learning and radiomics analysis. We will further validate the model for predicting prognosis and response to chemotherapy and immunotherapy.

## Results

### Patient characteristics

Table S1 lists the clinicopathological characteristics of the patients in the training (n = 398), internal validation 1 (n = 196), internal validation 2 (n = 602), external validation 1 (n = 101), and external validation 2 (n = 1,068) cohorts. All these patients (n = 2,365) had resection for GC (Figure S1). Among them, 1,615 (68.3%) were men, and the median age was 57 (interquartile range: 48.5–64) years. The majority of patients (n = 1,773, 75%) had stage II or III disease, among whom 881 (49.7%) patients received adjuvant chemotherapy. The clinicopathological data of the immunotherapy cohort are shown in Table S2.

### Deep learning radiomics model predicts TME classifier from CT images

**Figure 1:**
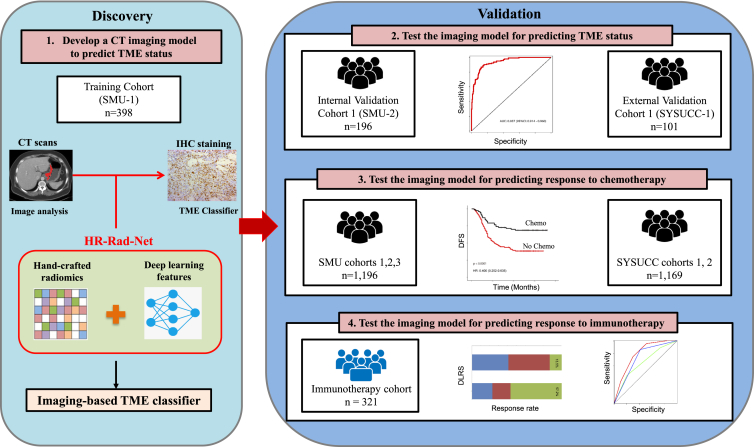
Study design for the discovery and validation of a deep learning model based on CT images to assess tumor microenvironment and treatment outcomes in gastric cancer Both CT images and IHC stains were available for patients in the SMU-1 (training) cohort and the SMU-2 and SYSUCC-1 (internal and external validation) cohorts, which were used for testing the model’s accuracy for predicting tumor microenvironment status. All patients had CT and treatment outcomes available, which were used for testing the prognostic and predictive value of the model. CT, computed tomography; IHC, immunohistochemistry; SMU, Southern Medical University; SYSUCC, Sun Yat-sen University Cancer Center.

**Figure 2:**
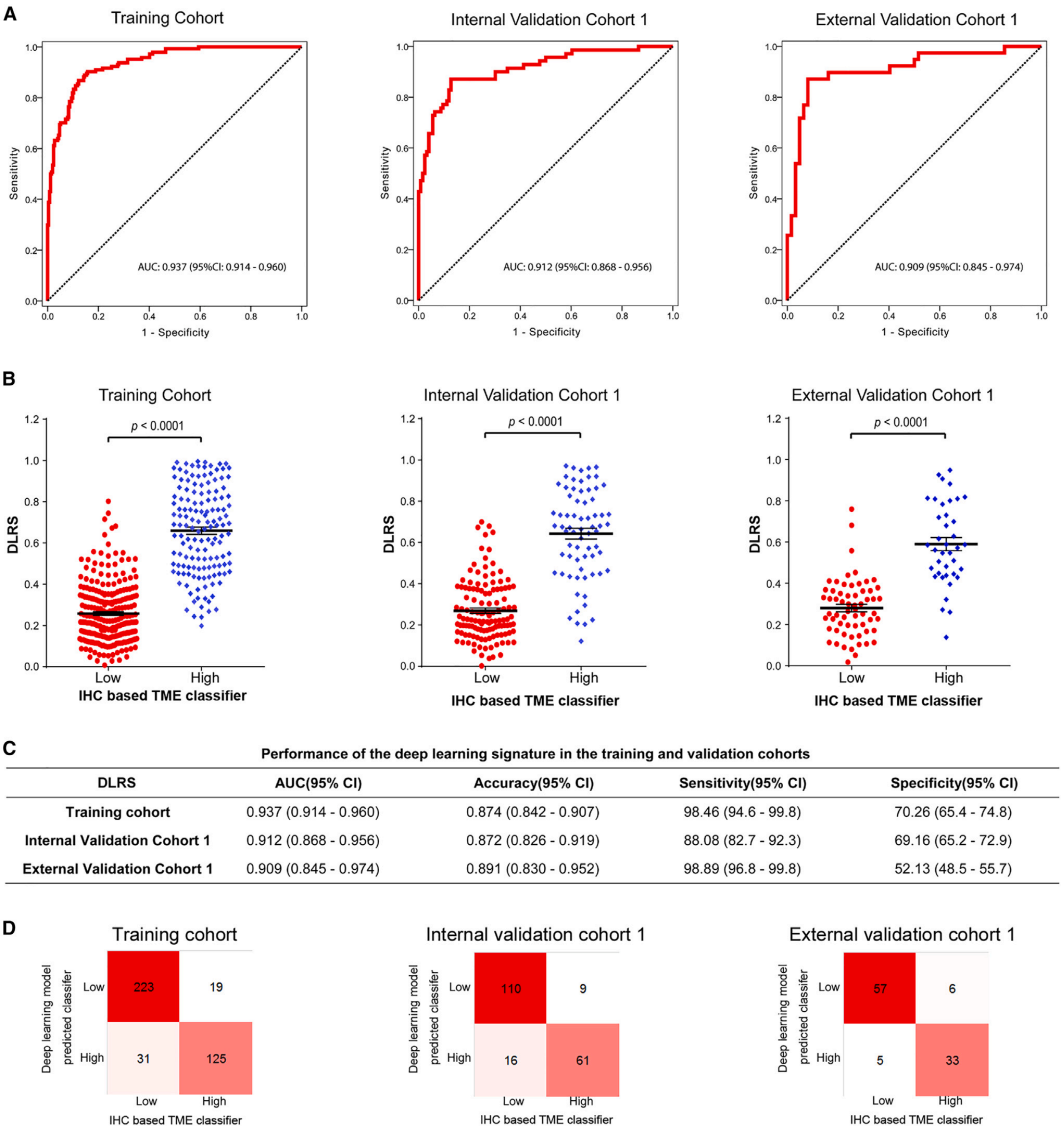
Performance of the deep learning model to assess tumor microenvironment in the training cohort, internal validation cohort 1, and external validation cohort 1 (A) Receiver operator characteristic (ROC) curves. (B) Distributions of DLRS by IHC-defined TME classifier. (C) Performance of the image signature in the training and validation cohorts. (D) Confusion matrices in the training and validation cohorts. The confusion matrices show the pairwise comparison; diagonal: number cases of correctly classified; off-diagonal: number cases of in correctly classified. AUC, area under the curves; TME, tumor microenvironment; DLRS: deep learning radiomics score.

We trained a deep learning radiomics model to predict the TME classifier from computed tomography (CT) images (Figures 1 and S2). The model combines a deep convolutional neural network with hand-crafted features to derive an imaging-based TME classifier, i.e., deep learning radiomics signature (DLRS). The implementation of the deep learning radiomics model is available at https://github.com/MontaEllis/HR-Rad-Net. Figures S3 shows some representative cases with CT images and visualization of the network prediction. The proposed model had an area under the curve (AUC) of 0.937 (95% confidence interval [CI], 0.914–0.960) for predicting the TME classifier in the training cohort (Figure 2A). Similarly, the model achieved high levels of discriminability with AUCs of 0.912 and 0.909 in the internal and external validation cohorts, respectively (Figure 2A). As expected, the model’s output DLRS was significantly higher in the TME-high group than that in the TME-low group in all three cohorts (Figure 2B). Additional performance metrics including the overall accuracy, sensitivity, and specificity are shown in Figure 2C. Consistently, the confusion matrix showed that the model predictions agreed well with the TME classifier defined by immunohistochemistry (IHC) (Figure 2D). The calibration curve also showed excellent agreement between the predicted probabilities for the TME classifier and observations in all cohorts (Figure S4A). The decision curve analysis showed a high net benefit for the model across a range of relevant threshold probabilities (Figure S4B).

Based on the optimum cutoff determined by the receiver operating characteristic (ROC) curve analysis in the training cohort (Figure 2A), patients were divided into a DLRS-low group (DLRSs < 0.428) and a DLRS-high group (DLRSs ≥ 0.428). The relationships between the DLRS and clinicopathological characteristics are summarized in Table S3.

Additionally, we compared performance of the proposed HR-Rad-Net model with alternative deep-learning approaches for predicting the TME classifier. In both validation cohorts, our model improved the prediction accuracy compared with the model trained without including radiomics features as well as the original HR-Net model trained without incorporating the squeeze and excitation strategy (Figure S5).

### DLRS is correlated with immune cell infiltration and stroma abundance

We assessed the relationship between DLRS and individual TME features in 695 patients for whom IHC data were available (i.e., by merging patients in the training cohort, internal validation cohort 1, and external validation cohort 1). This analysis revealed two distinct clusters: a dominant cluster of 9 features for DLRSs and lymphocytes (CD3TC, CD3IM, DLRS, CD8TC, CD8IM, CD45ROTC, CD45ROIM, CD57TC, and CD57IM) and a second cluster of 6 features for fibroblast, microvessel, and myeloid cells (a-SMA, CD34, CD66bTC, CD66bIM, CD68TC, and CD68IM) (Figure S6A). A positive correlation was observed between the DLRS and lymphocyte features (all p < 0.05), and a negative correlation was observed between the DLRS and features of fibroblast, vessel, and myeloid cells (Figures S6–S12). We also assessed the distribution of DLRSs, recurrence and survival status, and the expression of the 14 TME features. Tumors with high DLRSs generally exhibited increased expression of CD3IM, CD3TC, CD8IM, and CD45ROTC and reduced expression of fibroblast, microvessel, and myeloid cells. High-DLRS patients had fewer recurrences and deaths than low-DLRS patients did (Figures S13–S15).

### DLRS is associated with prognosis

**Figure 3:**
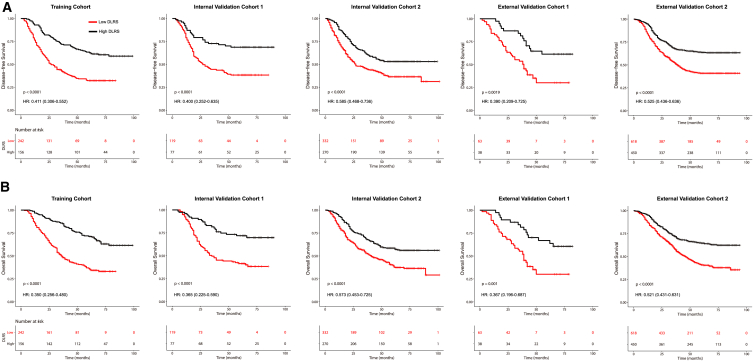
Kaplan-Meier analyses of disease-free survival (DFS) and overall survival (OS) according to the DLRS in patients with gastric cancer (A) Disease-free survival. (B) Overall survival. Training cohort (n = 398), internal validation cohort 1 (n = 196), internal validation cohort 2 (n = 602), external validation cohort 1 (n = 101), and external validation cohort 1 (n = 1,068).

We assessed the prognostic value of the DLRS. In the training cohort, the 5-year disease-free survival (DFS) and overall survival (OS) were 21.1% and 28.5% for the low-DLRS group; by contrast, the 5-year DFS and OS were 48.9% and 54.0% for the high-DLRS group (Figure 3). Similarly, in the internal validation cohort 1, the 5-year DFS and OS were 21.9% and 24.8% for the low-DLRS group compared with 48.7% and 55.5% for the high-DLRS group (Figure 3). We further confirmed significantly different prognoses for patients stratified by the DLRS in all internal and external validation cohorts (Figure 3).

We performed multivariate Cox regression analysis adjusting for clinicopathological variables. The DLRS remained an independent prognostic factor for predicting DFS and OS in the training and validation cohorts (Tables S4 and S5). We performed additional analyses within subgroups of patients stratified by various clinicopathological risk factors. Of note, patients with high DLRS had improved DFS and OS compared with patients with low DLRSs within each stage I, II, III, or IV (Figure S16). In addition, when stratified by other factors such as tumor size, location, histology, and differentiation, the DLRS remained a statistically significant prognostic factor in these subgroups (Figures S17 and S18). These data show that the DLRS is a strong independent prognostic factor in GC.

### DLRS predicts benefit from adjuvant chemotherapy

We further evaluated predictive value of the DLRS regarding the benefit of adjuvant chemotherapy after surgery in stage II and III patients. To do this, we first performed propensity score matching to ensure that the characteristics of patients who received chemotherapy were similar to those who did not (Tables S6). We then compared the survival for patients who either received or did not receive chemotherapy according to the DLRS.

**Figure 4:**
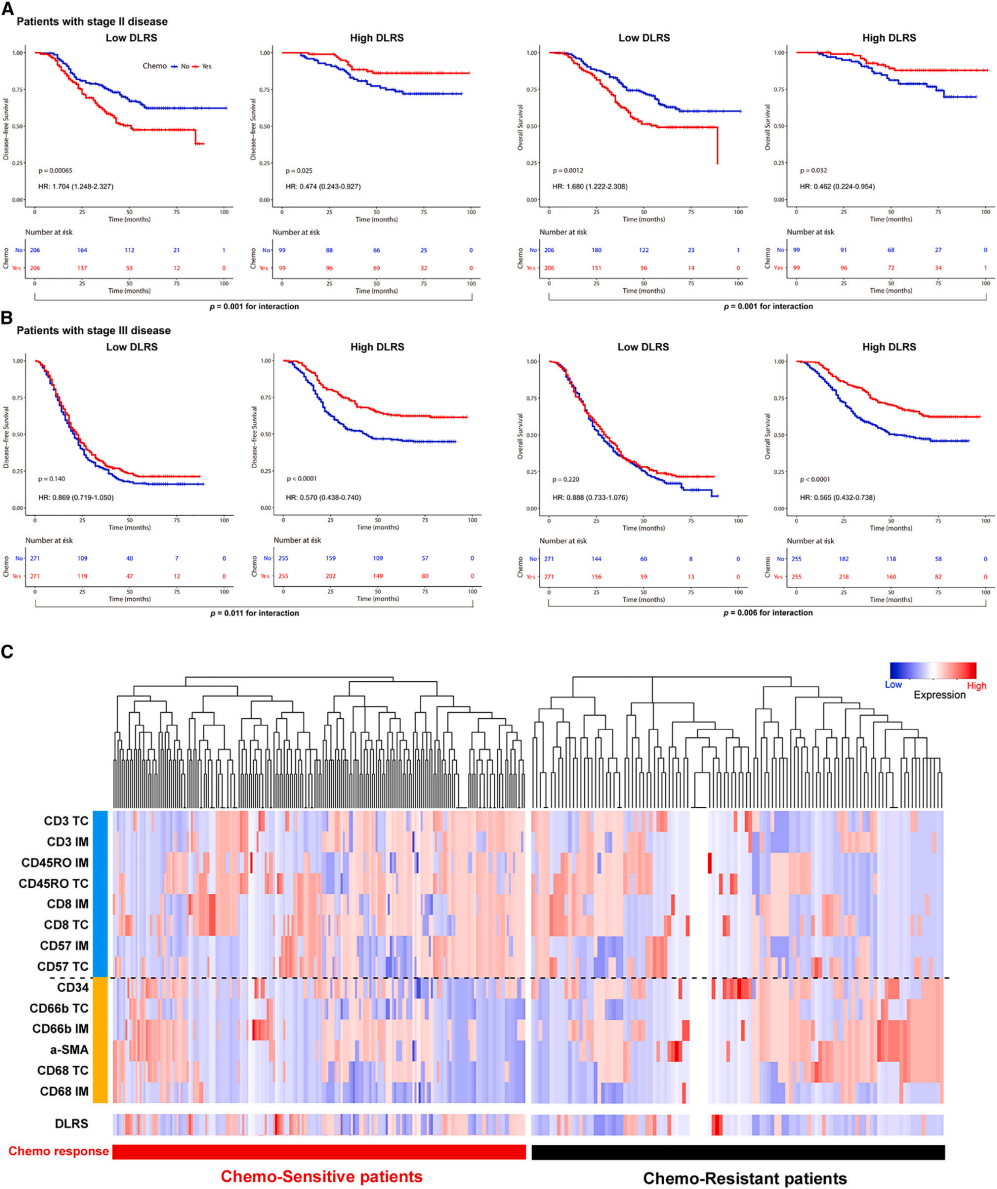
Relationship between the DLRS and DFS in matched patients who were treated with or without adjuvant chemotherapy (A) Stage II (n = 610). (B) Stage III (n = 1,052). Patients were stratified by the receipt of adjuvant chemotherapy. Statistical interaction tests were performed for the following: (left panel) predicted DLRS low vs. high and adjuvant chemotherapy: p_interaction_ = 0.001 and 0.011 for stage II and stage III; (right panel) predicted DLRS low vs. high and adjuvant chemotherapy: p_interaction_ = 0.001 and 0.006 for stage II and stage III. (C) Hierarchical tree structure classifying the stage II and III patients who received chemotherapy according to the levels of DLRS and 14 TME features: high expression (red) and low expression (green).

We found that for patients in the high-DLRS group, adjuvant chemotherapy was associated with an improved prognosis for both stage II and III disease, e.g., for DFS, stage II: hazard ratio (HR) 0.474 (95% CI, 0.243–0.927), p = 0.025, and stage III: HR 0.570 (0.438–0.740), p < 0.001 (Figures 4A and 4B). On the other hand, for patients in the low-DLRS group, adjuvant chemotherapy was not associated with an improvement in DFS in either stage II or III disease. In fact, for stage II patients, chemotherapy was associated with a significantly worse DFS (HR = 1.704 [95% CI, 1.248–2.327], p = 0.007) in the low-DLRS group. A statistical interaction test was performed between the DLRS signature and chemotherapy, which confirmed a significant interaction effect (p < 0.05) regarding the impact on DFS and OS. Additionally, we performed the above analyses using all the patients without propensity score matching and obtained similar results (Figure S19). We also performed multivariate logistic regression analysis and found that DLRS is an independent factor for predicting chemotherapy sensitivity in GC (Table S7). These data suggest that the DLRS may be predictive of the benefit from adjuvant chemotherapy in stage II and III disease.

**Figure 5:**
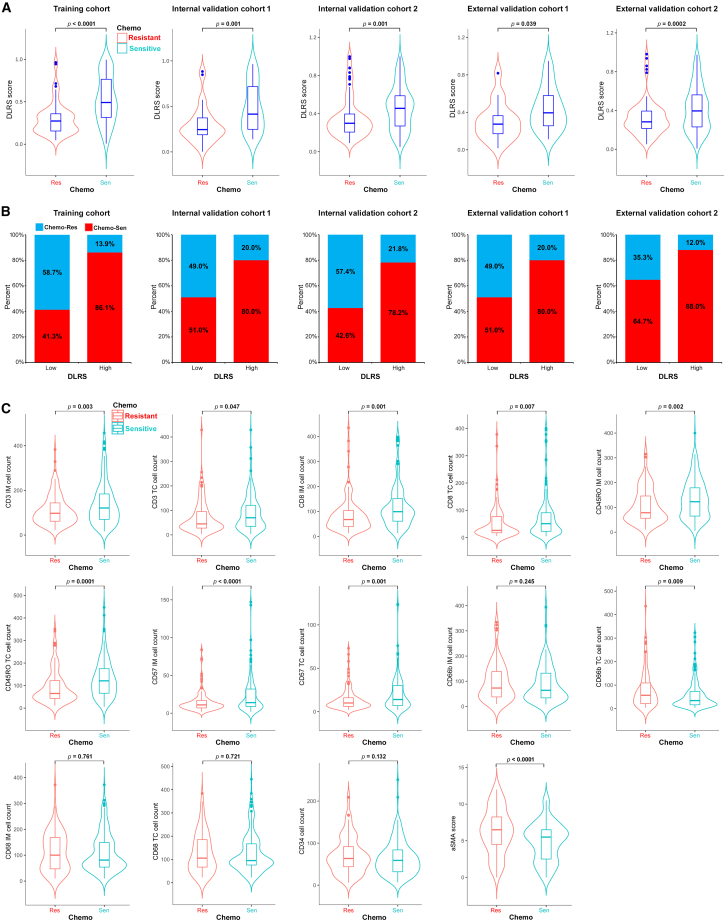
Relationship between the DLRS and chemotherapy response and TME characteristics (A) Violin plot showing DLRS scores in stage II and III patients resistant or sensitive to adjuvant chemotherapy. (B) Rate of clinical response (resistant, sensitive) to adjuvant chemotherapy in high- or low-DLRS score groups. (C) Violin plot showing TME features in stage II and III patients resistant or sensitive to adjuvant chemotherapy.

For the stage II and III patients treated with chemotherapy (n = 881), we divided them into two groups, chemo-sensitive vs. chemo-resistant, based on if they derived or did not derive a survival benefit from chemotherapy (DFS ≥ or <2 years). Chemo-sensitive patients had significantly higher DLRS scores than chemo-resistant patients in the training and validation cohorts (Figure 5A). Consistently, patients with high DLRS scores had a significantly higher probability of being chemo-sensitive than those with low DLRS scores, and vice versa (Figure 5B). We further explored the association between chemotherapy responsiveness and individual TME features (Figures 4C and 5C). Chemo-sensitive GC had a higher expression of lymphocytes such as CD3IM, CD3TC, CD8IM, and CD45ROTC (Figure 5C). On the other hand, chemo-resistant GC had a higher expression of fibroblasts and neutrophils (a-SMA, CD66bTC, and CD66bIM) (Figure 5C).

### DLRS predicts response to anti-PD-1 immunotherapy

**Figure 6:**
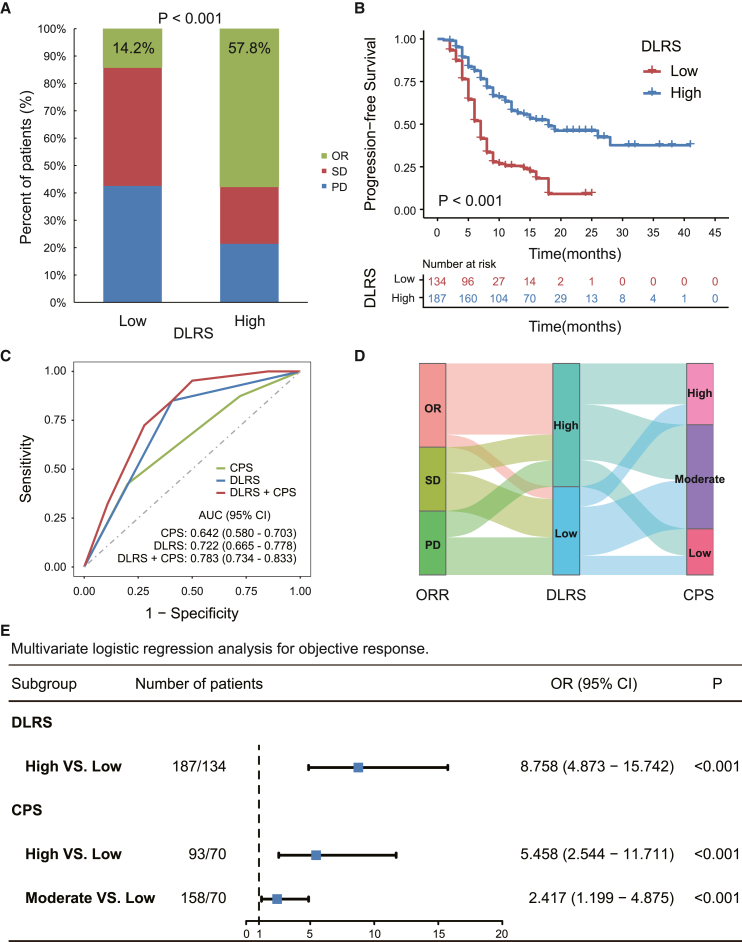
Relationship between the DLRS and clinical response and outcomes in patients treated with anti-PD-1 immunotherapy (A) Response rates in patients of the DLRS-high vs. -low groups. (B) Progression-free survival in patients of the DLRS high vs. low groups. (C) ROC curves of the predicted TME classes, CPS, and composite models combining TME classes and CPS for predicting immunotherapy response (n = 321); AUC: DLRS vs. CPS, p = 0.04; DLRS+CPS vs. CPS, p < 0.0001; DLRS+CPS vs. DLRS, p < 0.0001. (D) Alluvial diagram of the correspondence among patients classified according to the immunotherapy response, DLRS, and CPS in the merged immunotherapy cohorts (n = 321). (E) Forest plot for the multivariate logistic regression analysis for objective response. AUC, area under the receiver operator characteristic curve; CPS, combined positive score of PDL1 expression; OR, objective response (complete and partial response); SD, stable disease; PD, progressive disease.

We finally investigated relationships between the DLRS and the response to anti-PD-1 immunotherapy in a cohort of 321 patients with advanced GC. The overall objective response rate was 39.6%. Patients in the high-DLRS group achieved a substantially higher objective response rate (57.8%) compared with those in the low-DLRS group (14.2%) (Figure 6A). For all patients, the median progression-free survival (PFS) was 10 months. Kaplan-Meier analysis showed that the DLRSs were significantly associated with PFS (p < 0.001; Figure 6B). The median PFSs were 18 and 7 months in patients in the high- and low-DLRS groups, respectively.

Although the combined positive score (CPS) of PD-L1 expression, a clinically approved biomarker of immunotherapy response, was also associated with objective response (Figure S20A), the predictive accuracy was quite modest, with an AUC of 0.642 (95% CI, 0.580–0.703) (Figures 6C and 6D). In comparison, the DLRSs showed a higher accuracy for predicting objective response (AUC: 0.722 [0.665–0.778]; Figure 6C). In multivariate regression analysis, DLRSs had a stronger effect on objective response than CPS (Figure 6E). Importantly, DLRSs can further distinguish patients with differential response within the CPS-moderate and CPS-high subgroups (Figure S20B), suggesting complementary value between the two. Therefore, we combined CPS and DLRS into an integrative model (Figure 6C), which significantly improved the accuracy for immunotherapy response prediction (AUC: 0.783 [0.734–0.833], p < 0.0001) compared with CPS. Furthermore, we performed the subgroup analysis in 83 patients who were treated with single-agent immunotherapy, and observed similar results for predicting response to immunotherapy (Figure S21).

### Molecular correlates of the DLRS

**Figure 7:**
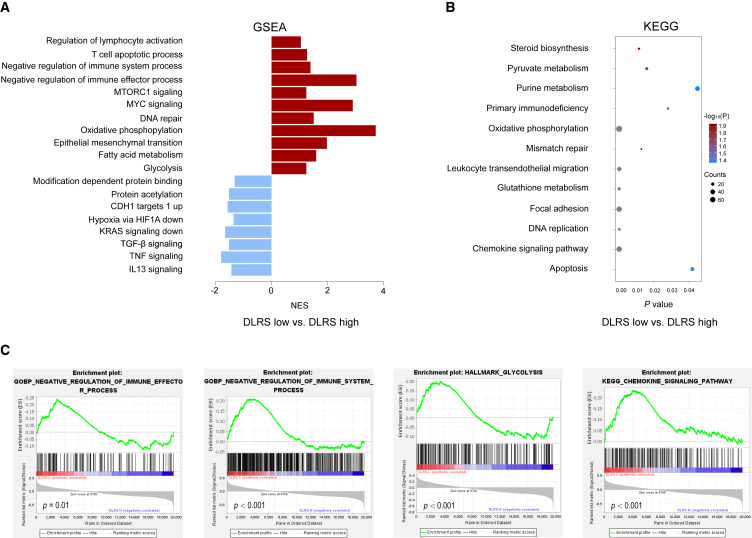
Molecular correlates of the DLRS in gastric cancer (A) Bar plot shows the top enriched molecular pathways by normalized enrichment score (NES) in the DLRS-high group (blue) and the DLRS-low group (red). A positive NES score indicates the pathway is significantly enriched in the DLRS-low group, and a negative NES indicates the pathway is significantly enriched in the DLRS-high group. (B) Bubble plot shows the top enriched pathways by gene counts along with p values. (C) Examples of the enrichment plot for the molecular pathways significantly associated with the DLRS.

We performed radiogenomics analysis to investigate the biological underpinnings of the DLRS. For this analysis, we leveraged the TCGA/TCIA-STAD dataset, which contains publicly available genomic/transcriptomic data and matched CT images for 42 patients with GC. We processed the CT images to compute DLRSs and performed gene set enrichment analyses to identify the underlying molecular pathways associated with the DLRSs. This analysis showed that tumors in the DLRS-low group were significantly enriched for multiple cancer hallmark-related pathways such as MYC signaling, KRAS signaling, and epithelial mesenchymal transition that are associated with aggressive tumor phenotypes (Figure 7A). In addition, tumors in the DLRS-low group were also enriched for immune suppression-related pathways such as negative regulation of immune system and effector processes as well as chemokine signaling that may play a role in the migration of myeloid-derived suppressor cells into the tumor (Figures 7A and 7C). Interestingly, we also found several metabolism-related pathways, including glycolysis, oxidative phosphorylation, and fatty acid metabolism pathways, associated with DLRS-low tumors (Figures 7B and 7C). Overall, these findings are consistent with their unfavorable prognosis and response rates and might suggest potential therapeutic targets for overcoming resistance to immunotherapy.

Finally, we assessed the relationship between the DLRSs and established biomarkers of immunotherapy response including TMB, PD-L1, GZMB, and T effector signatures. We did not find any significant associations between DLRSs and these existing biomarkers, suggesting that DLRSs might provide additional information for predicting immunotherapy response (Figure S22).

## Discussion

In this study, we developed and validated an imaging signature to noninvasively assess the TME in GC by quantitative analysis of CT images. Further, we demonstrated prognostic value of the imaging signature, which was independent of traditional clinicopathologic risk factors. Importantly, we showed that the imaging signature could identify which patients will benefit from adjuvant chemotherapy as well as improve the prediction of response to immunotherapy.

There is growing evidence for the prognostic and predictive relevance of TME, which has been established as a key determinant of treatment response and outcomes in many cancers. However, major challenges remain for the reliable evaluation of TME. In addition to the need for high-quality tissue, which is often limited in clinical practice, the current histological approach is prone to sample errors due to intratumor spatial heterogeneity and dynamic evolution of the TME.

Radiological imaging provides some unique advantages that may overcome these challenges. Imaging allows noninvasive evaluation and longitudinal monitoring of the entire tumor *in situ*. Because radiological phenotypes are fundamentally driven by the underlying pathophysiology, quantitative imaging analysis may reveal subtle relations between the two.^22^ The feasibility of this idea has been demonstrated in previous studies.^12^^,^^23^^,^^24^^,^^25^ Sun et al. developed a radiomic signature of CD8 T cells, which was correlated with clinical response and outcomes of patients treated with anti-PD1 immunotherapy.^12^ In our recent work, we developed a deep learning-based imaging signature of tumor stroma, which predicted prognosis and the benefit of adjuvant chemotherapy in GC.

Our study represents a clinical and technical advance over prior work in several aspects. First, previous studies have primarily focused on tumor-infiltrating lymphocytes,^12^^,^^26^ which only provide a simplified and partial view of the TME. Here, we evaluated both lymphoid/myeloid immune cells as well as stromal and vascular components that better capture the complexity and heterogeneity of the TME across patients. We note that the ground truth for TME status was based on IHC staining of large surgical specimens, which is more representative of the tumor than a small biopsy. Second, in deriving the imaging signature, we combined the respective merits of knowledge-based radiomics and data-driven deep learning approaches and showed superior performance over either approach. Third, we validated the prognostic and predictive values of our model in large multi-institutional cohorts of patients treated with chemotherapy and immunotherapy.

The standard treatment for localized GC includes surgery followed by adjuvant chemotherapy to prevent disease recurrence and improve survival. However, some studies have reported that certain subgroups of patients may not benefit from adjuvant chemotherapy.^2^^,^^3^^,^^27^^,^^28^ The optimal criteria for selection of candidates for adjuvant chemotherapy remain controversial. In our study, we found that only stage II/III patients classified as high DLRS were able to derive survival benefit from adjuvant chemotherapy, whereas patients classified as low DLRS did not benefit. In future clinical trials, novel personalized approaches to deintensify or intensify treatment based on the risk profile and TME status for these patients could be tested to enhance the efficacy of systemic therapies.

There is an unmet need for reliable biomarkers to identify which patients will respond to immunotherapy, which has become a standard treatment for many cancer types. We showed that the DLRS could predict response to anti-PD-1 immunotherapy in advanced GC. Specifically, tumors with high immune cell infiltration showed good response to immunotherapy. However, tumors with high stroma and vasculature had poor response. This is consistent with previous findings based on molecular approaches to TME evaluation.^29^ The imaging signature of TME had a stronger predictive effect than PD-L1 expression, a clinically approved biomarker of immunotherapy response. Importantly, combining the TME classifier with PD-L1 expression significantly improved the accuracy for response prediction. Although tumors with mismatch repair deficient (dMMR)/microsatellite instability-high (MSI-H) status have a much higher response rate, our model could identify a subset of tumors that do not respond to anti-PD-1 immunotherapy, and combination treatment strategies will be required for these patients.

Rather than studying the relation between radiological imaging and TME, an alternative approach is to apply radiomic or deep-learning analysis to directly predict treatment response or outcome, and many studies have reported promising results in gastric and other cancers.^30^^,^^31^^,^^32^^,^^33^^,^^34^^,^^35^ However, a critical issue has been the lack of biological interpretation of these imaging signatures. By contrast, our work builds on the extensive evidence for the well-established role of the TME in disease progression and its impact on treatment response,^36^^,^^37^^,^^38^^,^^39^ thus providing the biological rationale behind the model predictions.

### Limitations of the study

Our study is retrospective, which makes it susceptible to selection biases. To address this issue, we included large independent cohorts of patients from multiple institutions to validate our findings. Second, the decision about use of chemotherapy was made by the clinicians and/or patients, and thus the predictive effect of our imaging model should be validated in prospective randomized trials. Third, patients were enrolled from one geographic region in China; the distribution of clinical and pathological characteristics might be different in other populations.

Future work will focus on prospective validation of the imaging signature to confirm the generalizability and reproducibility in larger populations and across different scanners. While imaging will not replace tissue-based evaluation for TME, we envision that it could be used as an adjunct tool (like liquid biopsy for tumor genomics) to supplement current histopathology approaches where tumor tissue is unavailable or inadequate.

In conclusion, we developed a noninvasive radiological model for the assessment of TME status using deep learning and radiomics analysis of CT images. The imaging signature has the potential to refine prognosis and guide personalized therapy of GC. Prospective studies and randomized trials are required to confirm its clinical validity and clinical utility.

## STAR★Methods

### Key resources table

**Table:** 

REAGENT or RESOURCE	SOURCE	IDENTIFIER
**Antibodies**		

CD3	NeoMarkers	clone SP7
CD8	NeoMarkers	clone SP16
CD45RO	Invitrogen	clone UCHL1
CD57	NeoMarkers	clone NK1
CD66b	BD Pharmingen	Cat#555723
CD68	Dako,	clone PG-M1
CD34	Abcam,	ab81289
a-SMA	Abcam,	ab5694
Horseradish-peroxidase-conjugated anti-rabbit and anti-mouse antibody	Dako	Code K5007

**Software and algorithms**		

R 4.1.0	The R Project for Statistical Computing	https://www.r-project.org
GraphPad PRISM 8	GraphPad software	https://www.graphpad.com/
BioRender	BioRender website	https://www.biorender.com/
SPSS software version 21.0	IBM SPSS software	https://www.ibm.com/spss
Python version 3.6	Python software	https://www.python.org/

### Resource availability

#### Lead contact

Further information and requests for resources and reagents should be directed to and will be fulfilled by the lead contact, Ruijiang Li (rli2@stanford.edu).

#### Materials availability

This study did not generate new unique reagents.

### Experimental model and subject details

#### Study design and patients

The overall study design is shown in Figure 1. This study followed the Transparent Reporting of a Multivariable Prediction Model for Individual Prognosis or Diagnosis (TRIPOD) reporting guideline. Ethical approval was obtained from the institutional review boards of Nanfang Hospital of Southern Medical University and Sun Yat-sen University Cancer Center (SYSUCC), and patient consent was waived for this retrospective analysis. We retrospectively reviewed data for 5,213 patients who underwent surgery for gastric cancer in two academic medical centers. The inclusion criteria were: histologically confirmed diagnosis of GC; resection of the primary tumor with at least 15 lymph nodes harvested; preoperative abdominal computed tomography (CT) images available; and complete clinicopathological and follow-up data available. We excluded patients who had other synchronous malignant neoplasms, or previously received neoadjuvant chemotherapy; patients whose primary tumor could not be identified on CT were also excluded.

A total of 2,686 patients in six independent cohorts were included in this study (Figure S1; Tables S1 and S2).). The training cohort and two internal validation cohorts included 398, 196, and 602 patients who were consecutively treated at Nanfang Hospital of Southern Medical University (Guangzhou, China) from January 1 2005 to June 30 2009, from July 1 2009 to December 31 2012, and from January 1 2013 to June 30 2017 respectively. The two external validation cohorts included 1,169 patients consecutively treated at SYSUCC (Guangzhou, China) between June 1 2007 and June 30 2013. Here, we divided patients into training and validation cohorts by time of surgery instead of random sampling. This strategy substantially reduces arbitrariness in data splitting, which allows more rigorous assessment and independent validation of the model. Moreover, this approach mimics the situation where a model is first trained on existing data and then tested on future patients.

Clinicopathologic data including age, gender, tumor and lymph node status, tumor differentiation, Lauren histology type, carcinoembryonic antigen (CEA), and cancer antigen 19-9 (CA19-9) was collected. D2 lymph node dissection was performed in most patients (>90%) in accordance with clinical guidelines.^40^^,^^41^ Tumor staging was performed on the basis of the 8^th^ Edition of the American Joint Committee on Cancer TNM Staging Manual.^42^ There were 164 (51.10%), 109 (44.0%), 258 (49.5%), and 525 (46.8%) patients who received 5-fluorouracil–based adjuvant chemotherapy in the training cohort, internal validation cohorts 1 and 2 from Nanfang Hospital, and external validation cohort from SYSUCC, respectively. Among these patients, 171 (32.2%) from Nanfang Hospital and 179 (34.1%) from SYSYCC received the XELOX (capecitabine–oxaliplatin) chemotherapy regimen, while 360 (67.8%) and 346 (65.9%) received the FOLFOX (fluorouracil–folinic acid–oxaliplatin) regimen in the two institutions.

The immunotherapy cohort consists of 321 patients with advanced GC treated at Nanfang Hospital and Guangdong Provincial Hospital of Chinese Medicine (Table S2). Anti-PD-1 drugs include: Nivolumab, Pembrolizumab, or Toripalimab. Clinical data, including patient demographics, treatment information, laboratory and pathologic examinations, and CT scans were acquired. Microsatellite instability (MSI) status was assessed by either IHC or DNA sequencing.

### Method details

#### Immunohistochemistry (IHC) staining and scoring

Formalin-fixed paraffin-embedded (FFPE) samples were cut into 4-μm thick sections, which were then processed for immunohistochemistry as previously described.^3^^,^^7^ The samples were de-waxed in xylene and rehydrated in decreasing concentrations of ethanol. Prior to staining, the sections were subjected to endogenous peroxidase blocking in 1% H_2_O_2_ solution diluted in methanol for 10 min and then heated in a microwave for 30 min with 10 mmol/L citrate buffer (pH 6.0). Serum blocking was performed using 10% normal rabbit serum for 30 min. The slides were incubated overnight with an antibody against human immune cell biomarkers (CD3 (pan T cells), CD8 (cytotoxic T cells), CD45RO (memory T cells), CD45RA (naive T cells), CD57 (natural killer cells), CD68 (macrophages), CD66b (neutrophils)), a microvascular marker (CD34), and a stromal marker (a-SMA) at 4°C, followed by incubation with an amplification system with a labeled polymer/HRP (EnVision, DakoCytomation, Denmark) at 37°C for 30 min. The reaction was visualized using diaminobenzidine (DAB)+ chromogen, and nucleus was counterstained using hematoxylin. In all assays, we included negative control slides with the primary antibodies omitted. Every staining run contained a slide of positive control. And all slides were stained with DAB dyeing for the same time for each antibody (Table S8).

As previously described,^7^ we calculated a machine learning classifier to assess the overall TME status based on the expression of eight IHC markers, including CD3 IM, CD3 TC, CD8 IM, CD45RO TC, CD57 IM, CD68 TC, CD66b IM and CD34 (IM: invasive margin, TC: tumor core). Patients were classified into two groups: TME-high vs. TME-low.

The IHC markers were evaluated independently by two gastrointestinal pathologists who were blinded to the clinical data. A third pathologist was consulted to reach a consensus when different opinions arose between the two primary pathologists. In detail, the tissue sections were screened at low power (100×) using an inverted research microscope (model DM IRB; Leica, Germany), and 5 most representative fields were selected. The density of immune cells was measured at 200× magnification for two respective areas at tumor core (TC) and invasive margin (IM). The nucleated stained cells in each area were quantified and expressed as the number of cells per field. For micro-vessels, any discrete cluster or single cell stained positive for CD34 was counted as one micro-vessel.^9^^,^^43^ For the stromal marker (a-SMA), stain intensity was graded as 0 (negative staining), 1 (weak staining), 2 (moderate staining), and 3 (strong staining); stain extent was graded as 0 (0%–4%), 1 (5%–24%), 2 (25%–49%), 3 (50%–74%), and 4 (>75%).^44^^,^^45^ Values of the stain intensity and extent were multiplied and then averaged over the five fields as the final score.

#### CT acquisition and image processing

All patients underwent contrast-enhanced abdominal CT scans prior to surgery. Following intravenous contrast administration, arterial and portal venous-phase contrast-enhanced CT scans were performed after delays of 28 s and 60 s, respectively. Iodinated contrast material in the amount of 90–100 mL (Ultravist 370, Bayer Schering Pharma, Berlin, Germany) was injected at a rate of 3.0 or 3.5 mL/s with a pump injector (Ulrich CT Plus 150, Ulrich Medical, Ulm, Germany). The type of CT scanners included GE Lightspeed 16, GE Healthcare Milwaukee, WI; 64-section LightSpeed VCT, GE Medical Systems, Milwaukee, WI; USA. The CT acquisition protocols were as follows: 120 kV; 150–190 mAs; 0.5- or 0.4-s rotation time. Contrast-enhanced CT was reconstructed with a field of view, 350 × 350 mm; data matrix, 512 × 512; in-plane spatial resolution 0.607–0.75 mm; axial slice thickness, 1.25–7.5 mm.

CT images were resampled to a consistent spatial resolution of 0.75 × 0.75 × 2.5 mm by using trilinear interpolation. We normalized the CT intensity to a window of [-150, 150] HU to highlight the soft-tissue contrast. To focus analysis on the most relevant region (i.e., gastric carcinoma), we delineated the primary tumor as the region of interest.

CT images at the portal venous phase were analyzed given its better contrast. The primary tumor was delineated by two radiologists (C.C. and Q.Y. with 11 and 10 years of clinical experience in abdominal CT interpretation, respectively) using the ITK-SNAP software. Both radiologists reached consensus regarding tumor delineation.

#### Development of an imaging model to assess IHC-based TME classifier

We trained a deep learning radiomics model (named “HR-Rad-Net”) to predict the IHC-based TME classifier using CT images. The input image to the model consists of three channels: the original full CT image, CT image with tumor mask, and the binary tumor mask, all with a size of 64 × 64. To leverage the respective advantages of knowledge-based radiomics and data-driven deep learning approaches, we combined the two components in a unifying model (Figure S2A). This idea is motivated by prior work showing that either approach can extract useful information for assessing TME.^12^^,^^13^^,^^33^^,^^35^ The proposed model consists of two parts: feature representation learning via convolutional neural network and radiomic feature extraction. HR-Rad-Net draws on HR-Net’s ability to effectively learn reliable high-resolution representations. Here, we incorporate a squeeze and excitation (SE) module into HR-Net to better extract multi-scale image features (Figure S2B).^46^^,^^47^ The squeeze operation utilizes average pooling to extract the global information from each channel of the feature; while the excitation operation adopts a multi-layer perceptron to learn channel-wise weights to re-weight each channel.

For radiomics analysis, we computed a total of 361 radiomics features, including 9 shape features, 72 first-order statistical features, 96 Gray Level Cooccurrence Matrix (GLCM) features, 64 Gray Level Run Length Matrix (GLRLM) features, 64 Gray Level Size Zone Matrix (GLSZM) features, and 56 Gray Level Dependence Matrix (GLDM) features.^48^ Then, the radiomics information of the CT image and annotation information was calculated and embedded into the feature space via a full connection layer. Given the prohibitive cost of manually delineating full 3D tumor contours for over 2600 patients in the study, these radiomics features were computed based on the image slice containing the largest tumor size.

The network learned features and radiomics features are fused in a fully connected layer to generate the final prediction. Both types of features are standardized by the *Z* score method before being fed into the fully connected layer. To demonstrate effectiveness of the proposed approach, we performed ablation experiments and compared the performance with the models trained without radiomics or SE module separately. Finally, to investigate and visualize which areas in the image are important for prediction, we used the gradient-weighted class activation mapping approach.^49^

#### Model architecture and training

We propose an HR-Radiomics-Net to predict the TME status of gastric tumors from CT images. The proposed model consists of two parts: feature representation learning via convolutional neural network and radiomic feature extraction, which are fused in a fully connected layer to generate the final prediction (Figure S2). We used a cohort of 398 patients from Nanfang Hospital, Southern Medical University, China for training purposes.

To reduce overfitting, we applied data augmentation and learning rate decay. Specifically, the augmentation included image reflection along the patient’s anterior/posterior or left/right directions and random rotation with an angle sampled from (−30°, +30°). We used focal loss as the objective function which is defined as:

$$\text{FL}\left(p_{t}\right)=-\alpha {\left(1-p_{t}\right)}^{\gamma }\log\left(p_{t}\right)$$

where $p_{t}$ is the output of the network, α,γ are set to balance the positive and negative samples.

In this study, we set α to 1 and γ to 2. The batch size was set to 16, the learning rate was set to 1e-3 and the learning rates decay half per 10 epochs. The optimizer used for training was the Adam algorithm. The proposed HR-Radiomics-Net was implemented on the open source Pytorch platform and trained using an NVIDIA GTX 1080TI.

#### Visualization and interpretation of network prediction

After training the network, we visualize the heatmap of the network that shows the more important features of the image for the model prediction. To achieve this, we use the Guided Grad-CAM.^49^ Guided Grad-CAM generates a corresponding heatmap of the input image, which indicates how much the position contributes to the classification. In addition, Guided Grad-CAM can also give a guide based on the returned gradient map, which provides a visualization of the network classification at a fine-grained level. (Figure S3)

#### Model validation and comparison with alternative methods

We tested the model performance in the internal and external validation cohorts. To demonstrate effectiveness of the proposed approach, we performed ablation experiments and compare the performance with the radiomics and deep learning module trained separately, as shown in Figures S5.

#### Accuracy of the imaging model for TME classification

We evaluated the accuracy of the CT imaging-based model to assess the TME classifier defined by IHC. Metrics including the area under the receiver operating characteristic curve (AUC), overall accuracy, sensitivity, and specificity were computed. The optimal cutoff value for the deep learning radiomics signature (DLRS) was determined using Youden’s index in the training cohort, which maximizes the sum of sensitivity and specificity. This analysis was performed for patients in the training cohort, internal validation cohort 1, and external validation cohort 1 for which IHC data was available. Calibration plots were used to graphically represent the agreement between the predicted and actual probability of the TME classifier. A decision curve analysis was performed to evaluate the model’s clinical usefulness by quantifying the net benefit at various threshold probabilities.

#### Imaging model’s association with prognosis and chemotherapy response

We assessed the imaging model’s association with survival outcomes including disease-free survival (DFS) and overall survival (OS). DFS was defined as the time from surgery to disease progression or death. OS was defined as the time to death from any cause. This analysis was performed for all 2,365 patients in 5 independent cohorts for which outcome data were available. Additionally, we evaluated the prognostic value in patient subgroups as defined by clinicopathological factors.

We further assessed the association between DLRS and adjuvant chemotherapy response in patients with stage II and III gastric cancer. To minimize potential selection bias and confounding effects, we used a matching strategy to balance patients in each DLRS-defined group. Propensity score matching (PSM) was performed for patients who received vs. did not receive chemotherapy using 1:1 nearest matching. The following variables were matched: age, gender, differentiation, CEA, CA19-9, location, depth of invasion (T stage), lymph node metastasis (N stage), tumor size, and Lauren type.

#### Imaging model’s association with immunotherapy response

We finally assessed the image model’s relation to clinical response and outcomes using an independent cohort of 321 advanced GC patients treated with anti-PD-1 immune checkpoint blockade. Response to immunotherapy was evaluated according to the irRECIST criteria^50^ and defined as complete response (CR), partial response (PR), stable disease (SD), or progressed disease (PD). Objective response was defined for patients who achieved either CR or PR. Progression-free survival (PFS) was calculated from the start of treatment until disease progression, death, or last follow up. The combined positive score (CPS) was defined as the total number of PD-L1 positive cells (tumor, lymphocytes, and macrophages) divided by the number of tumor cells. CPS was categorized as high (CPS≥10), intermediate (10>CPS≥1), and low (CPS<1).

#### Radiogenomics analysis

We used the TCGA/TCIA-STAD dataset for radiogenomics analysis, which contains publicly available genomic/transcriptomic data and matched CT images for 42 gastric cancer patients. We processed the CT images to compute DLRS and performed gene set enrichment analyses to identify the underlying molecular pathways associated with the DLRS. We identified genes that are significantly correlated with the imaging signature by the Spearman’s rank test. Multiple testing will be corrected using the Benjamini-Hochberg method. To elucidate the biological meaning of DLRS, we performed the gene set enrichment analysis (GSEA) and gene ontology (GO) kyoto encyclopedia of genes and genomes (KEGG) analyses using the GSEA software (version 4.1.0).^51^ All parameters were set to their default values, and an adjusted P-value of <0.05 was considered statistically significant.

We further assessed the relationship between the DLRS and established biomarkers of immunotherapy response using patients in the TCGA/TCIA dataset. The PD-L1 and GZMB expression values were determined based on the transcriptomic data from the TCGA. Tumor mutation burden (TMB) was computed using the R package “Matfools”.^52^ Furthermore, two well established T effector signatures were evaluated, namely, OAK-T effector signature (based on PD-L1, CXCL9, IFNG)^53^ and POPLAR-T effector signature (based on CD8A, GZMA, GZMB, IFNG, PD-L1, EOMES, CXCL9, CXCL10, and TBX21).^54^

### Quantification and statistical analysis

#### Statistical analysis

We compared two groups using the *t*-test for continuous variables and the chi-square test or Fisher exact test for categorical variables, as appropriate. Survival curves were generated according to the Kaplan-Meier method and compared using the log rank test. Univariate and multivariate analyses were performed using the Cox proportional hazards model. Interaction between the imaging model and adjuvant chemotherapy was assessed by means of the Cox model. Statistical analysis was conducted with R software (version 4.1.0) and SPSS software (version 21.0). A two-sided p value <0.05 was considered statistically significant.

## Data Availability

• The CT image data reported in this study cannot be deposited in a public repository because they contain sensitive information that could compromise patient privacy. Deidentified patient-level clinical and outcome data will be provided upon reasonable request. In addition, summary statistics describing these data have been deposited at supplementary tables and are publicly available as of the date of publication. • Source code for the deep learning model is available at: https://github.com/MontaEllis/HR-Rad-Net. • Any additional information required to reanalyze the data reported in this work paper is available from the lead contact upon request.

## Supplemental information

Document S1. Figures S1–S22

Table S1. Characteristics of patients in the training, internal, and external validation cohorts, related to Figure 1

Table S2. Characteristics of patients in the anti-PD1 immunotherapy cohort, related to Figure 1

Table S3. Clinical characteristics of patients according to the DLRS in the training, internal, and external validation cohorts, related to Figure 2

Table S4. Univariate association of DLRS and clinicopathological characteristics with disease-free and overall survival in the training, internal, and external validation cohorts, related to Figure 3

Table S5. Multivariate Cox regression analyses for disease-free survival and overall survival in patients with gastric cancer, related to Figure 3

Table S6. Clinical characteristics of patients according to the chemotherapy in stage II and III patients before and after PSM, related to Figure 4

Table S7. Multivariate analysis of DLRS and clinicopathological characteristics for association with chemotherapy response, related to Figure 5

Table S8. Antibody sources and staining conditions, related to Figure 2

Document S2. Article plus supplemental information
