# Supplementary material for: Non-invasive tumor microenvironment evaluation and treatment response prediction in gastric cancer using deep learning radiomics
Source: Cell Rep Med. 2023 Aug 8;4(8):101146. doi: 10.1016/j.xcrm.2023.101146 (PMC10439253; doi:10.1016/j.xcrm.2023.101146)
Supplement: Document S1. Figures S1–S22 [file mmc1.pdf]

Cell Reports Medicine, Volume *4*

Supplemental information

Non-invasive tumor microenvironment evaluation and treatment response prediction in gastric cancer using deep learning radiomics

Yuming Jiang, Kangneng Zhou, Zepang Sun, Hongyu Wang, Jingjing Xie, Taojun Zhang, Shengtian Sang, Md Tauhidul Islam, Jen-Yeu Wang, Chuanli Chen, Qingyu Yuan, Sujuan Xi, Tuanjie Li, Yikai Xu, Wenjun Xiong, Wei Wang, Guoxin Li, and Ruijiang Li

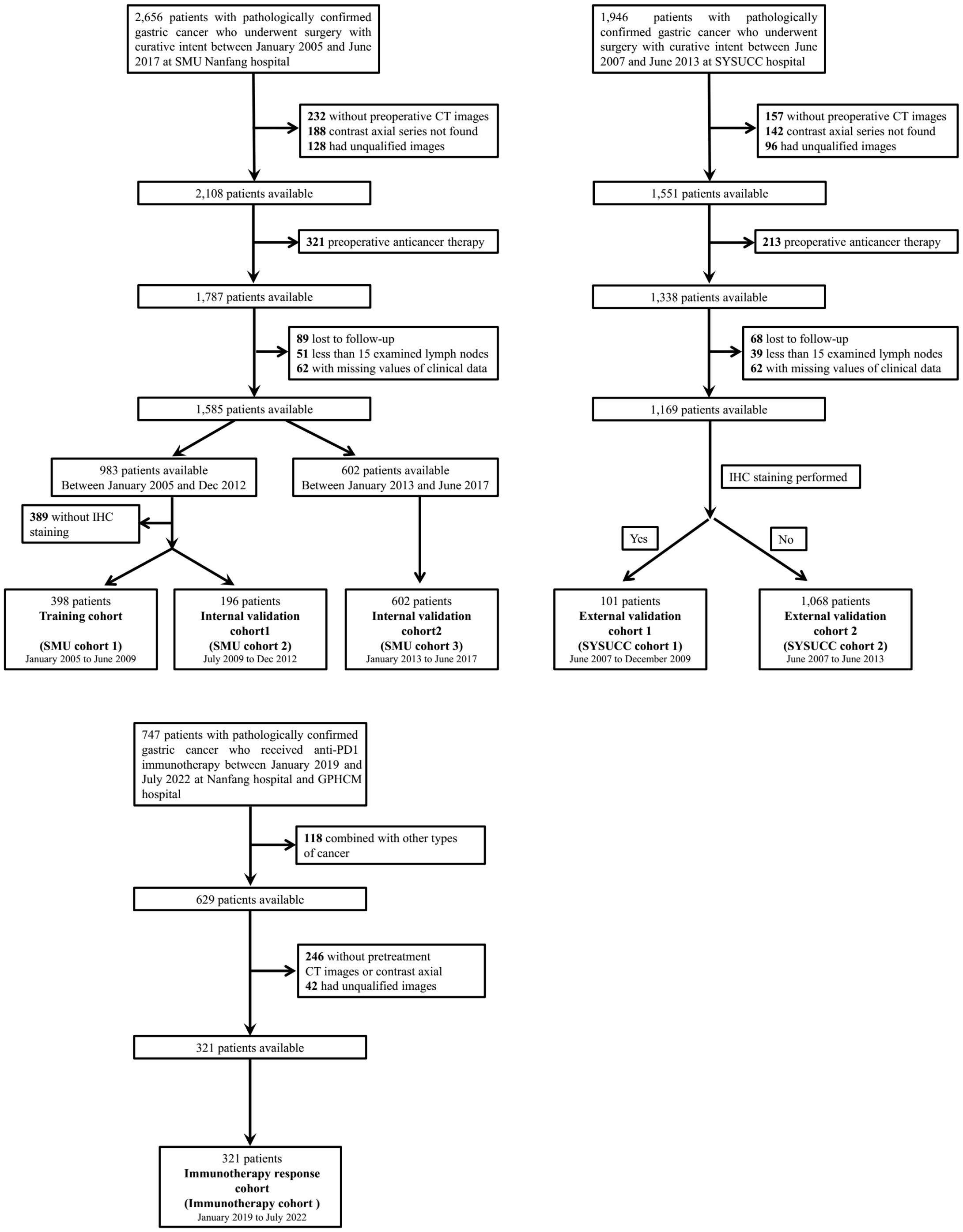

Figure S1. Flow chart of patient inclusion and exclusion. Related to Figure 1.

SMU cohorts: patients from Southern Medical University hospital; SYSUCC cohorts: patients from Sun Yat-sen University Cancer Center; Stanford cohort: patients from Stanford University hospital. Immunotherapy cohort: patients from Guangdong Provincial Hospital of Chinese Medicine (GPHCM).

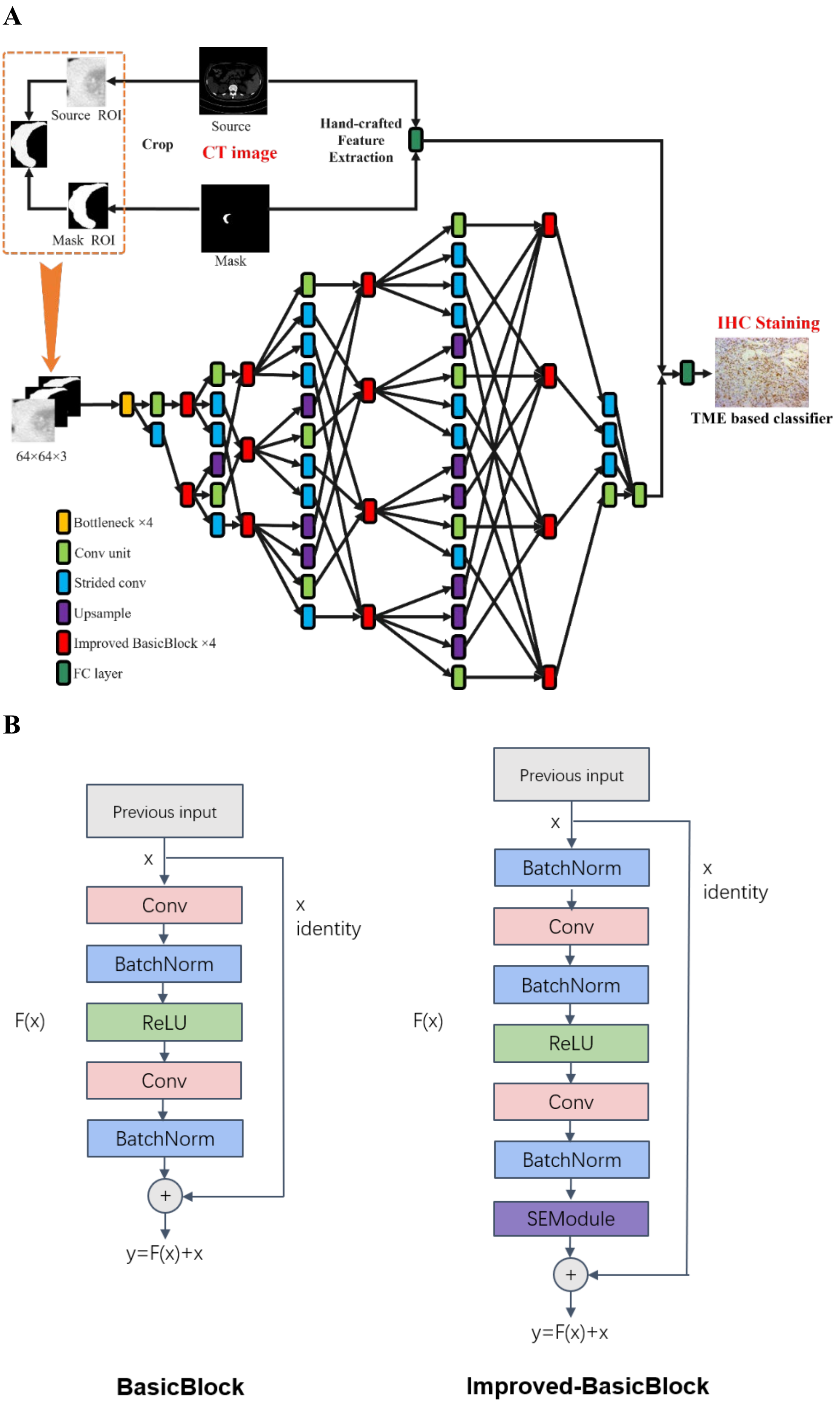

Figure S2. The framework of the proposed network (A) and comparison of basic residual network blocks, SE-based improved residual network blocks, and HR-Rad-Net approach (B). Related to Figure 1.

(A) The radiomics features extracted from the source and mask images are fused with the network

features extracted from improved HR-Net. Different colored, directional arrows represent different processing methods.

(B) The SE-based improvement module incorporates a channel attention mechanism, making the network more focused on learning implicit information between channels.

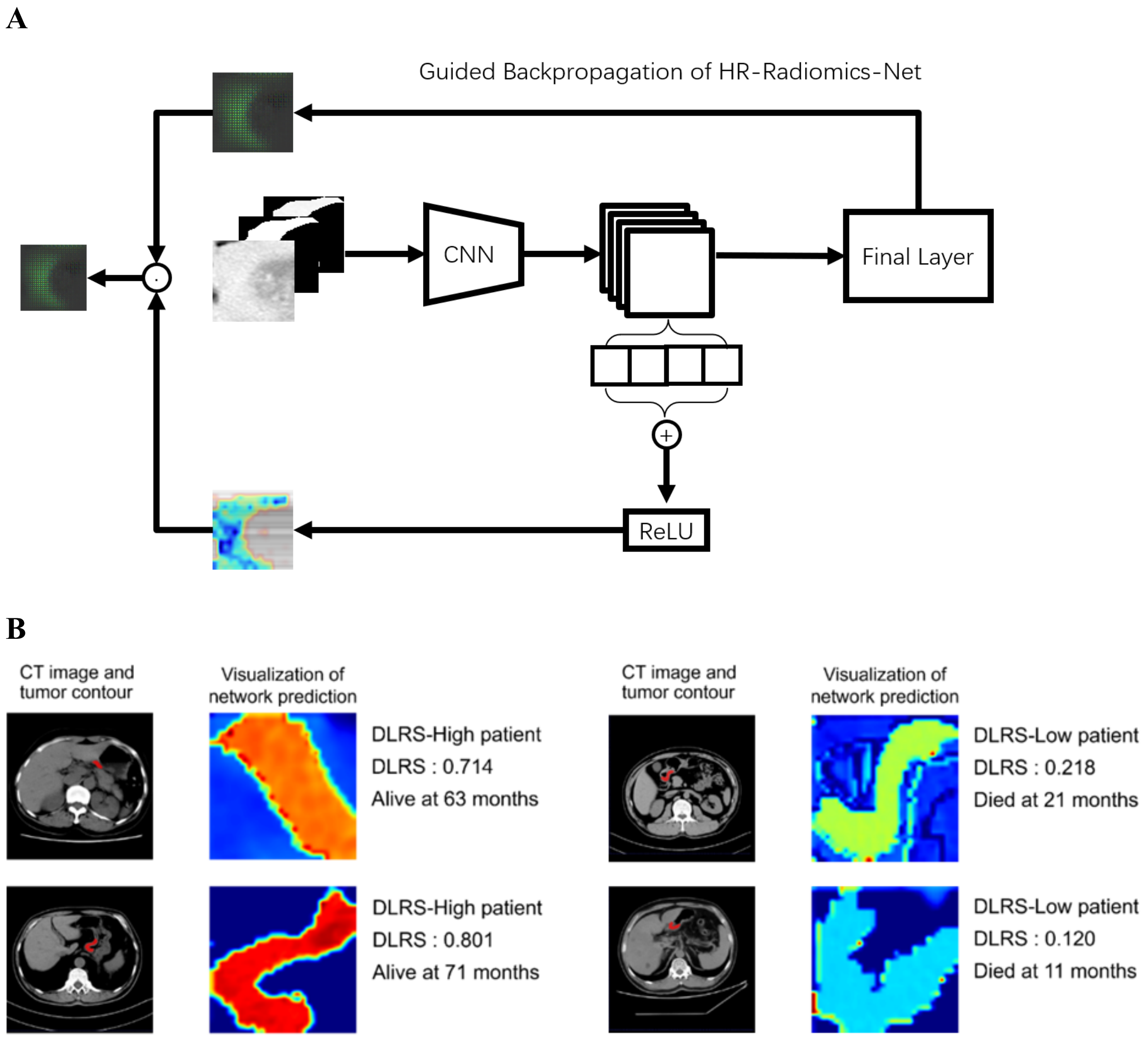

Figure S3. **Guided Grad-CAM for visualization of network prediction. Related to Figure 1.**

(A) The back-propagation of the HR-Radiomics-Net highlights certain areas of the feature map. These feature maps are combined to calculate heatmaps, which show the sub-regions of the network that are focused on learning. At the same time, we combine the guide map of the back-propagation and feature heatmaps to obtain a visualization of the Guided Grad-CAM.

(B) Examples of the CT images, visualization of the HR-Radiomics-Net prediction, and corresponding network prediction (DLRS). Areas in the CT image that are important for prediction of TME class were identified and highlighted by the Grad-CAM approach. Grad-CAM: gradient-weighted class activation mapping.

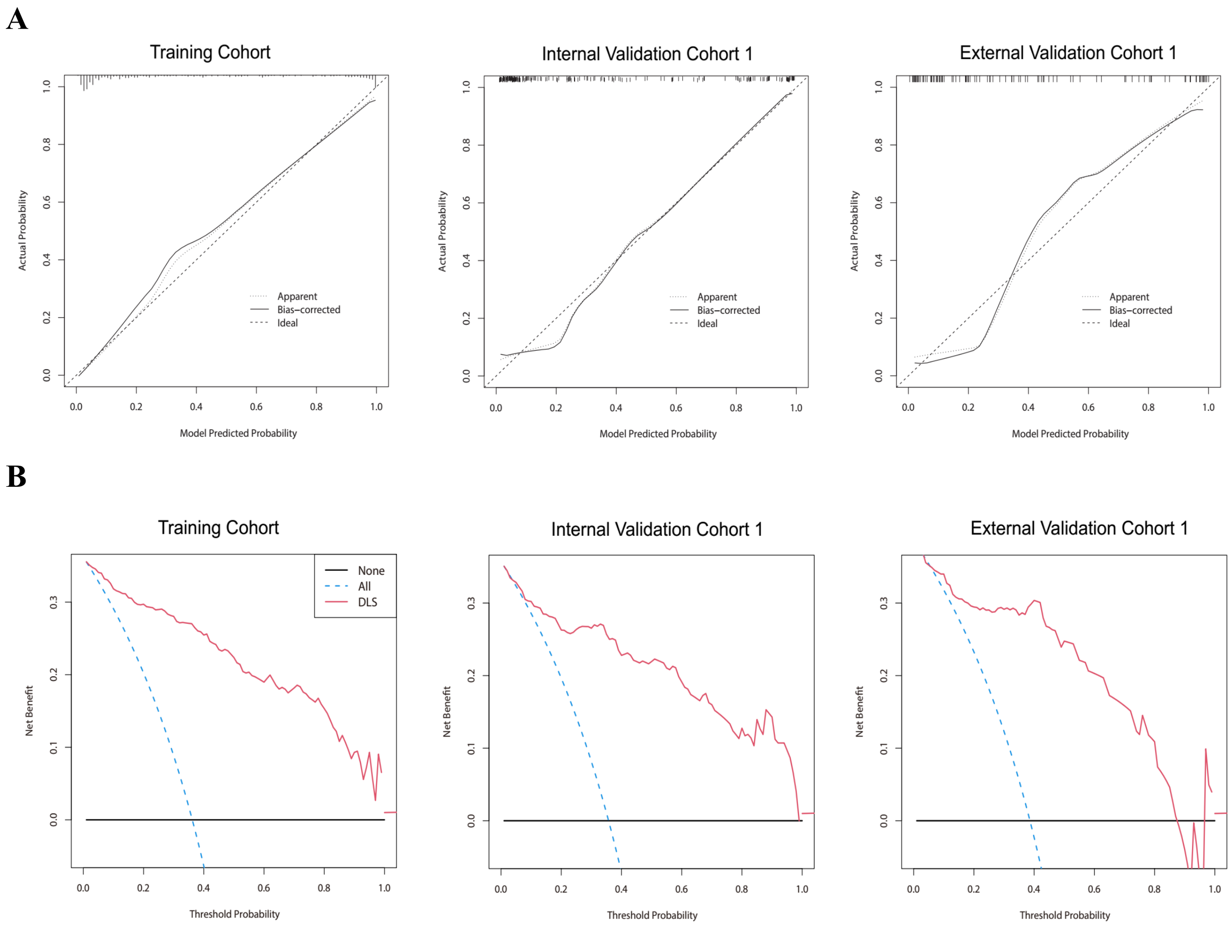

Figure S4. Calibration curves (A) and decision curve (B) analysis for the DLRS model. Related to Figure 2.

(A) The calibration showed the agreement between the estimated and the observed probabilities of the TME classifier. DLRS model-estimated probabilities is plotted on the x-axis; the actual probabilities is plotted on the y-axis. The diagonal dotted line is a perfect estimation by an ideal model, in which the estimated outcome perfectly corresponds to the actual outcome. The solid line is the performance of the DLRS model: a closer alignment with the diagonal dotted line represents a better estimation.

(B) Decision curve analysis of the DLRS model for predicting the TME classifier in the training and validation cohorts. The y-axis measures the net benefit, and the red line represents the DLRS model.

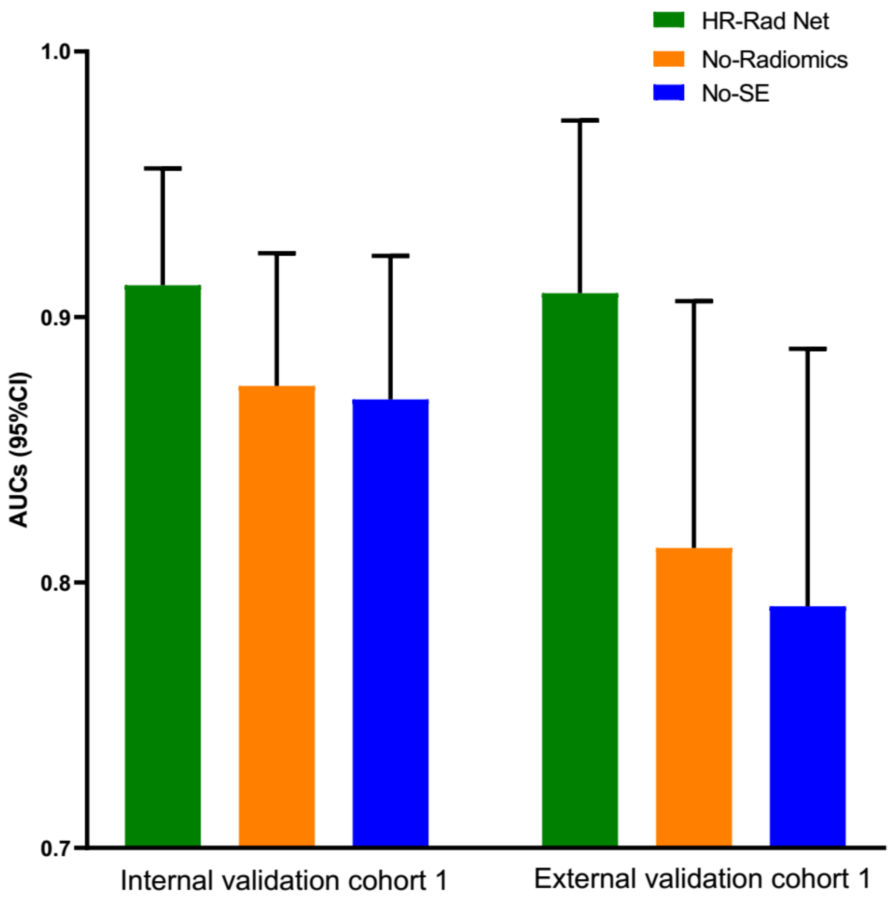

Figure S5. Comparison of the HR-Rad-Net approach with other approaches for prediction of TME-based classifier. Related to Figure 1. No-Radiomics: the model developed without combing with radiomics features. No-SE：the model developed without SE strategy.

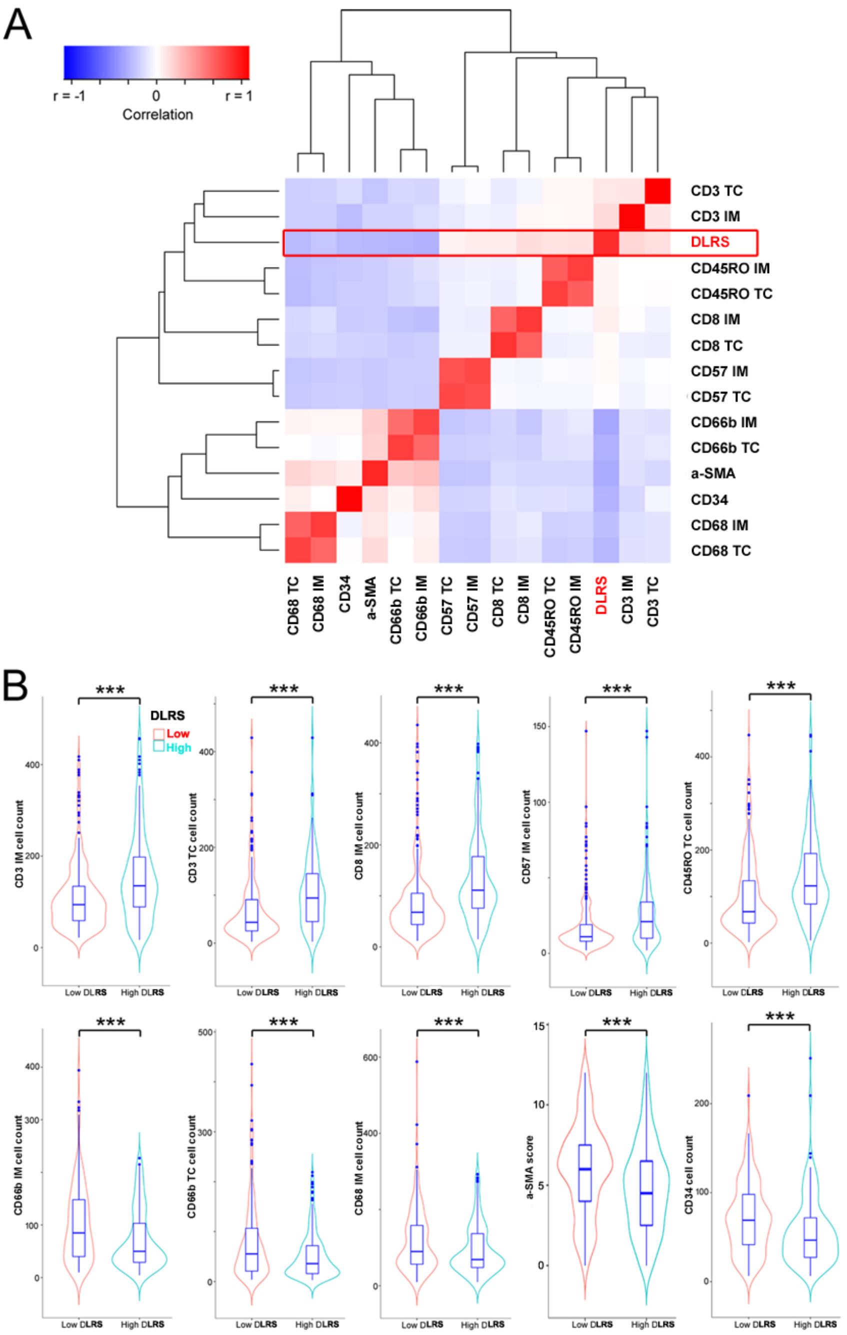

Figure S6. Relationship between the imaging model and individual TME features. Related to Figure 2.

(A) Pairwise correlation between the DLRS and TME features followed by unsupervised hierarchical clustering (Pearson uncentered algorithm).

(B)Violin plots showing association between DLRS group and TME features.

Training cohort

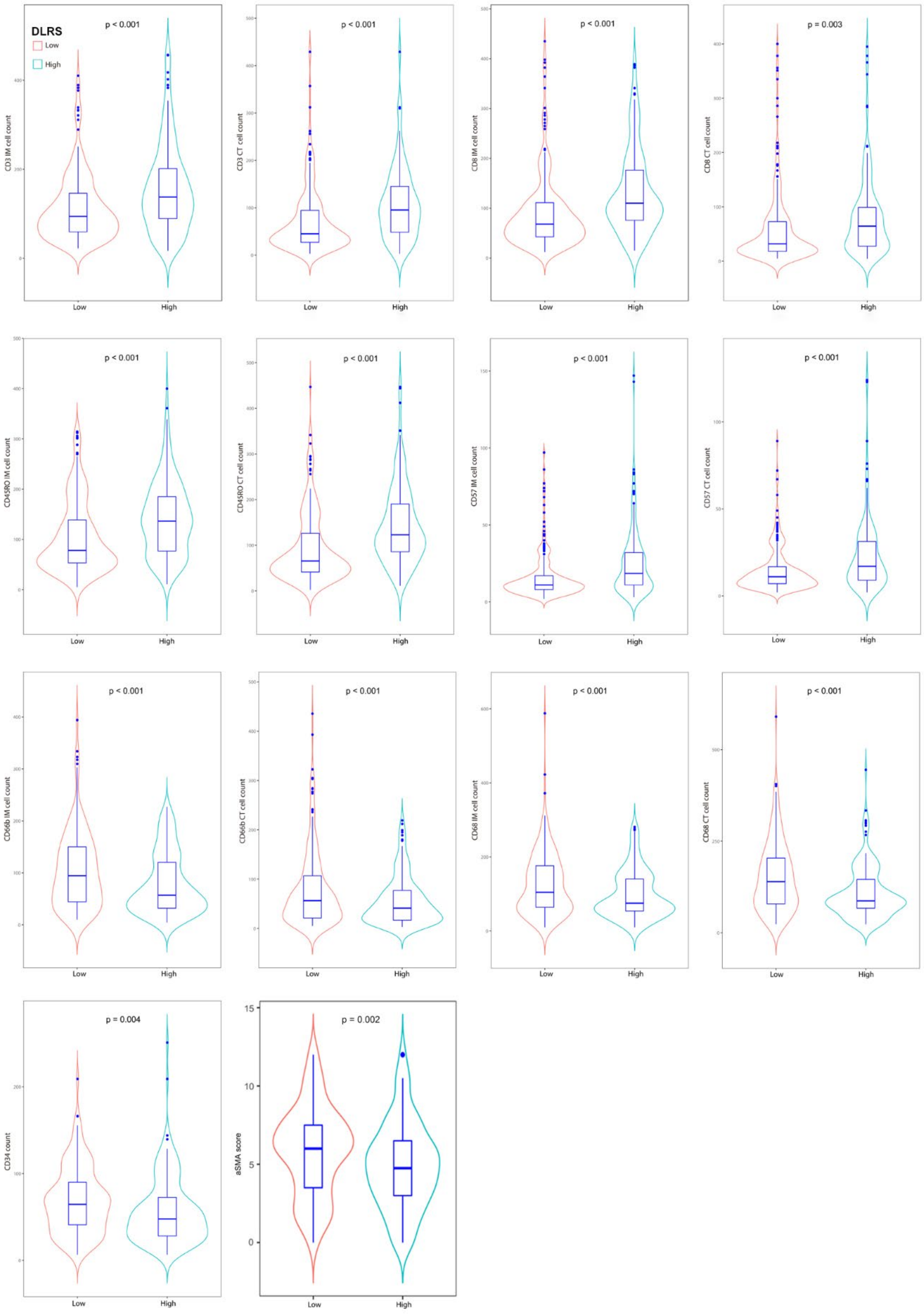

Figure S7. The relationship between DLRS and each TME features in the traing cohort. Related to Figure 2.

Internal validation cohort 1

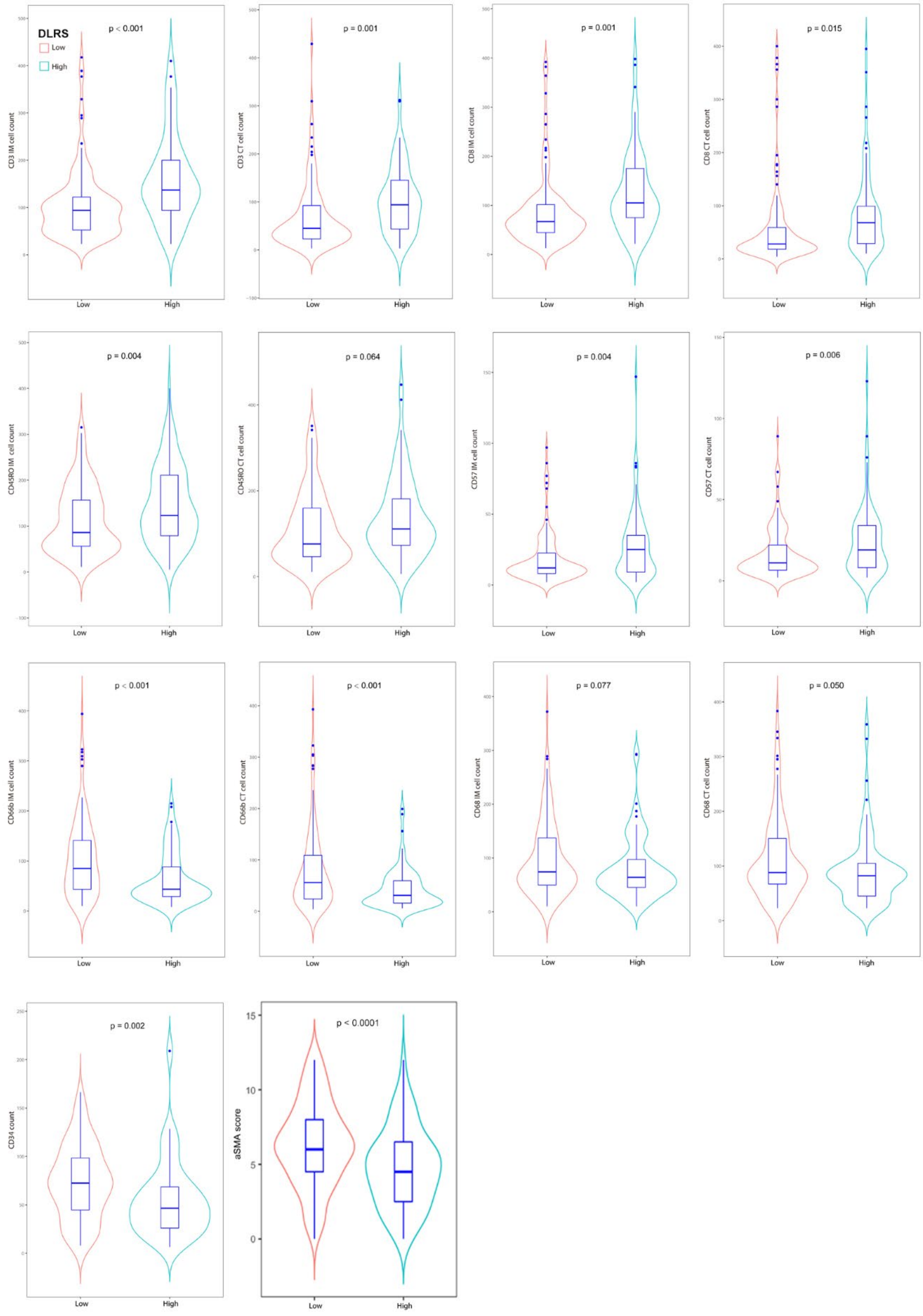

Figure S8. The relationship between DLRS and each TME features in the internal validation cohort 1. Related to Figure 2.

External validation cohort 1

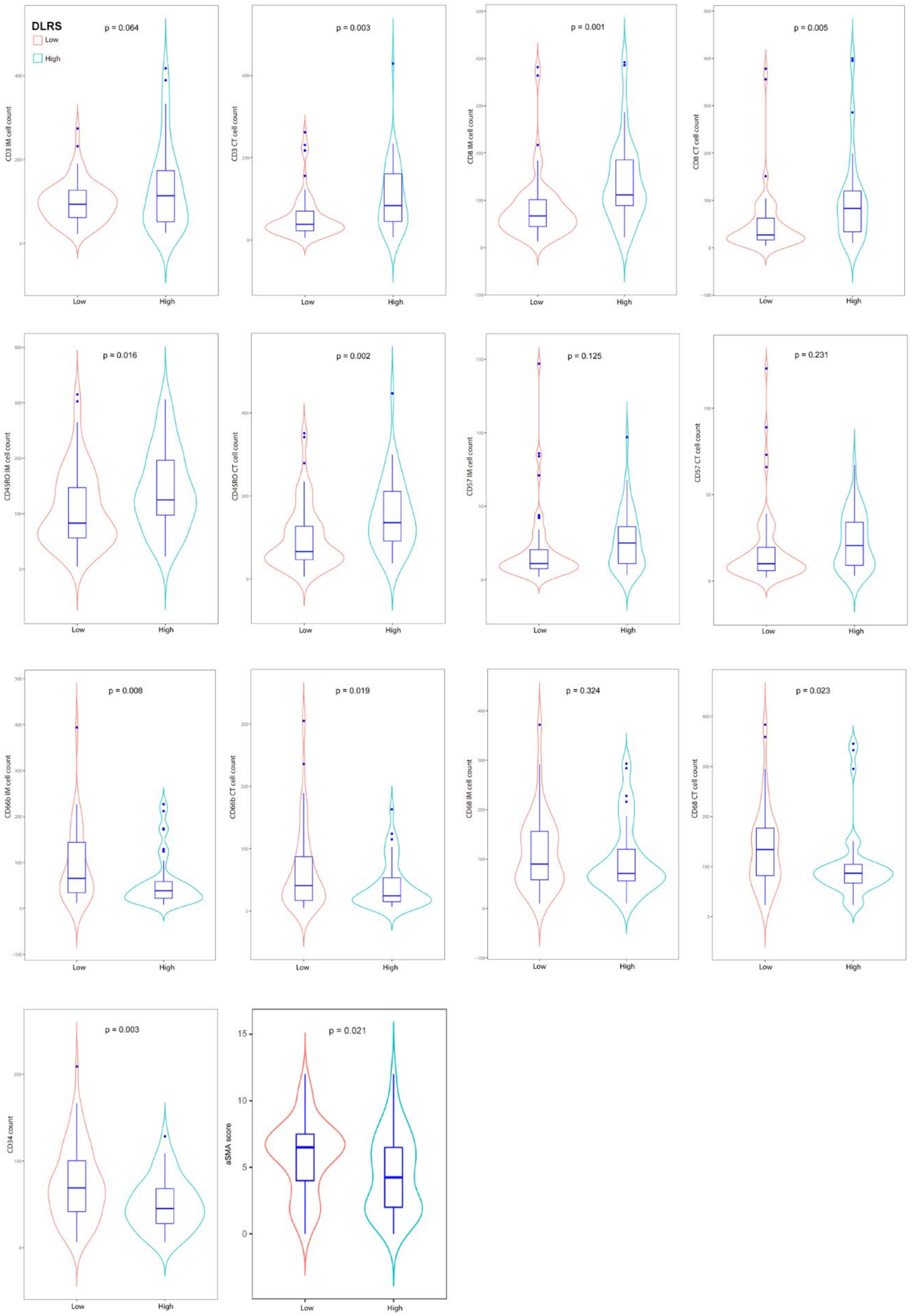

Figure S9. The relationship between DLRS and each TME features in the external validation cohort 1. Related to Figure 2.

Training cohort

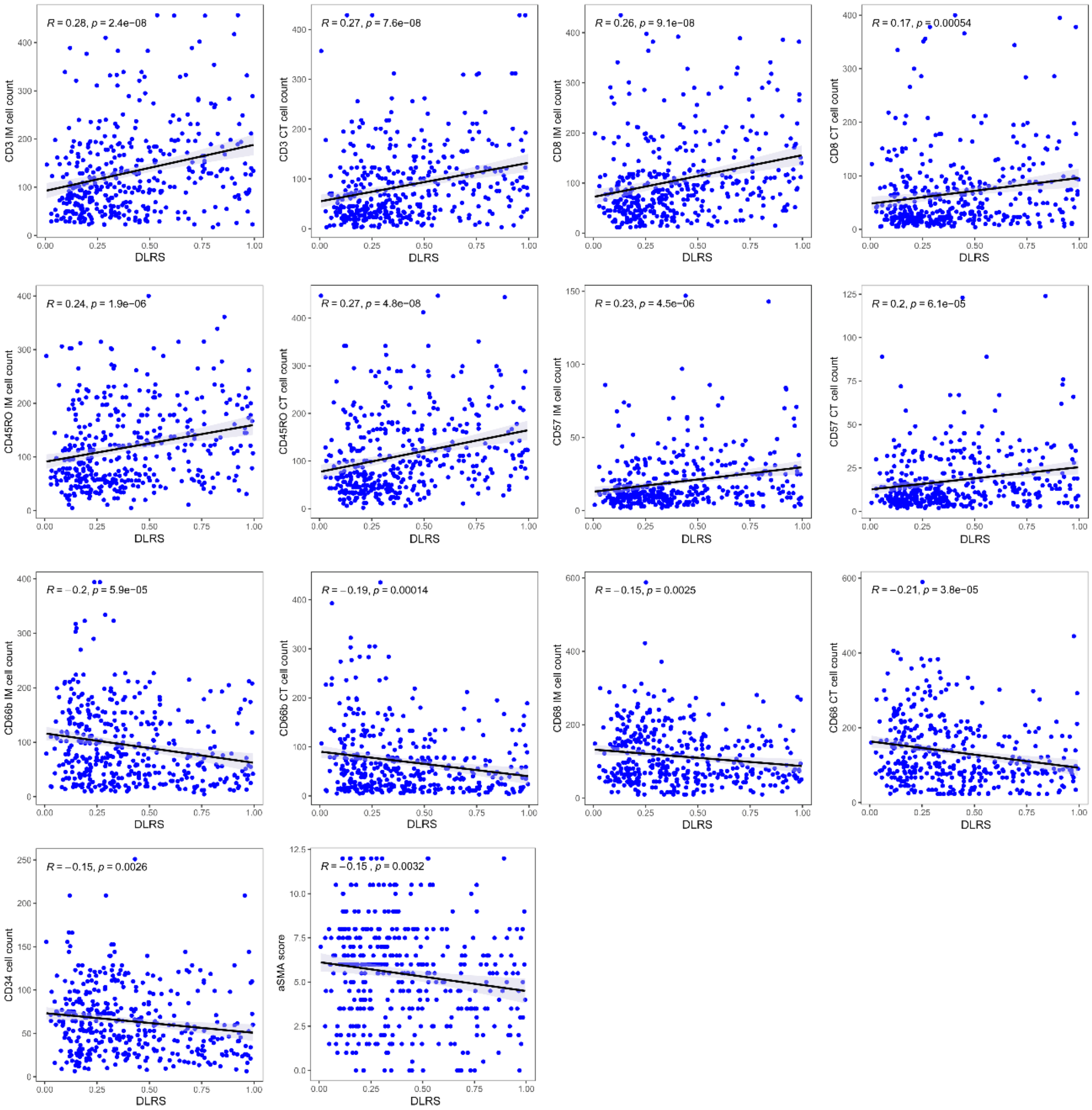

Figure S10. The scatter plot of relationship between DLRS and each TME features in the training cohort. Related to Figure 2.

Internal validation cohort 1

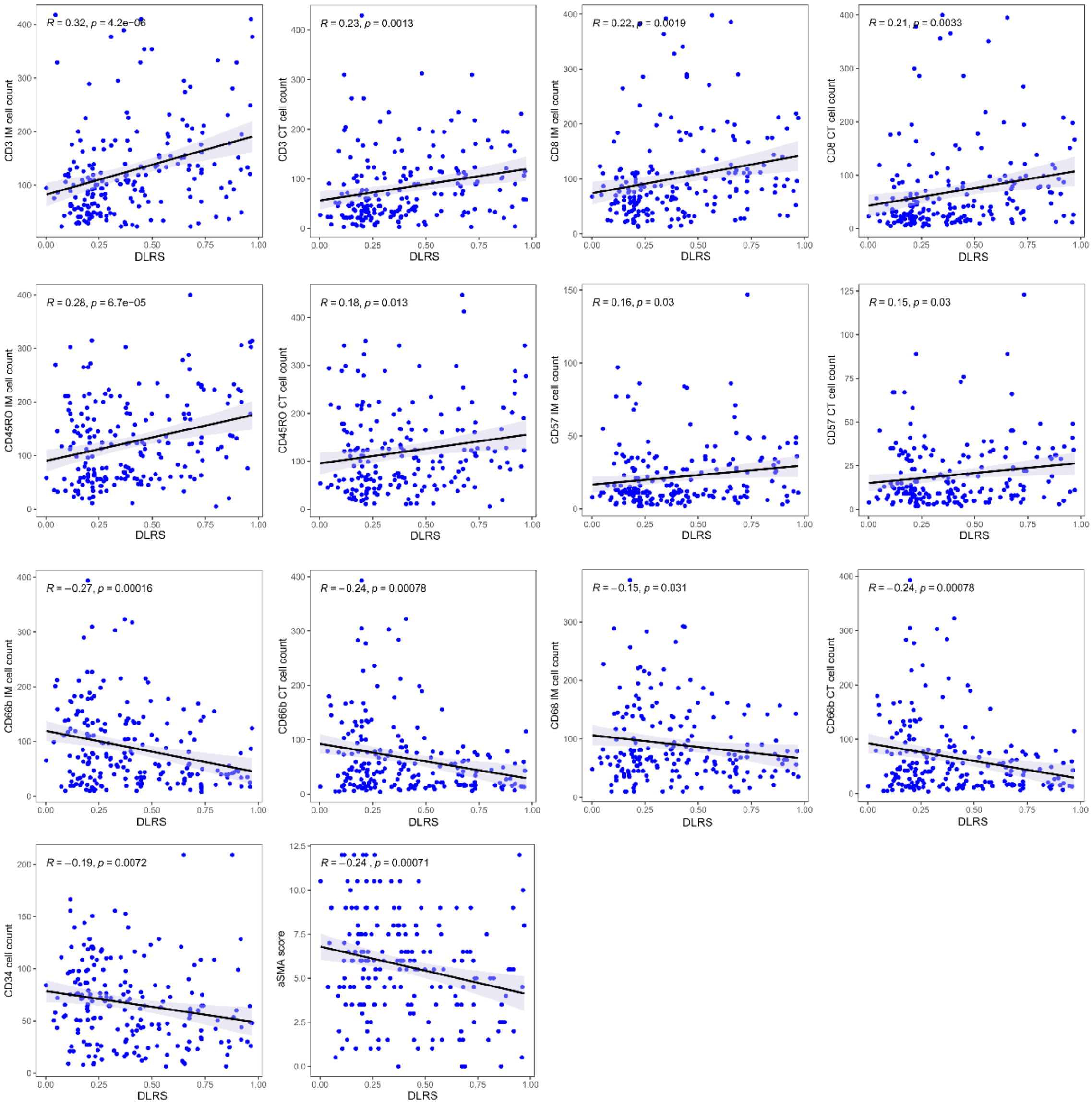

Figure S11. The scatter plot of relationship between DLRS and each TME features in the internal validation cohort 1. Related to Figure 2.

External validation cohort 1

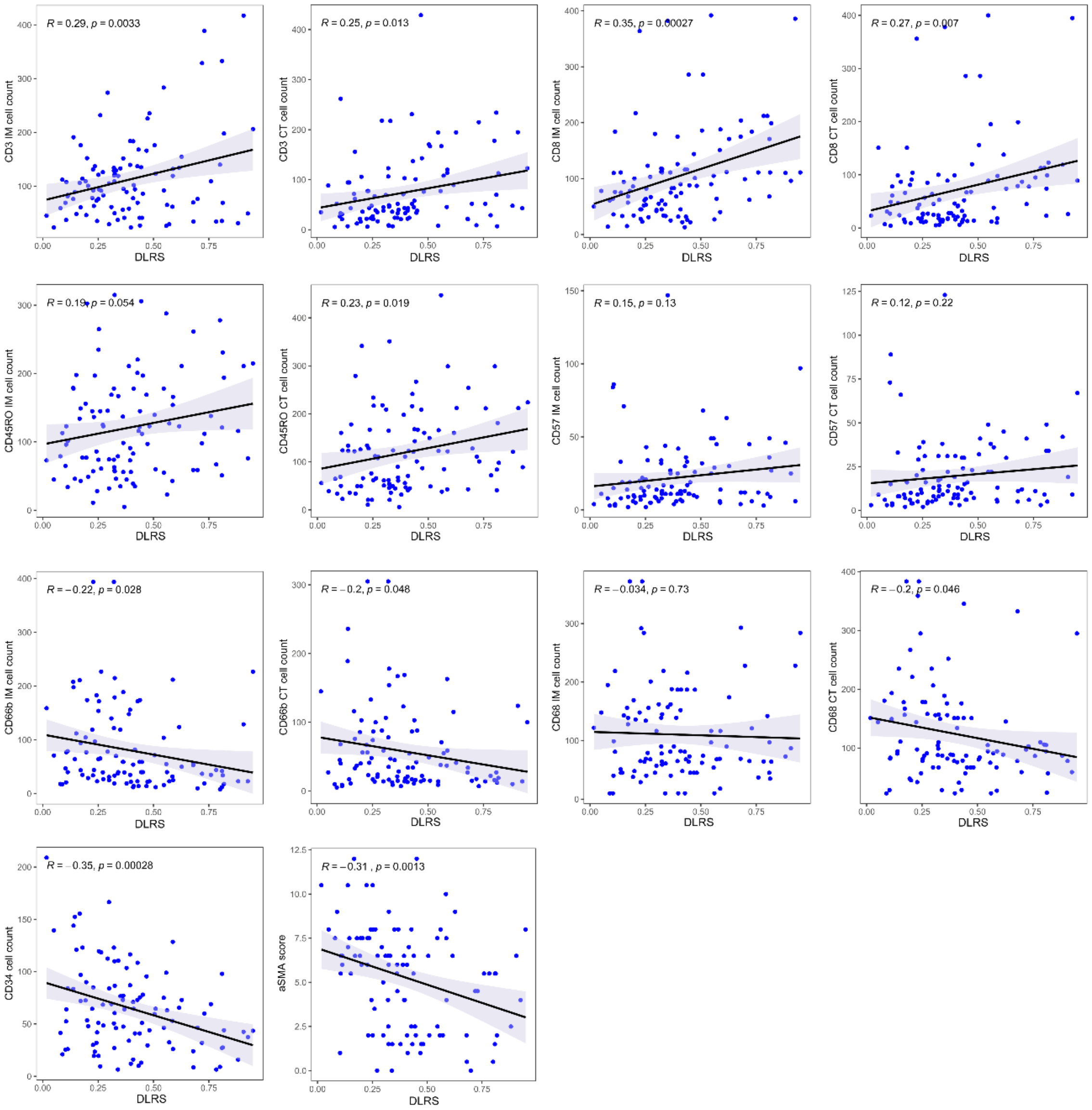

Figure S12. The scatter plot of relationship between DLRS and each TME features in the external validation cohort 1. Related to Figure 2.

Training cohort

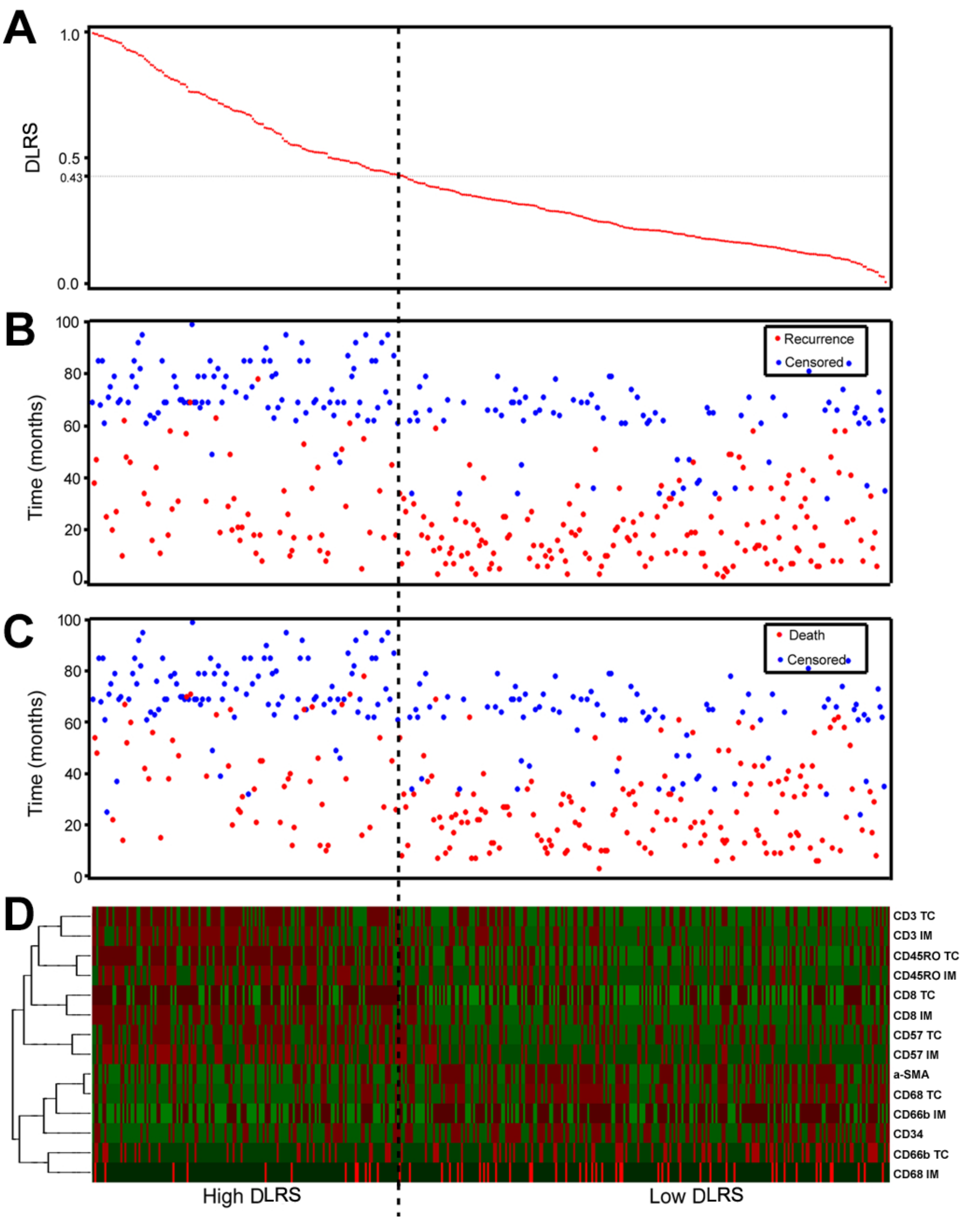

Figure S13. The deep learning score analysis of GC patients in the training cohort (n = 398). Related to Figure 3.

(A) DLRS score of GC patients. (B) Recurrence status of GC patients. (C) Survival status of GC patients. (D) Color-gram of the expression profiles of 14 tumor microenvironment features in GC patients. Rows represent 14 features and columns represent patients. Magenta dotted line represents the DLRS cutoff dividing the patients into high and low DLRS groups.

Internal validation cohort 1

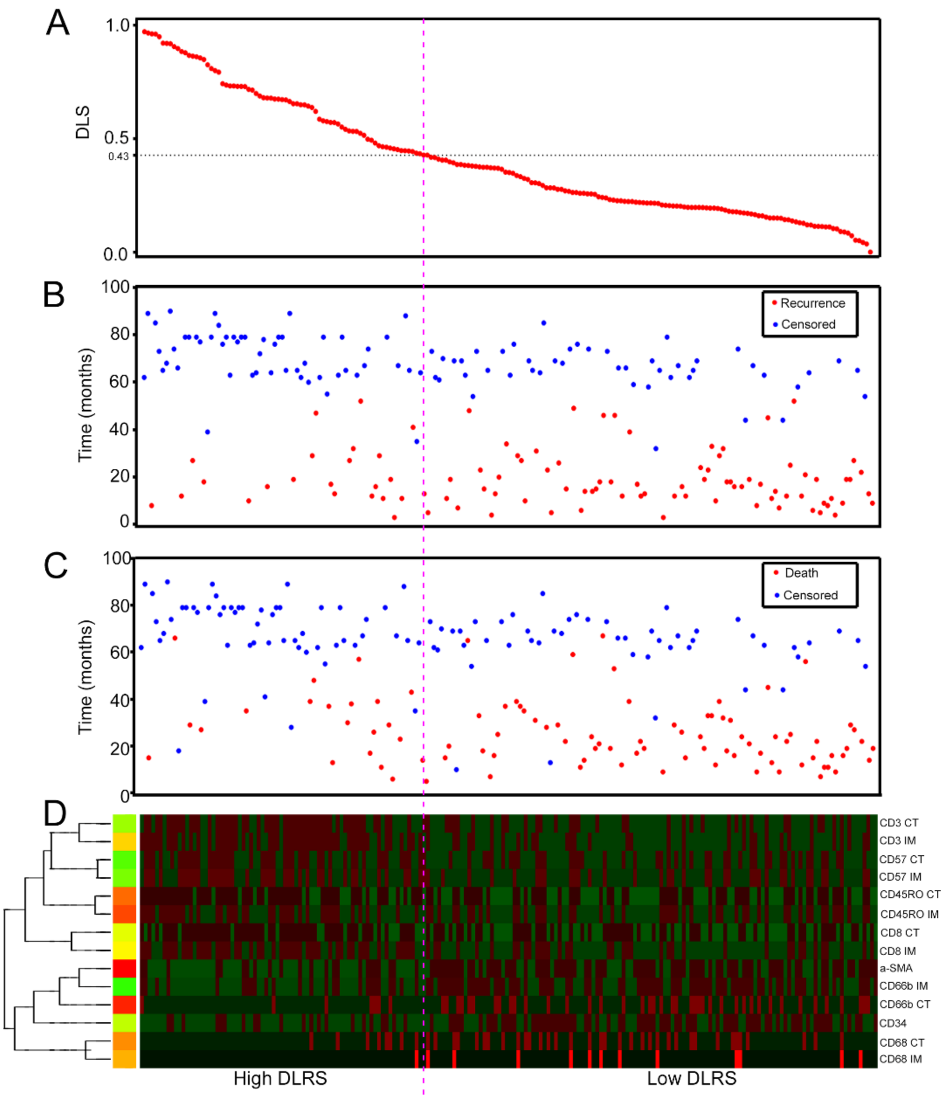

Figure S14. The deep learning score analysis of GC patients in the internal validation cohort 1 (n = 196). Related to Figure 3.

(A) DLRS score of GC patients. (B) Recurrence status of GC patients. (C) Survival status of GC patients. (D) Color-gram of the expression profiles of 14 tumor microenvironment features in GC patients. Rows represent 14 features and columns represent patients. Magenta dotted line represents the DLRS cutoff dividing the patients into high and low DLRS groups.

External validation cohort 1

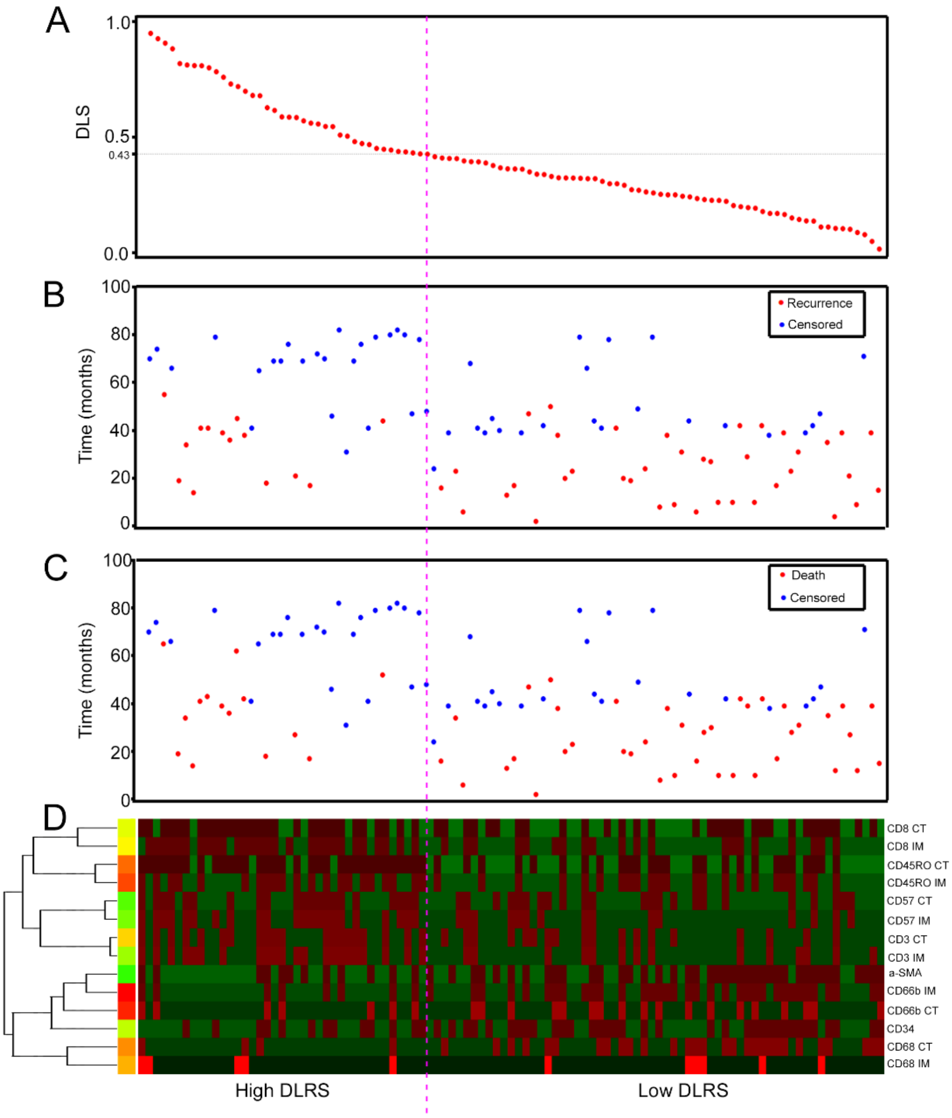

Figure S15. The deep learning score analysis of GC patients in the external validation cohort 1 (n = 101). Related to Figure 3.

(A) DLRS score of GC patients. (B) Recurrence status of GC patients. (C) Survival status of GC patients. (D) Color-gram of the expression profiles of 14 tumor microenvironment features in GC patients. Rows represent 14 features and columns represent patients. Magenta dotted line represents the DLRS cutoff dividing the patients into high and low DLRS groups.

A Disease-free survival

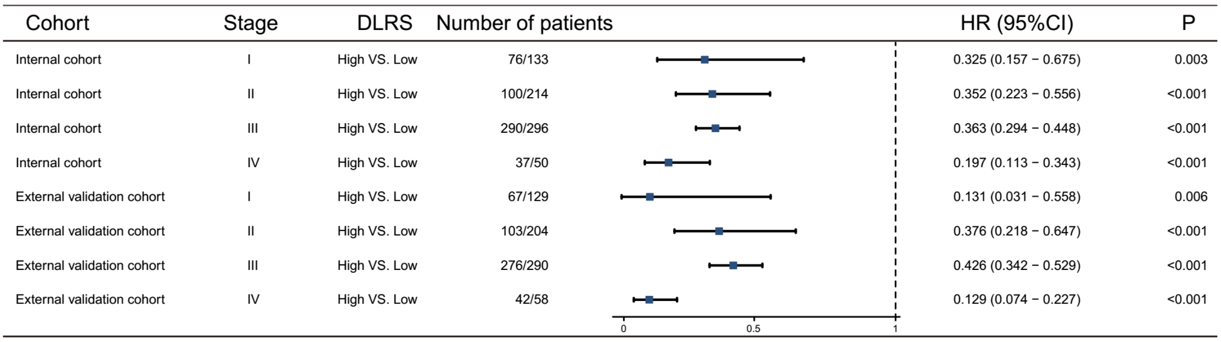

B Overall survival

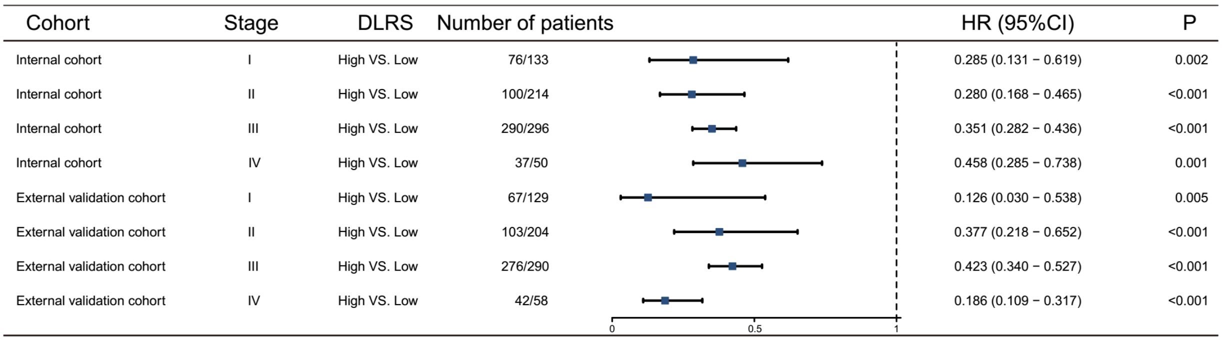

Figure S16. Forest plots summarize the analysis of disease-free survival and overall survival according to the DLRS signature in subgroups of GC patients in the internal cohort (combined training and internal validation cohorts) and external validation cohort. Related to Figure 3. Internal cohort (SMU): Stage I (n = 209), Stage II (n = 314), Stage III (n = 586), Stage IV (n = 87). External cohort (SYSUCC): Stage I (n = 196), Stage II (n = 307), Stage III (n = 566), Stage IV (n = 100).

Disease-free survival

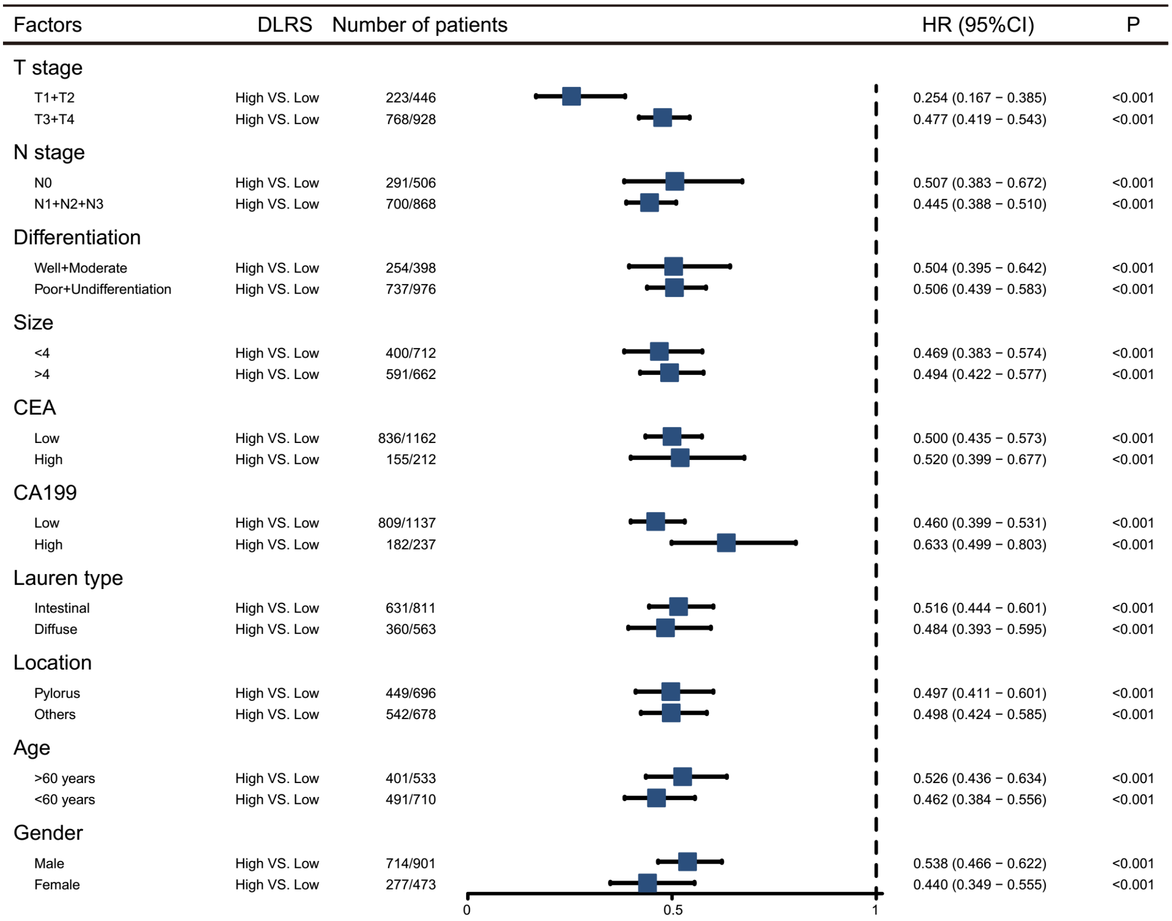

Figure S17. Forest plots summarize the analysis of disease-free survival for all the patients according to the predicted survival score stratified by clinicopathological risk factors. Related to Figure 3.

Overall survival

Factors	DLRS	Number of patients	HR (95%CI)	P
T stage				
T1+T2	High VS. Low	223/446	0.202 (0.128 − 0.320)	<0.001
T3+T4	High VS. Low	768/928	0.470 (0.412 − 0.537)	<0.001
N stage				
N0	High VS. Low	291/506	0.479 (0.358 − 0.639)	<0.001
N1+N2+N3	High VS. Low	700/868	0.427 (0.371 − 0.491)	<0.001
Differentiation				
Well+Moderate	High VS. Low	254/398	0.469 (0.364 − 0.603)	<0.001
Poor+Undifferentiation	High VS. Low	737/976	0.486 (0.420 − 0.561)	<0.001
Size				
<4	High VS. Low	400/712	0.441 (0.358 − 0.543)	<0.001
>4	High VS. Low	591/662	0.476 (0.406 − 0.558)	<0.001
CEA				
Low	High VS. Low	836/1162	0.500 (0.435 − 0.573)	<0.001
High	High VS. Low	155/212	0.520 (0.399 − 0.677)	<0.001
CA199				
Low	High VS. Low	809/1137	0.426 (0.367 − 0.493)	<0.001
High	High VS. Low	182/237	0.672 (0.528 − 0.854)	0.001
Lauren type				
Intestinal	High VS. Low	631/811	0.495 (0.424 − 0.578)	<0.001
Diffuse	High VS. Low	360/563	0.455 (0.368 − 0.564)	<0.001
Location				
Pylorus	High VS. Low	449/696	0.464 (0.382 − 0.564)	<0.001
Others	High VS. Low	542/678	0.482 (0.482 − 0.568)	<0.001
Age				
>60 years	High VS. Low	401/533	0.507 (0.418 − 0.614)	<0.001
<60 years	High VS. Low	491/710	0.440 (0.365 − 0.532)	<0.001
Gender				
Male	High VS. Low	714/901	0.519 (0.447 − 0.602)	<0.001
Female	High VS. Low	277/473	0.407 (0.320 − 0.517)	<0.001

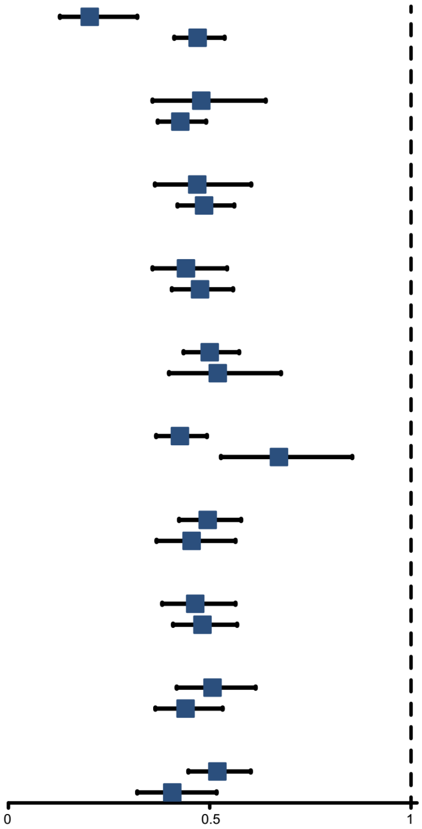

Figure S18. Forest plots summarize the analysis of overall survival for all the patients according to the predicted survival score stratified by clinicopathological risk factors. Related to Figure 3.

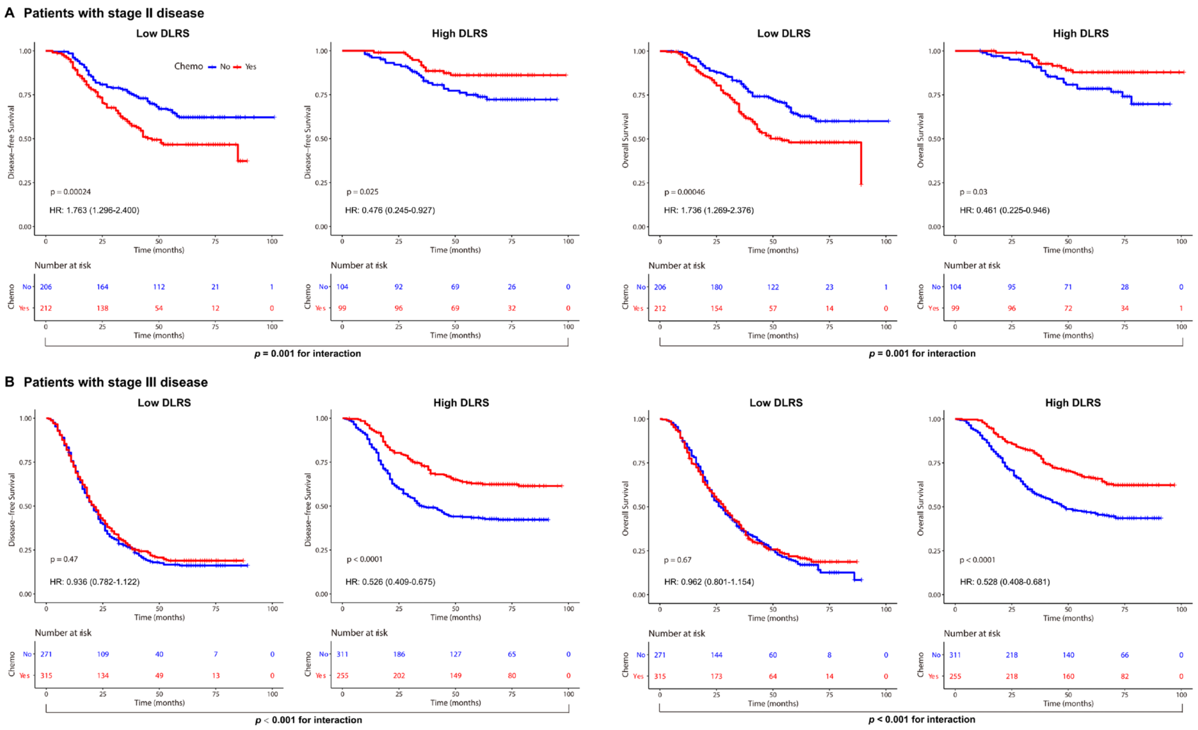

Figure S19. Relationship between the CT imaging-defined DLRS group and disease-free survival and overall survival in unmatched patients who were treated with or without adjuvant chemotherapy. Related to Figure 4 and 5.

A, stage II (n = 621). B, stage III (n = 1,152). Patients were stratified by the receipt of adjuvant chemotherapy. Statistical interaction tests were performed for the following: (A) predicted DLRS classes Low vs. High and adjuvant chemotherapy: $P_{interaction}$ = 0.001 and 0.001 for disease-free survival and overall survival in stage II patients; (2) predicted DLRS classes Low vs High and adjuvant chemotherapy: $P_{interaction}$ <0.001 and <0.001 for disease-free survival and overall survival in stage III patients.

A

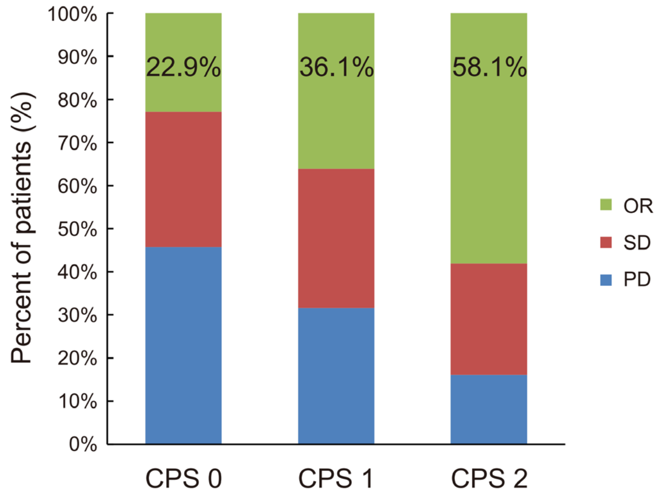

B

Multivariate logistic regression analysis for objective response in patients with different CPS.

Subgroup	Number of patients	OR (95% CI)	P
**CPS High**			
**DLRS High VS. Low**	62/31	2.703 (1.114 − 6.559)	0.028
**CPS Moderate**			
**DLRS High VS. Low**	84/74	17.515 (6.824 − 44.955)	<0.001
**CPS Low**			
**DLRS High VS. Low**	41/29	99999 (0.000 − 999999)	0.998

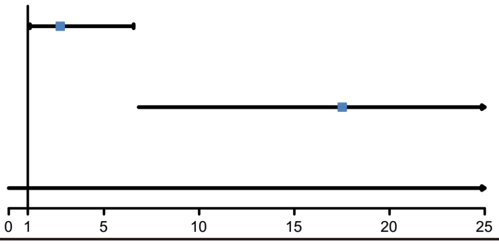

Figure S20. Proportion of response to anti-PD-1 immunotherapy response in different CPS (A), and multivariate logistic regression analysis for objective response in patients with different CPS (B). Related to Figure 6.

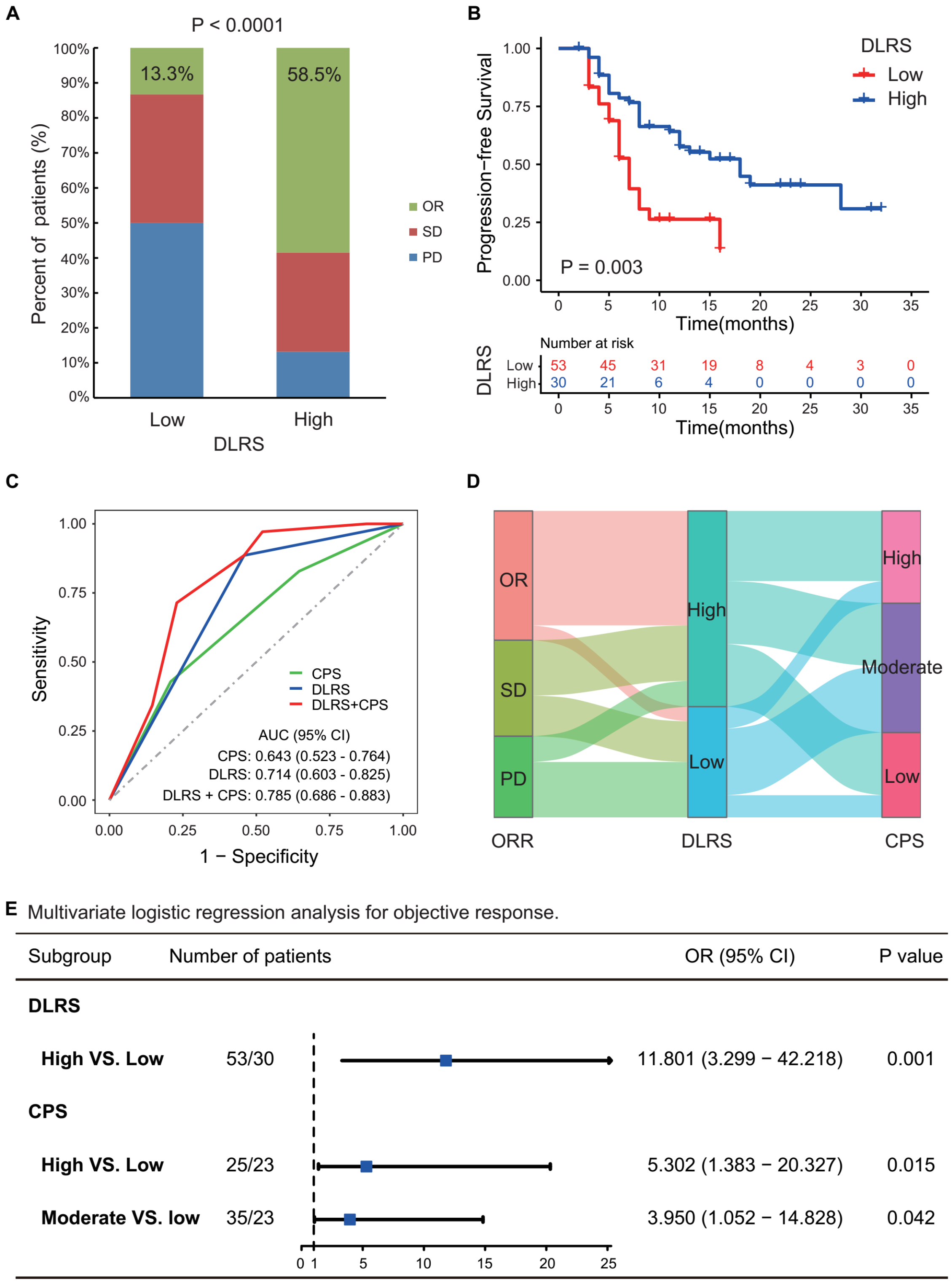

Figure S21. Relationship between the imaging based TME classifier and clinical response and outcomes in patients treated with anti-PD-1 immunotherapy. Related to Figure 6.

(A): Response rates in patients of the DLRS high vs low groups; (B): Progression-free survival in patients of the DLRS high vs low groups; (C), Receiver operator characteristic (ROC) curves of the predicted TME classes, CPS and composite models combining TME classes and CPS for predicting

immunotherapy response (n=83); AUC: DLRS vs. CPS, *P*=0.362; DLRS+CPS vs. CPS, *P*=0.01; DLRS+CPS vs. DLRS, *P*=0.033; (D), Alluvial diagram of the correspondence among patients classified according to the immunotherapy response, DLRS, and CPS in the merged immunotherapy cohorts (n = 83); (E), Forest plot for the multivariate logistic regression analysis for objective response; AUC: area under the receiver operator characteristic curve. CPS: combined positive score of PDL1 expression. OR: objective response (complete and partial response); SD: stable disease; PD: progressive disease.

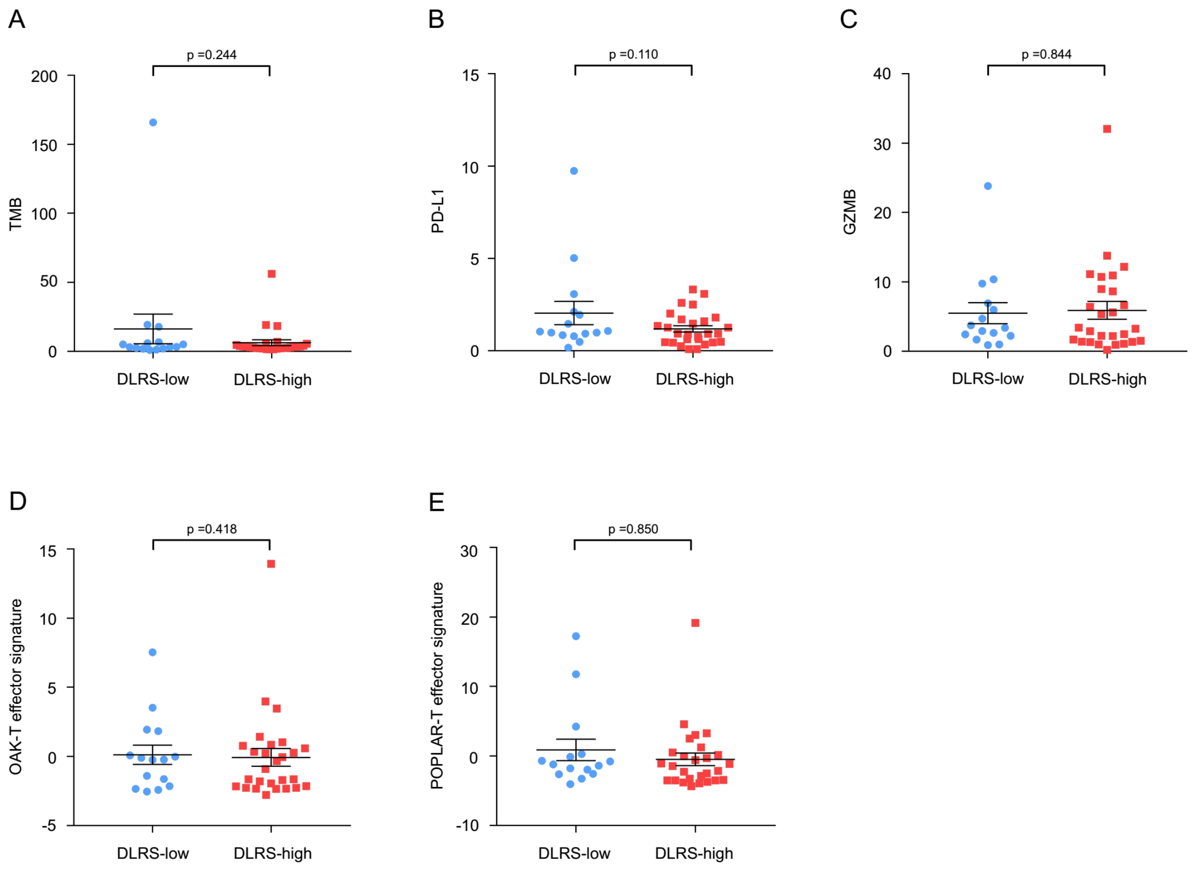

Figure S22. Relationship between the proposed imaging signature DLRS and established genomic and transcriptomic biomarkers, including TMB (A), PD-L1 (B), GZMB (C), OAK-T effector signature (D), and POPLAR-T effector signature (E). Related to Figure 7.
