# Supplementary material for: Non-invasive tumor microenvironment evaluation and treatment response prediction in gastric cancer using deep learning radiomics
Source: Cell Rep Med. 2023 Aug 8;4(8):101146. doi: 10.1016/j.xcrm.2023.101146 (PMC10439253; doi:10.1016/j.xcrm.2023.101146)
Supplement: Document S2. Article plus supplemental information [file mmc10.pdf]

Cell Reports Medicine

Article

Non-invasive tumor microenvironment evaluation and treatment response prediction in gastric cancer using deep learning radiomics

Graphical abstract

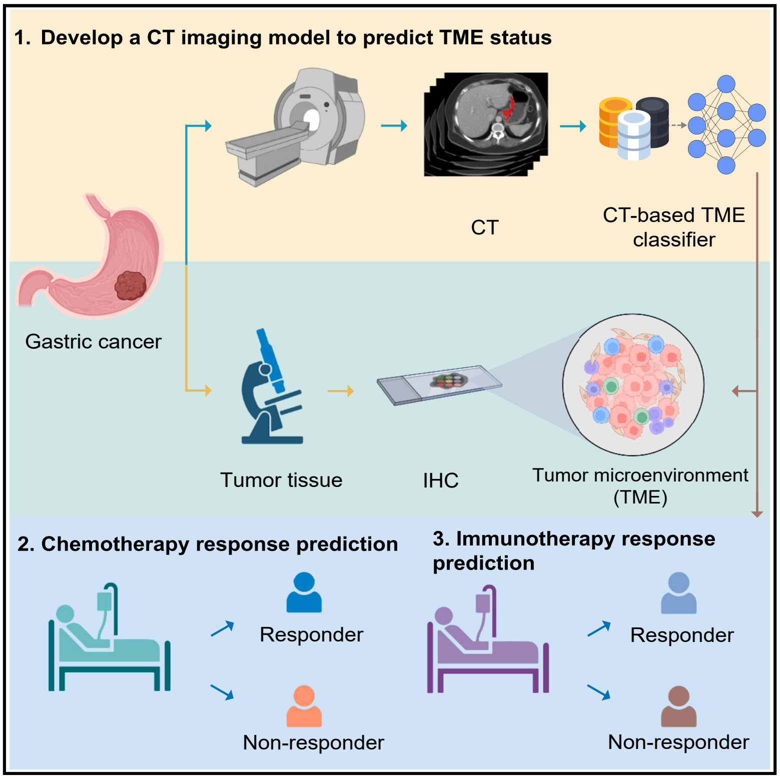

Highlights

- Deep learning radiomics enable noninvasive evaluation of tumor microenvironment
- Imaging signature predicts prognosis and chemotherapy benefit in gastric cancer
- Imaging signature predicts clinical response to immune checkpoint inhibitors

Authors

Yuming Jiang, Kangneng Zhou, Zepang Sun, ..., Wei Wang, Guoxin Li, Ruijiang Li

Correspondence

wangwei@sysucc.org.cn (W.W.), gzliguoxin@163.com (G.L.), rli2@stanford.edu (R.L.)

In brief

Jiang et al. develop an imaging signature by combined deep learning and radiomic analyses that allows noninvasive evaluation of the tumor microenvironment in gastric cancer. The imaging signature predicts the benefit from adjuvant chemotherapy as well as from clinical response to immunotherapy, demonstrating its potential for personalizing treatment of gastric cancer.

Jiang et al., 2023, Cell Reports Medicine *4*, 101146
August 15, 2023 © 2023 The Author(s).
https://doi.org/10.1016/j.xcrm.2023.101146

CellPress

Cell Reports Medicine

CellPress
OPEN ACCESS

Article

Non-invasive tumor microenvironment evaluation and treatment response prediction in gastric cancer using deep learning radiomics

Yuming Jiang,[1,2,9] Kangneng Zhou,[3,9] Zepang Sun,[1,9] Hongyu Wang,[2,9] Jingjing Xie,[4] Taojun Zhang,[1] Shengtian Sang,[2] Md Tauhidul Islam,[2] Jen-Yeu Wang,[2] Chuanli Chen,[5] Qingyu Yuan,[5] Sujuan Xi,[6] Tuanjie Li,[1] Yikai Xu,[5] Wenjun Xiong,[7] Wei Wang,[8,*] Guoxin Li,[1,*] and Ruijiang Li[2,10,*]

[1]Department of General Surgery & Guangdong Provincial Key Laboratory of Precision Medicine for Gastrointestinal Tumor, Nanfang Hospital, Southern Medical University, Guangzhou, China

[2]Department of Radiation Oncology, Stanford University School of Medicine, Stanford, CA, USA

[3]School of Computer and Communication Engineering, University of Science and Technology Beijing, Beijing 100083, China

[4]Graduate Group of Epidemiology, University of California Davis, Davis, CA, USA

[5]Department of Medical Imaging Center, Nanfang Hospital, Southern Medical University, Guangzhou, China

[6]The Reproductive Medical Center, The Seventh Affiliated Hospital of Sun Yat-sen University, Shenzhen, China

[7]Department of Gastrointestinal Surgery, Guangdong Provincial Hospital of Chinese Medicine, The Second Affiliated Hospital of Guangzhou University of Chinese Medicine, Guangzhou, China

[8]Department of Gastric Surgery, and State Key Laboratory of Oncology in South China, Collaborative Innovation Center for Cancer Medicine, Sun Yat-sen University Cancer Center, Guangzhou, China

[9]These authors contributed equally

[10]Lead contact

*Correspondence: wangwei@sysucc.org.cn (W.W.), gzliguoxin@163.com (G.L.), rli2@stanford.edu (R.L.)

https://doi.org/10.1016/j.xcrm.2023.101146

SUMMARY

The tumor microenvironment (TME) plays a critical role in disease progression and is a key determinant of therapeutic response in cancer patients. Here, we propose a noninvasive approach to predict the TME status from radiological images by combining radiomics and deep learning analyses. Using multi-institution cohorts of 2,686 patients with gastric cancer, we show that the radiological model accurately predicted the TME status and is an independent prognostic factor beyond clinicopathologic variables. The model further predicts the benefit from adjuvant chemotherapy for patients with localized disease. In patients treated with checkpoint blockade immunotherapy, the model predicts clinical response and further improves predictive accuracy when combined with existing biomarkers. Our approach enables noninvasive assessment of the TME, which opens the door for longitudinal monitoring and tracking response to cancer therapy. Given the routine use of radiologic imaging in oncology, our approach can be extended to many other solid tumor types.

INTRODUCTION

Gastric cancer (GC) is a highly prevalent malignancy and is the leading cause of cancer-related deaths worldwide.[1] Currently, the most important factor in risk stratification and treatment decisions is the TNM staging system.[2] However, large variations in treatment response and outcomes are observed for patients with disease of identical stage, suggesting that the current prognostic model could not provide complete predictive information.[2,3] Therefore, an improved stratification of GC is needed to more accurately predict patient prognosis and treatment response.

Extensive research on the tumor microenvironment (TME) has shed new light on the molecularly based classification of cancers.[3–8] Based on the quantification of various cell subpopulations in the TME, several biomarkers have been shown to be associated with prognosis and with response to chemotherapy and immunotherapy such as immune checkpoint inhibitors.[3,7] In GC, patients whose tumors are infiltrated by cytotoxic $CD8^+$ T lymphocytes have a prolonged survival, while those with a high amount of neutrophils have a poor prognosis.[3,7,9]

The gold standard for TME evaluation is based on histopathology. However, this approach suffers from the fundamental limitation of sampling bias due to intratumor heterogeneity[10] and is also limited by insufficient tumor tissue available in practice. Therefore, a noninvasive means to assess the TME would be valuable, especially in the neoadjuvant therapy setting or metastatic disease.

Radiological imaging allows visualization of the entire tumor and is routinely used for diagnosis, staging, evaluation for treatment response, and follow up of patients with cancer. Sophisticated imaging analysis can reveal the link between subtle radiological phenotypes and specific aspects of the underlying pathobiology including the TME.[11] Two broad approaches have been explored: (1) radiomics analysis with hand-crafted

© 2023 The Author(s).
This is an open access article under the CC BY license (http://creativecommons.org/licenses/by/4.0/).

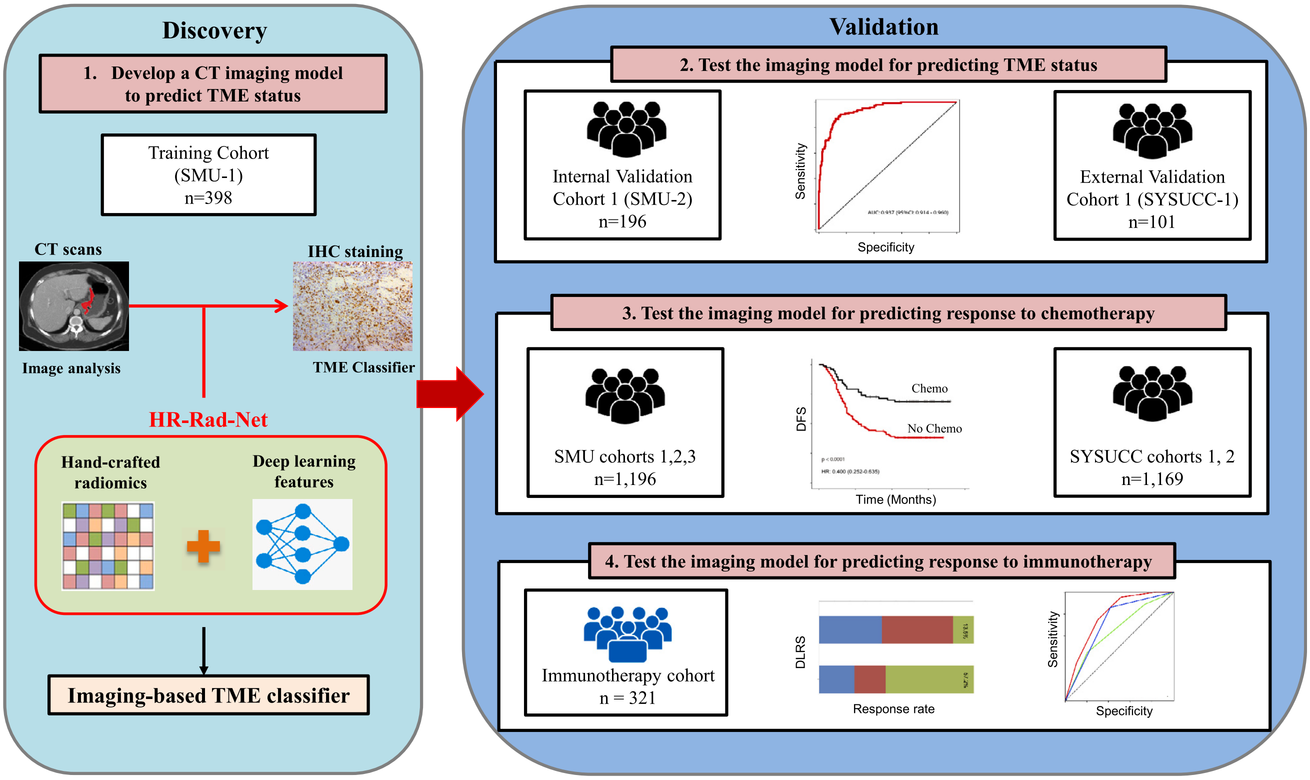

Figure 1. Study design for the discovery and validation of a deep learning model based on CT images to assess tumor microenvironment and treatment outcomes in gastric cancer

Both CT images and IHC stains were available for patients in the SMU-1 (training) cohort and the SMU-2 and SYSUCC-1 (internal and external validation) cohorts, which were used for testing the model's accuracy for predicting tumor microenvironment status. All patients had CT and treatment outcomes available, which were used for testing the prognostic and predictive value of the model. CT, computed tomography; IHC, immunohistochemistry; SMU, Southern Medical University; SYSUCC, Sun Yat-sen University Cancer Center.

image features[12–14] and (2) deep learning, which can automatically learn feature representations from images.[15–20] While most applications are focused on clinical diagnosis, emerging studies have shown the feasibility of using deep learning to predict biological features from medical images.[21]

We previously developed and validated a machine learning classifier of the overall TME status based on immunohistochemistry assessment of eight immune and stroma features, which predicted survival and benefit from adjuvant chemotherapy in GC.[7] In this study, we aim to develop a noninvasive imaging-based model of the TME classifier by combining deep learning and radiomics analysis. We will further validate the model for predicting prognosis and response to chemotherapy and immunotherapy.

RESULTS

Patient characteristics

Table S1 lists the clinicopathological characteristics of the patients in the training (n = 398), internal validation 1 (n = 196), internal validation 2 (n = 602), external validation 1 (n = 101), and external validation 2 (n = 1,068) cohorts. All these patients (n = 2,365) had resection for GC (Figure S1). Among them, 1,615 (68.3%) were men, and the median age was 57 (interquartile range: 48.5–64) years. The majority of patients (n = 1,773, 75%) had stage II or III disease, among whom 881 (49.7%) patients received adjuvant chemotherapy. The clinicopathological data of the immunotherapy cohort are shown in Table S2.

Deep learning radiomics model predicts TME classifier from CT images

We trained a deep learning radiomics model to predict the TME classifier from computed tomography (CT) images (Figures 1 and S2). The model combines a deep convolutional neural network with hand-crafted features to derive an imaging-based TME classifier, i.e., deep learning radiomics signature (DLRS). The implementation of the deep learning radiomics model is available at https://github.com/MontaEllis/HR-Rad-Net. Figures S3 shows some representative cases with CT images and visualization of the network prediction. The proposed model had an area under the curve (AUC) of 0.937 (95% confidence interval [CI], 0.914–0.960) for predicting the TME classifier in the training cohort (Figure 2A). Similarly, the model achieved high levels of discriminability with AUCs of 0.912 and 0.909 in the internal and external validation cohorts, respectively (Figure 2A). As expected, the model's output DLRS was significantly higher in the TME-high group than that in the TME-low group in all three cohorts (Figure 2B). Additional performance metrics including the overall

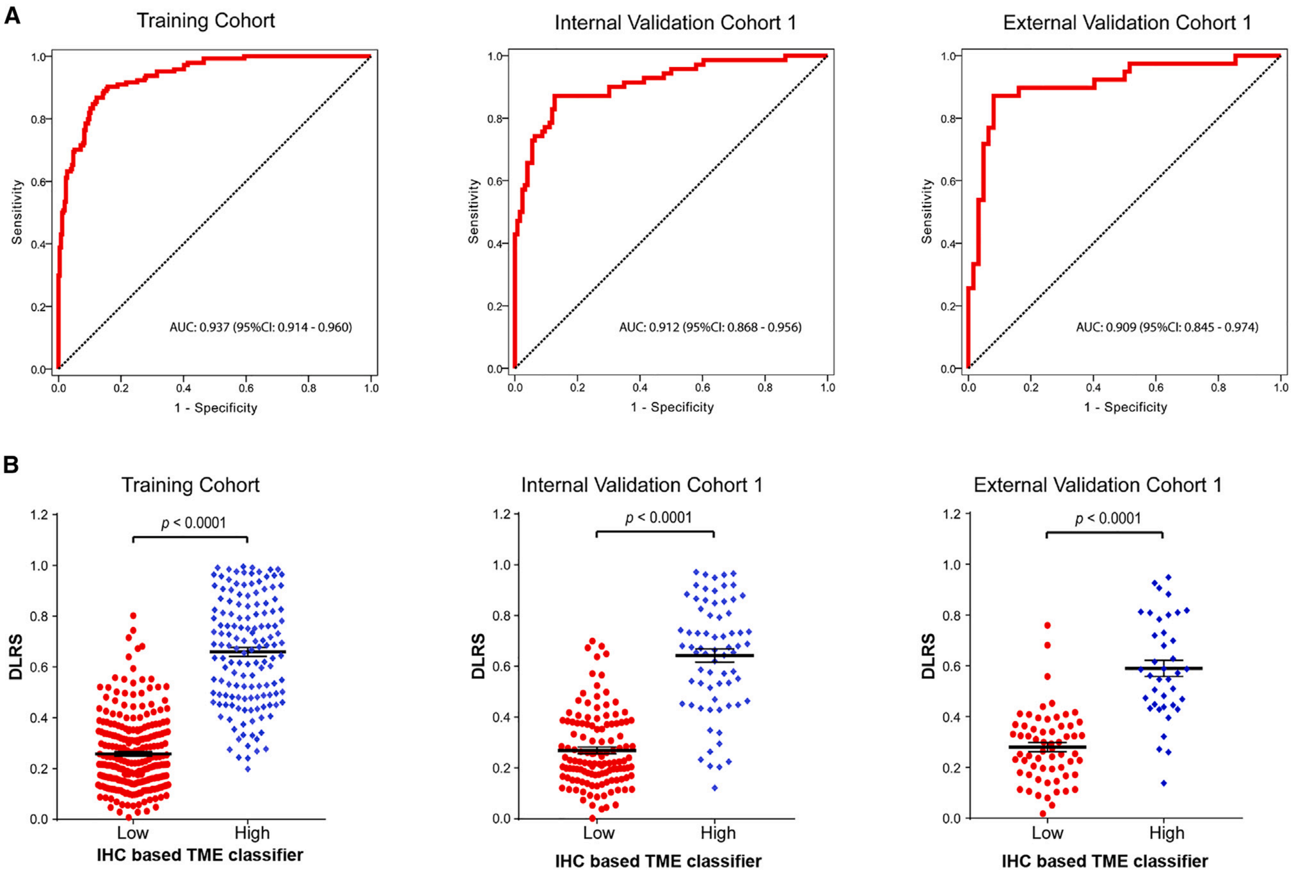

C

Performance of the deep learning signature in the training and validation cohorts

DLRS	AUC(95% CI)	Accuracy(95% CI)	Sensitivity(95% CI)	Specificity(95% CI)
**Training cohort**	0.937 (0.914 - 0.960)	0.874 (0.842 - 0.907)	98.46 (94.6 - 99.8)	70.26 (65.4 - 74.8)
**Internal Validation Cohort 1**	0.912 (0.868 - 0.956)	0.872 (0.826 - 0.919)	88.08 (82.7 - 92.3)	69.16 (65.2 - 72.9)
**External Validation Cohort 1**	0.909 (0.845 - 0.974)	0.891 (0.830 - 0.952)	98.89 (96.8 - 99.8)	52.13 (48.5 - 55.7)

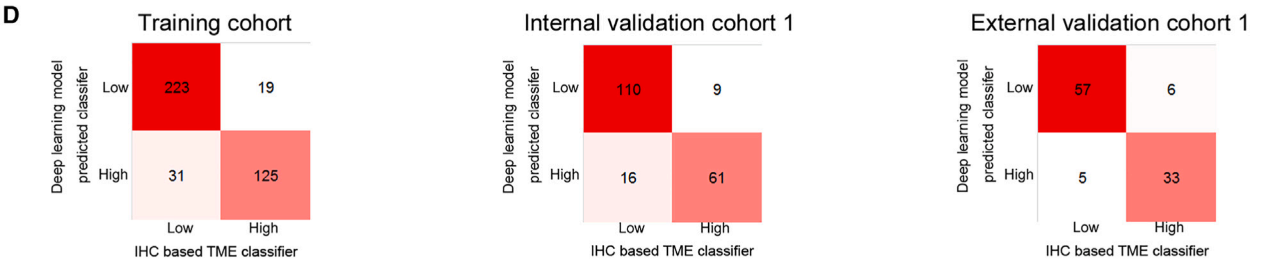

Figure 2. Performance of the deep learning model to assess tumor microenvironment in the training cohort, internal validation cohort 1, and external validation cohort 1

(A) Receiver operator characteristic (ROC) curves.

(B) Distributions of DLRS by IHC-defined TME classifier.

(C) Performance of the image signature in the training and validation cohorts.

(D) Confusion matrices in the training and validation cohorts. The confusion matrices show the pairwise comparison; diagonal: number cases of correctly classified; off-diagonal: number cases of in correctly classified. AUC, area under the curves; TME, tumor microenvironment; DLRS: deep learning radiomics score.

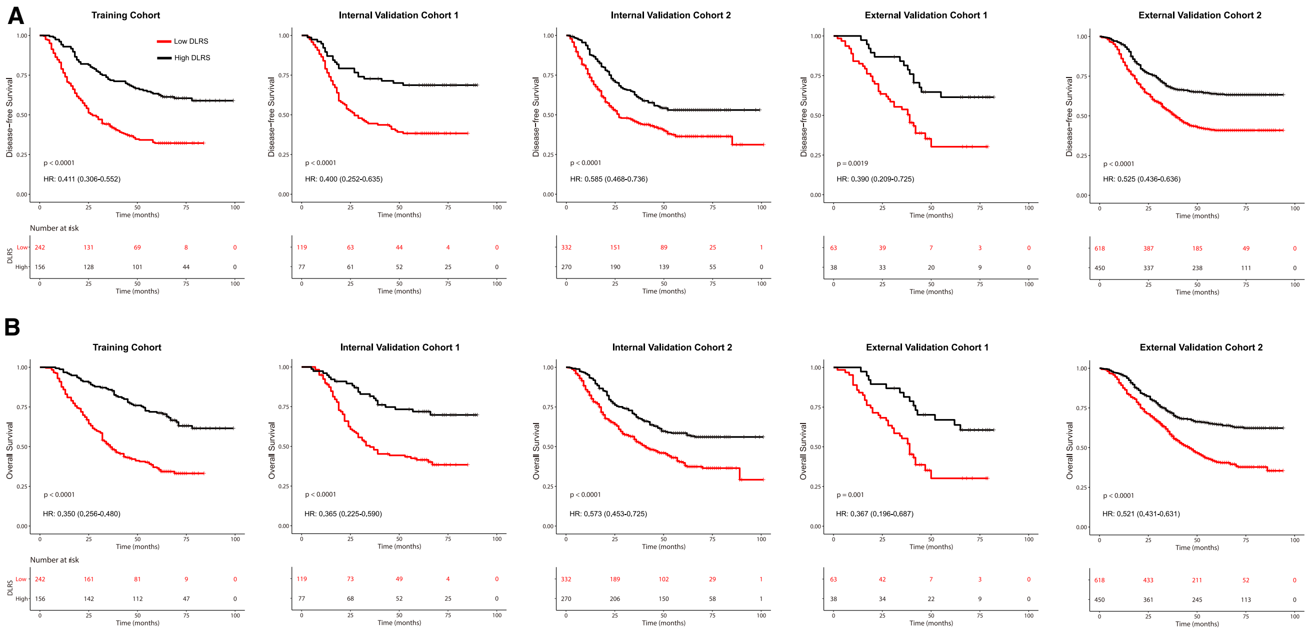

Figure 3. Kaplan-Meier analyses of disease-free survival (DFS) and overall survival (OS) according to the DLRS in patients with gastric cancer

(A) Disease-free survival.

(B) Overall survival. Training cohort (n = 398), internal validation cohort 1 (n = 196), internal validation cohort 2 (n = 602), external validation cohort 1 (n = 101), and external validation cohort 1 (n = 1,068).

accuracy, sensitivity, and specificity are shown in Figure 2C. Consistently, the confusion matrix showed that the model predictions agreed well with the TME classifier defined by immunohistochemistry (IHC) (Figure 2D). The calibration curve also showed excellent agreement between the predicted probabilities for the TME classifier and observations in all cohorts (Figure S4A). The decision curve analysis showed a high net benefit for the model across a range of relevant threshold probabilities (Figure S4B).

Based on the optimum cutoff determined by the receiver operating characteristic (ROC) curve analysis in the training cohort (Figure 2A), patients were divided into a DLRS-low group (DLRSs < 0.428) and a DLRS-high group (DLRSs $\geq$ 0.428). The relationships between the DLRS and clinicopathological characteristics are summarized in Table S3.

Additionally, we compared performance of the proposed HR-Rad-Net model with alternative deep-learning approaches for predicting the TME classifier. In both validation cohorts, our model improved the prediction accuracy compared with the model trained without including radiomics features as well as the original HR-Net model trained without incorporating the squeeze and excitation strategy (Figure S5).

DLRS is correlated with immune cell infiltration and stroma abundance

We assessed the relationship between DLRS and individual TME features in 695 patients for whom IHC data were available (i.e., by merging patients in the training cohort, internal validation cohort 1, and external validation cohort 1). This analysis revealed two distinct clusters: a dominant cluster of 9 features for DLRSs and lymphocytes (CD3TC, CD3IM, DLRS, CD8TC, CD8IM, CD45ROTC, CD45ROIM, CD57TC, and CD57IM) and a second cluster of 6 features for fibroblast, microvessel, and myeloid cells (a-SMA, CD34, CD66bTC, CD66bIM, CD68TC, and CD68IM) (Figure S6A). A positive correlation was observed between the DLRS and lymphocyte features (all $p < 0.05$), and a negative correlation was observed between the DLRS and features of fibroblast, vessel, and myeloid cells (Figures S6–S12). We also assessed the distribution of DLRSs, recurrence and survival status, and the expression of the 14 TME features. Tumors with high DLRSs generally exhibited increased expression of CD3IM, CD3TC, CD8IM, and CD45ROTC and reduced expression of fibroblast, microvessel, and myeloid cells. High-DLRS patients had fewer recurrences and deaths than low-DLRS patients did (Figures S13–S15).

DLRS is associated with prognosis

We assessed the prognostic value of the DLRS. In the training cohort, the 5-year disease-free survival (DFS) and overall survival (OS) were 21.1% and 28.5% for the low-DLRS group; by contrast, the 5-year DFS and OS were 48.9% and 54.0% for the high-DLRS group (Figure 3). Similarly, in the internal validation cohort 1, the 5-year DFS and OS were 21.9% and 24.8% for the low-DLRS group compared with 48.7% and 55.5% for the high-DLRS group (Figure 3). We further confirmed significantly different prognoses for patients stratified by the DLRS in all internal and external validation cohorts (Figure 3).

We performed multivariate Cox regression analysis adjusting for clinicopathological variables. The DLRS remained an independent prognostic factor for predicting DFS and OS in the

CellPress
OPEN ACCESS

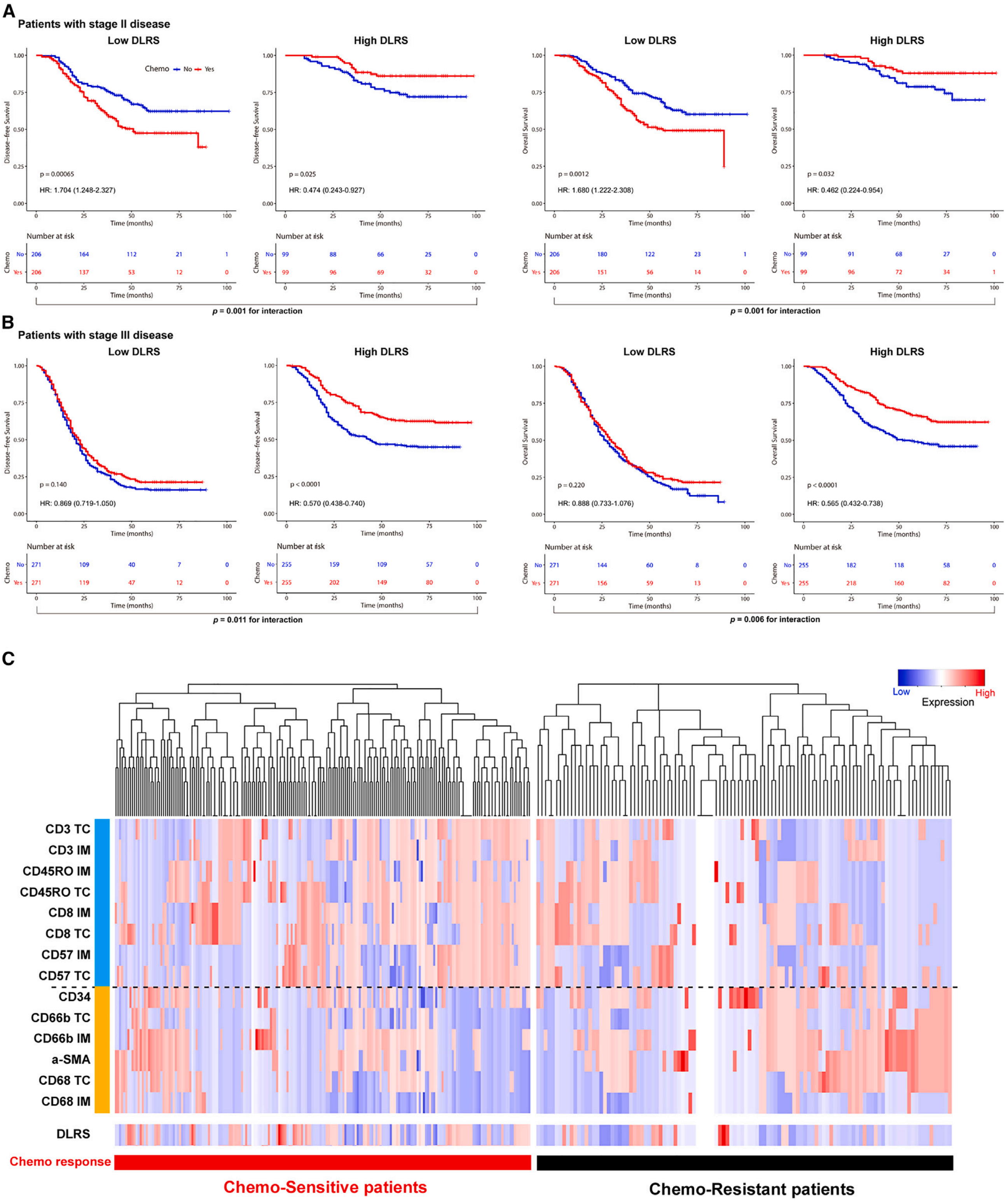

(legend on next page)

training and validation cohorts (Tables S4 and S5). We performed additional analyses within subgroups of patients stratified by various clinicopathological risk factors. Of note, patients with high DLRS had improved DFS and OS compared with patients with low DLRSs within each stage I, II, III, or IV (Figure S16). In addition, when stratified by other factors such as tumor size, location, histology, and differentiation, the DLRS remained a statistically significant prognostic factor in these subgroups (Figures S17 and S18). These data show that the DLRS is a strong independent prognostic factor in GC.

DLRS predicts benefit from adjuvant chemotherapy

We further evaluated predictive value of the DLRS regarding the benefit of adjuvant chemotherapy after surgery in stage II and III patients. To do this, we first performed propensity score matching to ensure that the characteristics of patients who received chemotherapy were similar to those who did not (Tables S6). We then compared the survival for patients who either received or did not receive chemotherapy according to the DLRS.

We found that for patients in the high-DLRS group, adjuvant chemotherapy was associated with an improved prognosis for both stage II and III disease, e.g., for DFS, stage II: hazard ratio (HR) 0.474 (95% CI, 0.243–0.927), $p = 0.025$, and stage III: HR 0.570 (0.438–0.740), $p < 0.001$ (Figures 4A and 4B). On the other hand, for patients in the low-DLRS group, adjuvant chemotherapy was not associated with an improvement in DFS in either stage II or III disease. In fact, for stage II patients, chemotherapy was associated with a significantly worse DFS (HR = 1.704 [95% CI, 1.248–2.327], $p = 0.007$) in the low-DLRS group. A statistical interaction test was performed between the DLRS signature and chemotherapy, which confirmed a significant interaction effect ($p < 0.05$) regarding the impact on DFS and OS. Additionally, we performed the above analyses using all the patients without propensity score matching and obtained similar results (Figure S19). We also performed multivariate logistic regression analysis and found that DLRS is an independent factor for predicting chemotherapy sensitivity in GC (Table S7). These data suggest that the DLRS may be predictive of the benefit from adjuvant chemotherapy in stage II and III disease.

For the stage II and III patients treated with chemotherapy (n = 881), we divided them into two groups, chemo-sensitive vs. chemo-resistant, based on if they derived or did not derive a survival benefit from chemotherapy (DFS $\geq$ or <2 years). Chemo-sensitive patients had significantly higher DLRS scores than chemo-resistant patients in the training and validation cohorts (Figure 5A). Consistently, patients with high DLRS scores had a significantly higher probability of being chemo-sensitive than those with low DLRS scores, and vice versa (Figure 5B). We further explored the association between chemotherapy responsiveness and individual TME features (Figures 4C and 5C). Chemo-sensitive GC had a higher expression of lymphocytes such as CD3IM, CD3TC, CD8IM, and CD45ROTC (Figure 5C). On the other hand, chemo-resistant GC had a higher expression of fibroblasts and neutrophils (a-SMA, CD66bTC, and CD66bIM) (Figure 5C).

DLRS predicts response to anti-PD-1 immunotherapy

We finally investigated relationships between the DLRS and the response to anti-PD-1 immunotherapy in a cohort of 321 patients with advanced GC. The overall objective response rate was 39.6%. Patients in the high-DLRS group achieved a substantially higher objective response rate (57.8%) compared with those in the low-DLRS group (14.2%) (Figure 6A). For all patients, the median progression-free survival (PFS) was 10 months. Kaplan-Meier analysis showed that the DLRSs were significantly associated with PFS ($p < 0.001$; Figure 6B). The median PFSs were 18 and 7 months in patients in the high- and low-DLRS groups, respectively.

Although the combined positive score (CPS) of PD-L1 expression, a clinically approved biomarker of immunotherapy response, was also associated with objective response (Figure S20A), the predictive accuracy was quite modest, with an AUC of 0.642 (95% CI, 0.580–0.703) (Figures 6C and 6D). In comparison, the DLRSs showed a higher accuracy for predicting objective response (AUC: 0.722 [0.665–0.778]; Figure 6C). In multivariate regression analysis, DLRSs had a stronger effect on objective response than CPS (Figure 6E). Importantly, DLRSs can further distinguish patients with differential response within the CPS-moderate and CPS-high subgroups (Figure S20B), suggesting complementary value between the two. Therefore, we combined CPS and DLRS into an integrative model (Figure 6C), which significantly improved the accuracy for immunotherapy response prediction (AUC: 0.783 [0.734–0.833], $p < 0.0001$) compared with CPS. Furthermore, we performed the subgroup analysis in 83 patients who were treated with single-agent immunotherapy, and observed similar results for predicting response to immunotherapy (Figure S21).

Molecular correlates of the DLRS

We performed radiogenomics analysis to investigate the biological underpinnings of the DLRS. For this analysis, we leveraged the TCGA/TCIA-STAD dataset, which contains publicly available genomic/transcriptomic data and matched CT images for 42 patients with GC. We processed the CT images to compute DLRSs and performed gene set enrichment analyses to identify the underlying molecular pathways associated with the DLRSs. This analysis showed that tumors in the DLRS-low group were significantly enriched for multiple cancer hallmark-related pathways such as MYC signaling, KRAS signaling, and epithelial mesenchymal transition that are associated with aggressive tumor

Figure 4. Relationship between the DLRS and DFS in matched patients who were treated with or without adjuvant chemotherapy

(A) Stage II (n = 610).

(B) Stage III (n = 1,052). Patients were stratified by the receipt of adjuvant chemotherapy. Statistical interaction tests were performed for the following: (left panel) predicted DLRS low vs. high and adjuvant chemotherapy: $p_{interaction} = 0.001$ and 0.011 for stage II and stage III; (right panel) predicted DLRS low vs. high and adjuvant chemotherapy: $p_{interaction} = 0.001$ and 0.006 for stage II and stage III.

(C) Hierarchical tree structure classifying the stage II and III patients who received chemotherapy according to the levels of DLRS and 14 TME features: high expression (red) and low expression (green).

CellPress
OPEN ACCESS

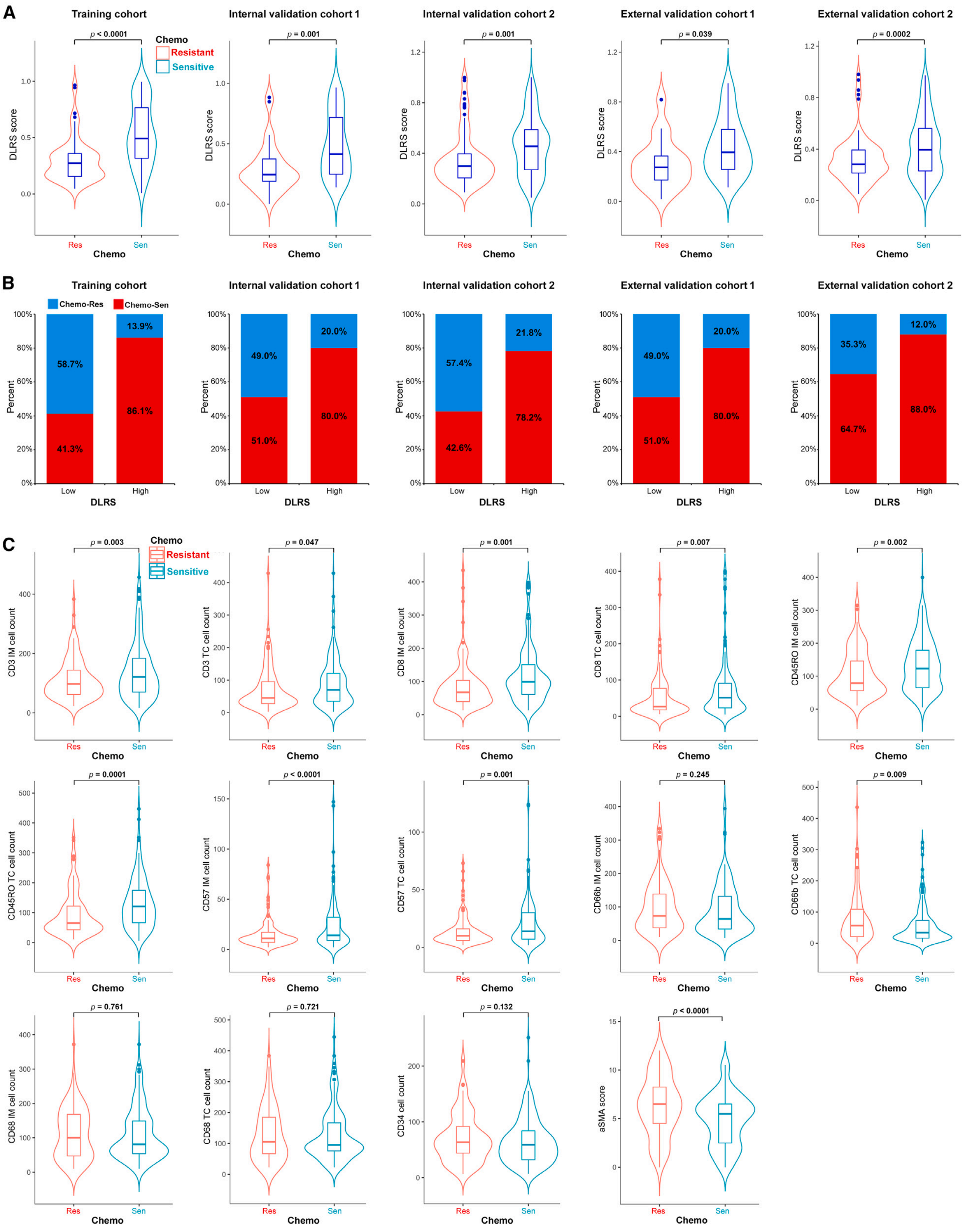

(legend on next page)

phenotypes (Figure 7A). In addition, tumors in the DLRS-low group were also enriched for immune suppression-related pathways such as negative regulation of immune system and effector processes as well as chemokine signaling that may play a role in the migration of myeloid-derived suppressor cells into the tumor (Figures 7A and 7C). Interestingly, we also found several metabolism-related pathways, including glycolysis, oxidative phosphorylation, and fatty acid metabolism pathways, associated with DLRS-low tumors (Figures 7B and 7C). Overall, these findings are consistent with their unfavorable prognosis and response rates and might suggest potential therapeutic targets for overcoming resistance to immunotherapy.

Finally, we assessed the relationship between the DLRSs and established biomarkers of immunotherapy response including TMB, PD-L1, GZMB, and T effector signatures. We did not find any significant associations between DLRSs and these existing biomarkers, suggesting that DLRSs might provide additional information for predicting immunotherapy response (Figure S22).

DISCUSSION

In this study, we developed and validated an imaging signature to noninvasively assess the TME in GC by quantitative analysis of CT images. Further, we demonstrated prognostic value of the imaging signature, which was independent of traditional clinicopathologic risk factors. Importantly, we showed that the imaging signature could identify which patients will benefit from adjuvant chemotherapy as well as improve the prediction of response to immunotherapy.

There is growing evidence for the prognostic and predictive relevance of TME, which has been established as a key determinant of treatment response and outcomes in many cancers. However, major challenges remain for the reliable evaluation of TME. In addition to the need for high-quality tissue, which is often limited in clinical practice, the current histological approach is prone to sample errors due to intratumor spatial heterogeneity and dynamic evolution of the TME.

Radiological imaging provides some unique advantages that may overcome these challenges. Imaging allows noninvasive evaluation and longitudinal monitoring of the entire tumor *in situ*. Because radiological phenotypes are fundamentally driven by the underlying pathophysiology, quantitative imaging analysis may reveal subtle relations between the two.[22] The feasibility of this idea has been demonstrated in previous studies.[12,23–25] Sun et al. developed a radiomic signature of CD8 T cells, which was correlated with clinical response and outcomes of patients treated with anti-PD1 immunotherapy.[12] In our recent work, we developed a deep learning-based imaging signature of tumor stroma, which predicted prognosis and the benefit of adjuvant chemotherapy in GC.

Our study represents a clinical and technical advance over prior work in several aspects. First, previous studies have primarily focused on tumor-infiltrating lymphocytes,[12,26] which only provide a simplified and partial view of the TME. Here, we evaluated both lymphoid/myeloid immune cells as well as stromal and vascular components that better capture the complexity and heterogeneity of the TME across patients. We note that the ground truth for TME status was based on IHC staining of large surgical specimens, which is more representative of the tumor than a small biopsy. Second, in deriving the imaging signature, we combined the respective merits of knowledge-based radiomics and data-driven deep learning approaches and showed superior performance over either approach. Third, we validated the prognostic and predictive values of our model in large multi-institutional cohorts of patients treated with chemotherapy and immunotherapy.

The standard treatment for localized GC includes surgery followed by adjuvant chemotherapy to prevent disease recurrence and improve survival. However, some studies have reported that certain subgroups of patients may not benefit from adjuvant chemotherapy.[2,3,27,28] The optimal criteria for selection of candidates for adjuvant chemotherapy remain controversial. In our study, we found that only stage II/III patients classified as high DLRS were able to derive survival benefit from adjuvant chemotherapy, whereas patients classified as low DLRS did not benefit. In future clinical trials, novel personalized approaches to deintensify or intensify treatment based on the risk profile and TME status for these patients could be tested to enhance the efficacy of systemic therapies.

There is an unmet need for reliable biomarkers to identify which patients will respond to immunotherapy, which has become a standard treatment for many cancer types. We showed that the DLRS could predict response to anti-PD-1 immunotherapy in advanced GC. Specifically, tumors with high immune cell infiltration showed good response to immunotherapy. However, tumors with high stroma and vasculature had poor response. This is consistent with previous findings based on molecular approaches to TME evaluation.[29] The imaging signature of TME had a stronger predictive effect than PD-L1 expression, a clinically approved biomarker of immunotherapy response. Importantly, combining the TME classifier with PD-L1 expression significantly improved the accuracy for response prediction. Although tumors with mismatch repair deficient (dMMR)/microsatellite instability-high (MSI-H) status have a much higher response rate, our model could identify a subset of tumors that do not respond to anti-PD-1 immunotherapy, and combination treatment strategies will be required for these patients.

Rather than studying the relation between radiological imaging and TME, an alternative approach is to apply radiomic or deep-learning analysis to directly predict treatment response or outcome, and many studies have reported promising results in gastric and other cancers.[30–35] However, a critical issue has been the lack of biological interpretation of these imaging signatures. By contrast, our work builds on the extensive evidence for the well-established role of the TME in disease progression and

Figure 5. Relationship between the DLRS and chemotherapy response and TME characteristics
(A) Violin plot showing DLRS scores in stage II and III patients resistant or sensitive to adjuvant chemotherapy.
(B) Rate of clinical response (resistant, sensitive) to adjuvant chemotherapy in high- or low-DLRS score groups.
(C) Violin plot showing TME features in stage II and III patients resistant or sensitive to adjuvant chemotherapy.

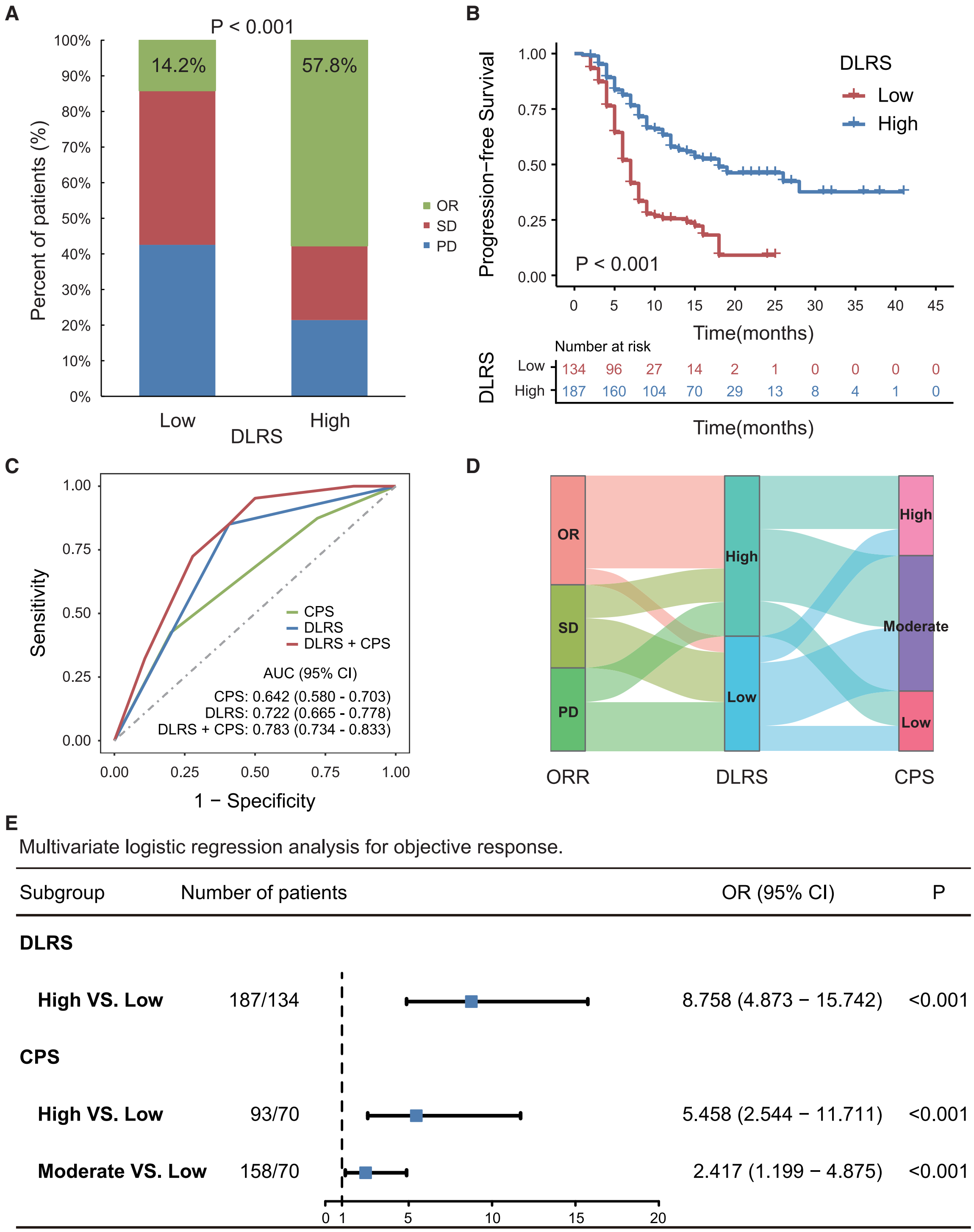

Figure 6. Relationship between the DLRS and clinical response and outcomes in patients treated with anti-PD-1 immunotherapy

(A) Response rates in patients of the DLRS-high vs. -low groups.

(B) Progression-free survival in patients of the DLRS high vs. low groups.

(C) ROC curves of the predicted TME classes, CPS, and composite models combining TME classes and CPS for predicting immunotherapy response ($n = 321$); AUC: DLRS vs. CPS, $p = 0.04$; DLRS+CPS vs. CPS, $p < 0.0001$; DLRS+CPS vs. DLRS, $p < 0.0001$.

(legend continued on next page)

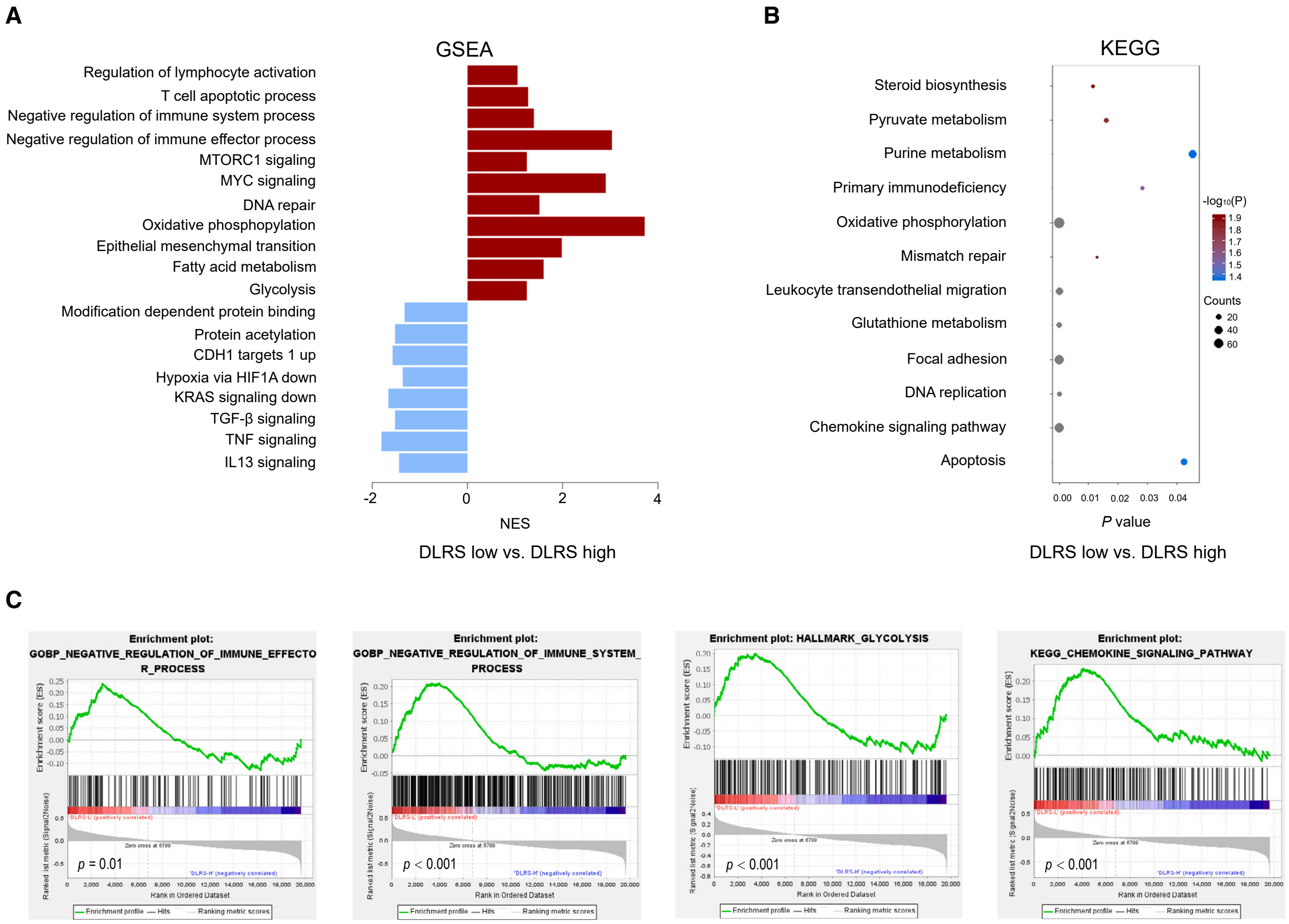

Figure 7. Molecular correlates of the DLRS in gastric cancer

(A) Bar plot shows the top enriched molecular pathways by normalized enrichment score (NES) in the DLRS-high group (blue) and the DLRS-low group (red). A positive NES score indicates the pathway is significantly enriched in the DLRS-low group, and a negative NES indicates the pathway is significantly enriched in the DLRS-high group.

(B) Bubble plot shows the top enriched pathways by gene counts along with p values.

(C) Examples of the enrichment plot for the molecular pathways significantly associated with the DLRS.

its impact on treatment response,[36–39] thus providing the biological rationale behind the model predictions.

Limitations of the study

Our study is retrospective, which makes it susceptible to selection biases. To address this issue, we included large independent cohorts of patients from multiple institutions to validate our findings. Second, the decision about use of chemotherapy was made by the clinicians and/or patients, and thus the predictive effect of our imaging model should be validated in prospective randomized trials. Third, patients were enrolled from one geographic region in China; the distribution of clinical and pathological characteristics might be different in other populations.

Future work will focus on prospective validation of the imaging signature to confirm the generalizability and reproducibility in larger populations and across different scanners. While imaging will not replace tissue-based evaluation for TME, we envision that it could be used as an adjunct tool (like liquid biopsy for tumor genomics) to supplement current histopathology approaches where tumor tissue is unavailable or inadequate.

In conclusion, we developed a noninvasive radiological model for the assessment of TME status using deep learning and radiomics analysis of CT images. The imaging signature has the potential to refine prognosis and guide personalized therapy of GC. Prospective studies and randomized trials are required to confirm its clinical validity and clinical utility.

(D) Alluvial diagram of the correspondence among patients classified according to the immunotherapy response, DLRS, and CPS in the merged immunotherapy cohorts (n = 321).

(E) Forest plot for the multivariate logistic regression analysis for objective response. AUC, area under the receiver operator characteristic curve; CPS, combined positive score of PDL1 expression; OR, objective response (complete and partial response); SD, stable disease; PD, progressive disease.

STAR★METHODS

Detailed methods are provided in the online version of this paper and include the following:

- KEY RESOURCES TABLE
- RESOURCE AVAILABILITY
  - Lead contact
  - Materials availability
  - Data and code availability
- EXPERIMENTAL MODEL AND SUBJECT DETAILS
  - Study design and patients
- METHOD DETAILS
  - Immunohistochemistry (IHC) staining and scoring
  - CT acquisition and image processing
  - Development of an imaging model to assess IHC-based TME classifier
  - Model architecture and training
  - Visualization and interpretation of network prediction
  - Model validation and comparison with alternative methods
  - Accuracy of the imaging model for TME classification
  - Imaging model's association with prognosis and chemotherapy response
  - Imaging model's association with immunotherapy response
  - Radiogenomics analysis
- QUANTIFICATION AND STATISTICAL ANALYSIS
  - Statistical analysis

SUPPLEMENTAL INFORMATION

Supplemental information can be found online at https://doi.org/10.1016/j.xcrm.2023.101146.

ACKNOWLEDGMENTS

This work was supported in part by Stanford Institute for Human-Centered Artificial Intelligence. The authors would like to thank TCGA/TCIA for providing the genomic and radiology data used in this study.

AUTHOR CONTRIBUTIONS

R.L., G.L., and Y.J. conceived and designed the study; Y.J., Z.S., C.C., W.W., Q.Y., T.L., S.X., and W.X. acquired the data; Y.J., J.X., Z.S., and M.T.I. did the statistical analyses; Y.J., K.Z., H.W., and S.S. developed, trained, and applied the artificial neural network. R.L., W.W., Y.X., and G.L. implemented quality control of data and the algorithms; Y.J., C.C., Q.Y., W.X., and W.W. verified the underlying raw data; all authors had access to the data presented in the manuscript; all authors analyzed and interpreted the data; Y.J., K.Z., Z.S., and H.W. prepared the first draft of the manuscript; R.L. revised the manuscript; and all authors contributed to manuscript preparation.

DECLARATION OF INTERESTS

The authors declare no competing interests.

Received: January 6, 2023
Revised: June 6, 2023
Accepted: July 12, 2023
Published: August 8, 2023

REFERENCES

1. Bray, F., Ferlay, J., Soerjomataram, I., Siegel, R.L., Torre, L.A., and Jemal, A. (2018). Global cancer statistics 2018: GLOBOCAN estimates of incidence and mortality worldwide for 36 cancers in 185 countries. CA A Cancer J. Clin. *68*, 394–424.
2. Jiang, Y., Li, T., Liang, X., Hu, Y., Huang, L., Liao, Z., Zhao, L., Han, Z., Zhu, S., Wang, M., et al. (2017). Association of Adjuvant Chemotherapy With Survival in Patients With Stage II or III Gastric Cancer. JAMA Surg. *152*, e171087.
3. Jiang, Y., Zhang, Q., Hu, Y., Li, T., Yu, J., Zhao, L., Ye, G., Deng, H., Mou, T., Cai, S., et al. (2018). ImmunoScore Signature: A Prognostic and Predictive Tool in Gastric Cancer. Ann. Surg. *267*, 504–513.
4. Fridman, W.H., Pagès, F., Sautès-Fridman, C., and Galon, J. (2012). The immune contexture in human tumours: impact on clinical outcome. Nat. Rev. Cancer *12*, 298–306.
5. Becht, E., de Reyniès, A., Giraldo, N.A., Pilati, C., Buttard, B., Lacroix, L., Selves, J., Sautès-Fridman, C., Laurent-Puig, P., and Fridman, W.H. (2016). Immune and Stromal Classification of Colorectal Cancer Is Associated with Molecular Subtypes and Relevant for Precision Immunotherapy. Clin. Cancer Res. *22*, 4057–4066.
6. Mahajan, U.M., Langhoff, E., Goni, E., Costello, E., Greenhalf, W., Halloran, C., Ormanns, S., Kruger, S., Boeck, S., Ribback, S., et al. (2018). Immune Cell and Stromal Signature Associated With Progression-Free Survival of Patients With Resected Pancreatic Ductal Adenocarcinoma. Gastroenterology *155*, 1625–1639.e2.
7. Jiang, Y., Xie, J., Han, Z., Liu, W., Xi, S., Huang, L., Huang, W., Lin, T., Zhao, L., Hu, Y., et al. (2018). Immunomarker Support Vector Machine Classifier for Prediction of Gastric Cancer Survival and Adjuvant Chemotherapeutic Benefit. Clin. Cancer Res. *24*, 5574–5584.
8. Zeng, D., Zhou, R., Yu, Y., Luo, Y., Zhang, J., Sun, H., Bin, J., Liao, Y., Rao, J., Zhang, Y., and Liao, W. (2018). Gene expression profiles for a prognostic immunoscore in gastric cancer. Br. J. Surg. *105*, 1338–1348.
9. Li, T.J., Jiang, Y.M., Hu, Y.F., Huang, L., Yu, J., Zhao, L.Y., Deng, H.J., Mou, T.Y., Liu, H., Yang, Y., et al. (2017). Interleukin-17-Producing Neutrophils Link Inflammatory Stimuli to Disease Progression by Promoting Angiogenesis in Gastric Cancer. Clin. Cancer Res. *23*, 1575–1585.
10. Yuan, Y. (2016). Spatial Heterogeneity in the Tumor Microenvironment. Cold Spring Harb. Perspect. Med. *6*, a026583.
11. Tomaszewski, M.R., and Gillies, R.J. (2021). The Biological Meaning of Radiomic Features. Radiology *299*, E256–E516.
12. Sun, R., Limkin, E.J., Vakalopoulou, M., Dercle, L., Champiat, S., Han, S.R., Verlingue, L., Brandao, D., Lancia, A., Ammari, S., et al. (2018). A radiomics approach to assess tumour-infiltrating CD8 cells and response to anti-PD-1 or anti-PD-L1 immunotherapy: an imaging biomarker, retrospective multicohort study. Lancet Oncol. *19*, 1180–1191.
13. Jiang, Y., Wang, H., Wu, J., Chen, C., Yuan, Q., Huang, W., Li, T., Xi, S., Hu, Y., Zhou, Z., et al. (2020). Noninvasive imaging evaluation of tumor immune microenvironment to predict outcomes in gastric cancer. Ann. Oncol. *31*, 760–768.
14. Vaidya, P., Bera, K., Gupta, A., Wang, X., Corredor, G., Fu, P., Beig, N., Prasanna, P., Patil, P.D., Velu, P.D., et al. (2020). CT derived radiomic score for predicting the added benefit of adjuvant chemotherapy following surgery in Stage I, II resectable Non-Small Cell Lung Cancer: a retrospective multi-cohort study for outcome prediction. Lancet. Digit. Health *2*, e116–e128.
15. Gulshan, V., Peng, L., Coram, M., Stumpe, M.C., Wu, D., Narayanaswamy, A., Venugopalan, S., Widner, K., Madams, T., Cuadros, J., et al. (2016). Development and Validation of a Deep Learning Algorithm for Detection of Diabetic Retinopathy in Retinal Fundus Photographs. JAMA *316*, 2402–2410.

16. Esteva, A., Kuprel, B., Novoa, R.A., Ko, J., Swetter, S.M., Blau, H.M., and Thrun, S. (2017). Dermatologist-level classification of skin cancer with deep neural networks. Nature *542*, 115–118.

17. Ehteshami Bejnordi, B., Veta, M., Johannes van Diest, P., van Ginneken, B., Karssemeijer, N., Litjens, G., van der Laak, J.A.W.M., the CAMELYON16 Consortium; Hermsen, M., Manson, Q.F., et al. (2017). Diagnostic Assessment of Deep Learning Algorithms for Detection of Lymph Node Metastases in Women With Breast Cancer. JAMA *318*, 2199–2210.

18. Chilamkurthy, S., Ghosh, R., Tanamala, S., Biviji, M., Campeau, N.G., Venugopal, V.K., Mahajan, V., Rao, P., and Warier, P. (2018). Deep learning algorithms for detection of critical findings in head CT scans: a retrospective study. Lancet *392*, 2388–2396.

19. Coudray, N., Ocampo, P.S., Sakellaropoulos, T., Narula, N., Snuderl, M., Fenyö, D., Moreira, A.L., Razavian, N., and Tsirigos, A. (2018). Classification and mutation prediction from non-small cell lung cancer histopathology images using deep learning. Nat. Med. *24*, 1559–1567.

20. Ardila, D., Kiraly, A.P., Bharadwaj, S., Choi, B., Reicher, J.J., Peng, L., Tse, D., Etemadi, M., Ye, W., Corrado, G., et al. (2019). End-to-end lung cancer screening with three-dimensional deep learning on low-dose chest computed tomography. Nat. Med. *25*, 954–961.

21. Jiang, Y., Liang, X., Han, Z., Wang, W., Xi, S., Li, T., Chen, C., Yuan, Q., Li, N., Yu, J., et al. (2021). Radiographical assessment of tumour stroma and treatment outcomes using deep learning: a retrospective, multicohort study. Lancet. Digit. Health *3*, e371–e382.

22. Segal, E., Sirlin, C.B., Ooi, C., Adler, A.S., Gollub, J., Chen, X., Chan, B.K., Matcuk, G.R., Barry, C.T., Chang, H.Y., and Kuo, M.D. (2007). Decoding global gene expression programs in liver cancer by noninvasive imaging. Nat. Biotechnol. *25*, 675–680.

23. Wu, J., Li, X., Teng, X., Rubin, D.L., Napel, S., Daniel, B.L., and Li, R. (2018). Magnetic resonance imaging and molecular features associated with tumor-infiltrating lymphocytes in breast cancer. Breast Cancer Res. *20*, 101.

24. Tang, C., Hobbs, B., Amer, A., Li, X., Behrens, C., Canales, J.R., Cuentas, E.P., Villalobos, P., Fried, D., Chang, J.Y., et al. (2018). Development of an Immune-Pathology Informed Radiomics Model for Non-Small Cell Lung Cancer. Sci. Rep. *8*, 1922.

25. Sun, R., Sundahl, N., Hecht, M., Putz, F., Lancia, A., Rouyar, A., Milic, M., Carré, A., Battistella, E., Alvarez Andres, E., et al. (2020). Radiomics to predict outcomes and abscopal response of patients with cancer treated with immunotherapy combined with radiotherapy using a validated signature of CD8 cells. J. Immunother. Cancer *8*, e001429.

26. Khorrami, M., Prasanna, P., Gupta, A., Patil, P., Velu, P.D., Thawani, R., Corredor, G., Alilou, M., Bera, K., Fu, P., et al. (2020). Changes in CT Radiomic Features Associated with Lymphocyte Distribution Predict Overall Survival and Response to Immunotherapy in Non-Small Cell Lung Cancer. Cancer Immunol. Res. *8*, 108–119.

27. Noh, S.H., Park, S.R., Yang, H.K., Chung, H.C., Chung, I.J., Kim, S.W., Kim, H.H., Choi, J.H., Kim, H.K., Yu, W., et al. (2014). Adjuvant capecitabine plus oxaliplatin for gastric cancer after D2 gastrectomy (CLASSIC): 5-year follow-up of an open-label, randomised phase 3 trial. Lancet Oncol. *15*, 1389–1396.

28. Cheong, J.H., Yang, H.K., Kim, H., Kim, W.H., Kim, Y.W., Kook, M.C., Park, Y.K., Kim, H.H., Lee, H.S., Lee, K.H., et al. (2018). Predictive test for chemotherapy response in resectable gastric cancer: a multi-cohort, retrospective analysis. Lancet Oncol. *19*, 629–638.

29. Cristescu, R., Nebozhyn, M., Zhang, C., Albright, A., Kobie, J., Huang, L., Zhao, Q., Wang, A., Ma, H., Alexander Cao, Z., et al. (2022). Transcriptomic Determinants of Response to Pembrolizumab Monotherapy across Solid Tumor Types. Clin. Cancer Res. *28*, 1680–1689.

30. Braman, N.M., Etesami, M., Prasanna, P., Dubchuk, C., Gilmore, H., Tiwari, P., Plecha, D., and Madabhushi, A. (2017). Intratumoral and peritumoral radiomics for the pretreatment prediction of pathological complete response to neoadjuvant chemotherapy based on breast DCE-MRI. Breast Cancer Res. *19*, 57.

31. Peng, H., Dong, D., Fang, M.J., Li, L., Tang, L.L., Chen, L., Li, W.F., Mao, Y.P., Fan, W., Liu, L.Z., et al. (2019). Prognostic Value of Deep Learning PET/CT-based Radiomics: Potential Role for Future Individual Induction Chemotherapy in Advanced Nasopharyngeal Carcinoma. Clin. Cancer Res. *25*, 4271–4279.

32. Jiang, Y., Yuan, Q., Lv, W., Xi, S., Huang, W., Sun, Z., Chen, H., Zhao, L., Liu, W., Hu, Y., et al. (2018). Radiomic signature of (18)F fluorodeoxyglucose PET/CT for prediction of gastric cancer survival and chemotherapeutic benefits. Theranostics *8*, 5915–5928.

33. Jiang, Y., Chen, C., Xie, J., Wang, W., Zha, X., Lv, W., Chen, H., Hu, Y., Li, T., Yu, J., et al. (2018). Radiomics signature of computed tomography imaging for prediction of survival and chemotherapeutic benefits in gastric cancer. EBioMedicine *36*, 171–182.

34. Beukinga, R.J., Hulshoff, J.B., Mul, V.E.M., Noordzij, W., Kats-Ugurlu, G., Slart, R.H.J.A., and Plukker, J.T.M. (2018). Prediction of Response to Neoadjuvant Chemotherapy and Radiation Therapy with Baseline and Restaging (18)F-FDG PET Imaging Biomarkers in Patients with Esophageal Cancer. Radiology *287*, 983–992.

35. Jiang, Y., Jin, C., Yu, H., Wu, J., Chen, C., Yuan, Q., Huang, W., Hu, Y., Xu, Y., Zhou, Z., et al. (2021). Development and Validation of a Deep Learning CT Signature to Predict Survival and Chemotherapy Benefit in Gastric Cancer: A Multicenter, Retrospective Study. Ann. Surg. *274*, e1153–e1161.

36. Junttila, M.R., and de Sauvage, F.J. (2013). Influence of tumour micro-environment heterogeneity on therapeutic response. Nature *501*, 346–354.

37. Fridman, W.H., Zitvogel, L., Sautès-Fridman, C., and Kroemer, G. (2017). The immune contexture in cancer prognosis and treatment. Nat. Rev. Clin. Oncol. *14*, 717–734.

38. Ligorio, M., Sil, S., Malagon-Lopez, J., Nieman, L.T., Misale, S., Di Pilato, M., Ebright, R.Y., Karabacak, M.N., Kulkarni, A.S., Liu, A., et al. (2019). Stromal Microenvironment Shapes the Intratumoral Architecture of Pancreatic Cancer. Cell *178*, 160–175.e27.

39. Bruni, D., Angell, H.K., and Galon, J. (2020). The immune contexture and Immunoscore in cancer prognosis and therapeutic efficacy. Nat. Rev. Cancer *20*, 662–680.

40. Yu, J., Huang, C., Sun, Y., Su, X., Cao, H., Hu, J., Wang, K., Suo, J., Tao, K., He, X., et al. (2019). Effect of Laparoscopic vs Open Distal Gastrectomy on 3-Year Disease-Free Survival in Patients With Locally Advanced Gastric Cancer: The CLASS-01 Randomized Clinical Trial. JAMA *321*, 1983–1992.

41. Japanese Gastric Cancer Association (2011). Japanese gastric cancer treatment guidelines 2010 (ver. 3). Gastric Cancer *14*, 113–123.

42. In, H., Solsky, I., Palis, B., Langdon-Embry, M., Ajani, J., and Sano, T. (2017). Validation of the 8th Edition of the AJCC TNM Staging System for Gastric Cancer using the National Cancer Database. Ann. Surg Oncol. *24*, 3683–3691.

43. Fang, J.H., Zhou, H.C., Zeng, C., Yang, J., Liu, Y., Huang, X., Zhang, J.P., Guan, X.Y., and Zhuang, S.M. (2011). MicroRNA-29b Suppresses Tumor Angiogenesis, Invasion, and Metastasis by Regulating Matrix Metalloproteinase 2 Expression. Hepatology *54*, 1729–1740.

44. Kikuchi, Y., Kunita, A., Iwata, C., Komura, D., Nishiyama, T., Shimazu, K., Takeshita, K., Shibahara, J., Kii, I., Morishita, Y., et al. (2014). The niche component periostin is produced by cancer-associated fibroblasts, supporting growth of gastric cancer through ERK activation. Am. J. Pathol. *184*, 859–870.

45. Jiang, Y., Xie, J., Huang, W., Chen, H., Xi, S., Han, Z., Huang, L., Lin, T., Zhao, L.Y., Hu, Y.F., et al. (2019). Tumor Immune Microenvironment and Chemosensitivity Signature for Predicting Response to Chemotherapy in Gastric Cancer. Cancer Immunol. Res. *7*, 2065–2073.

CellPress
OPEN ACCESS

46. Wang, J., Sun, K., Cheng, T., Jiang, B., Deng, C., Zhao, Y., Liu, D., Mu, Y., Tan, M., Wang, X., et al. (2021). Deep High-Resolution Representation Learning for Visual Recognition. IEEE Trans. Pattern Anal. Mach. Intell. *43*, 3349–3364.

47. Hu, J., Shen, L., and Sun, G. (2018). Squeeze-and-Excitation Networks. In In 2018 IEEE/CVF Conference on Computer Vision and Pattern Recognition, pp. 7132–7141.

48. van Griethuysen, J.J.M., Fedorov, A., Parmar, C., Hosny, A., Aucoin, N., Narayan, V., Beets-Tan, R.G.H., Fillion-Robin, J.C., Pieper, S., and Aerts, H.J.W.L. (2017). Computational Radiomics System to Decode the Radiographic Phenotype. Cancer Res. *77*, e104–e107.

49. Selvaraju, R.R., Cogswell, M., Das, A., Vedantam, R., Parikh, D., and Batra, D. (2020). Grad-CAM: Visual Explanations from Deep Networks via Gradient-Based Localization. Int. J. Comput. Vis. *128*, 336–359.

50. Nishino, M., Giobbie-Hurder, A., Gargano, M., Suda, M., Ramaiya, N.H., and Hodi, F.S. (2013). Developing a common language for tumor response to immunotherapy: immune-related response criteria using unidimensional measurements. Clin. Cancer Res. *19*, 3936–3943.

51. Subramanian, A., Tamayo, P., Mootha, V., Mukherjee, S., Ebert, B., Gillette, M., Paulovich, A., Pomeroy, S., Golub, T., Lander, E., et al. (2005). Gene set enrichment analysis: a knowledge-based approach for interpreting genome-wide expression profiles. Proc. Natl. Acad. Sci. USA *102*, 15545–15550.

52. Mayakonda, A., Lin, D.C., Assenov, Y., Plass, C., and Koeffler, H.P. (2018). Maftools: efficient and comprehensive analysis of somatic variants in cancer. Genome Res. *28*, 1747–1756.

53. Kowanetz, M., Zou, W., McCleland, M., Gandara, D.R., Gadgeel, S., Rittmeyer, A., Barlesi, F., Park, K., Shames, D., Koeppen, H., et al. (2017). MA 05.09 Pre-Existing Immunity Measured by Teff Gene Expression in Tumor Tissue is Associated with Atezolizumad Efficacy in NSCLC. J. Thorac. Oncol. *12*, S1817–S1818.

54. Fehrenbacher, L., Spira, A., Ballinger, M., Kowanetz, M., Vansteenkiste, J., Mazieres, J., Park, K., Smith, D., Artal-Cortes, A., Lewanski, C., et al. (2016). Atezolizumab versus docetaxel for patients with previously treated non-small-cell lung cancer (POPLAR): A multicentre, open-label, phase 2 randomised controlled trial. Lancet *387*, 1837–1846.

STAR★METHODS

KEY RESOURCES TABLE

REAGENT or RESOURCE	SOURCE	IDENTIFIER
Antibodies		
CD3	NeoMarkers	clone SP7
CD8	NeoMarkers	clone SP16
CD45RO	Invitrogen	clone UCHL1
CD57	NeoMarkers	clone NK1
CD66b	BD Pharmingen	Cat#555723
CD68	Dako,	clone PG-M1
CD34	Abcam,	ab81289
a-SMA	Abcam,	ab5694
Horseradish-peroxidase-conjugated anti-rabbit and anti-mouse antibody	Dako	Code K5007
Software and algorithms		
R 4.1.0	The R Project for Statistical Computing	https://www.r-project.org
GraphPad PRISM 8	GraphPad software	https://www.graphpad.com/
BioRender	BioRender website	https://www.biorender.com/
SPSS software version 21.0	IBM SPSS software	https://www.ibm.com/spss
Python version 3.6	Python software	https://www.python.org/

RESOURCE AVAILABILITY

Lead contact

Further information and requests for resources and reagents should be directed to and will be fulfilled by the lead contact, Ruijiang Li (rli2@stanford.edu).

Materials availability

This study did not generate new unique reagents.

Data and code availability

- The CT image data reported in this study cannot be deposited in a public repository because they contain sensitive information that could compromise patient privacy. Deidentified patient-level clinical and outcome data will be provided upon reasonable request. In addition, summary statistics describing these data have been deposited at supplementary tables and are publicly available as of the date of publication.
- Source code for the deep learning model is available at: https://github.com/MontaEllis/HR-Rad-Net.
- Any additional information required to reanalyze the data reported in this work paper is available from the lead contact upon request.

EXPERIMENTAL MODEL AND SUBJECT DETAILS

Study design and patients

The overall study design is shown in Figure 1. This study followed the Transparent Reporting of a Multivariable Prediction Model for Individual Prognosis or Diagnosis (TRIPOD) reporting guideline. Ethical approval was obtained from the institutional review boards of Nanfang Hospital of Southern Medical University and Sun Yat-sen University Cancer Center (SYSUCC), and patient consent was waived for this retrospective analysis. We retrospectively reviewed data for 5,213 patients who underwent surgery for gastric cancer in two academic medical centers. The inclusion criteria were: histologically confirmed diagnosis of GC; resection of the primary tumor with at least 15 lymph nodes harvested; preoperative abdominal computed tomography (CT) images available; and complete clinicopathological and follow-up data available. We excluded patients who had other synchronous malignant neoplasms, or previously received neoadjuvant chemotherapy; patients whose primary tumor could not be identified on CT were also excluded.

CellPress
OPEN ACCESS

A total of 2,686 patients in six independent cohorts were included in this study (Figure S1; Tables S1 and S2).). The training cohort and two internal validation cohorts included 398, 196, and 602 patients who were consecutively treated at Nanfang Hospital of Southern Medical University (Guangzhou, China) from January 1 2005 to June 30 2009, from July 1 2009 to December 31 2012, and from January 1 2013 to June 30 2017 respectively. The two external validation cohorts included 1,169 patients consecutively treated at SYSUCC (Guangzhou, China) between June 1 2007 and June 30 2013. Here, we divided patients into training and validation cohorts by time of surgery instead of random sampling. This strategy substantially reduces arbitrariness in data splitting, which allows more rigorous assessment and independent validation of the model. Moreover, this approach mimics the situation where a model is first trained on existing data and then tested on future patients.

Clinicopathologic data including age, gender, tumor and lymph node status, tumor differentiation, Lauren histology type, carcinoembryonic antigen (CEA), and cancer antigen 19-9 (CA19-9) was collected. D2 lymph node dissection was performed in most patients (>90%) in accordance with clinical guidelines.[40,41] Tumor staging was performed on the basis of the 8th Edition of the American Joint Committee on Cancer TNM Staging Manual.[42] There were 164 (51.10%), 109 (44.0%), 258 (49.5%), and 525 (46.8%) patients who received 5-fluorouracil–based adjuvant chemotherapy in the training cohort, internal validation cohorts 1 and 2 from Nanfang Hospital, and external validation cohort from SYSUCC, respectively. Among these patients, 171 (32.2%) from Nanfang Hospital and 179 (34.1%) from SYSYCC received the XELOX (capecitabine–oxaliplatin) chemotherapy regimen, while 360 (67.8%) and 346 (65.9%) received the FOLFOX (fluorouracil–folinic acid–oxaliplatin) regimen in the two institutions.

The immunotherapy cohort consists of 321 patients with advanced GC treated at Nanfang Hospital and Guangdong Provincial Hospital of Chinese Medicine (Table S2). Anti-PD-1 drugs include: Nivolumab, Pembrolizumab, or Toripalimab. Clinical data, including patient demographics, treatment information, laboratory and pathologic examinations, and CT scans were acquired. Microsatellite instability (MSI) status was assessed by either IHC or DNA sequencing.

METHOD DETAILS

Immunohistochemistry (IHC) staining and scoring

Formalin-fixed paraffin-embedded (FFPE) samples were cut into 4-μm thick sections, which were then processed for immunohistochemistry as previously described.[3,7] The samples were de-waxed in xylene and rehydrated in decreasing concentrations of ethanol. Prior to staining, the sections were subjected to endogenous peroxidase blocking in 1% H_2O_2 solution diluted in methanol for 10 min and then heated in a microwave for 30 min with 10 mmol/L citrate buffer (pH 6.0). Serum blocking was performed using 10% normal rabbit serum for 30 min. The slides were incubated overnight with an antibody against human immune cell biomarkers (CD3 (pan T cells), CD8 (cytotoxic T cells), CD45RO (memory T cells), CD45RA (naive T cells), CD57 (natural killer cells), CD68 (macrophages), CD66b (neutrophils)), a microvascular marker (CD34), and a stromal marker (a-SMA) at 4°C, followed by incubation with an amplification system with a labeled polymer/HRP (EnVision, DakoCytomation, Denmark) at 37°C for 30 min. The reaction was visualized using diaminobenzidine (DAB)+ chromogen, and nucleus was counterstained using hematoxylin. In all assays, we included negative control slides with the primary antibodies omitted. Every staining run contained a slide of positive control. And all slides were stained with DAB dyeing for the same time for each antibody (Table S8).

As previously described,[7] we calculated a machine learning classifier to assess the overall TME status based on the expression of eight IHC markers, including CD3 IM, CD3 TC, CD8 IM, CD45RO TC, CD57 IM, CD68 TC, CD66b IM and CD34 (IM: invasive margin, TC: tumor core). Patients were classified into two groups: TME-high vs. TME-low.

The IHC markers were evaluated independently by two gastrointestinal pathologists who were blinded to the clinical data. A third pathologist was consulted to reach a consensus when different opinions arose between the two primary pathologists. In detail, the tissue sections were screened at low power (100×) using an inverted research microscope (model DM IRB; Leica, Germany), and 5 most representative fields were selected. The density of immune cells was measured at 200× magnification for two respective areas at tumor core (TC) and invasive margin (IM). The nucleated stained cells in each area were quantified and expressed as the number of cells per field. For micro-vessels, any discrete cluster or single cell stained positive for CD34 was counted as one micro-vessel.[9,43] For the stromal marker (a-SMA), stain intensity was graded as 0 (negative staining), 1 (weak staining), 2 (moderate staining), and 3 (strong staining); stain extent was graded as 0 (0%–4%), 1 (5%–24%), 2 (25%–49%), 3 (50%–74%), and 4 (>75%).[44,45] Values of the stain intensity and extent were multiplied and then averaged over the five fields as the final score.

CT acquisition and image processing

All patients underwent contrast-enhanced abdominal CT scans prior to surgery. Following intravenous contrast administration, arterial and portal venous-phase contrast-enhanced CT scans were performed after delays of 28 s and 60 s, respectively. Iodinated contrast material in the amount of 90–100 mL (Ultravist 370, Bayer Schering Pharma, Berlin, Germany) was injected at a rate of 3.0 or 3.5 mL/s with a pump injector (Ulrich CT Plus 150, Ulrich Medical, Ulm, Germany). The type of CT scanners included GE Lightspeed 16, GE Healthcare Milwaukee, WI; 64-section LightSpeed VCT, GE Medical Systems, Milwaukee, WI; USA. The CT acquisition protocols were as follows: 120 kV; 150–190 mAs; 0.5- or 0.4-s rotation time. Contrast-enhanced CT was reconstructed with a field of view, 350 × 350 mm; data matrix, 512 × 512; in-plane spatial resolution 0.607–0.75 mm; axial slice thickness, 1.25–7.5 mm.

CT images were resampled to a consistent spatial resolution of 0.75 × 0.75 × 2.5 mm by using trilinear interpolation. We normalized the CT intensity to a window of [-150, 150] HU to highlight the soft-tissue contrast. To focus analysis on the most relevant region (i.e., gastric carcinoma), we delineated the primary tumor as the region of interest.

CT images at the portal venous phase were analyzed given its better contrast. The primary tumor was delineated by two radiologists (C.C. and Q.Y. with 11 and 10 years of clinical experience in abdominal CT interpretation, respectively) using the ITK-SNAP software. Both radiologists reached consensus regarding tumor delineation.

Development of an imaging model to assess IHC-based TME classifier

We trained a deep learning radiomics model (named "HR-Rad-Net") to predict the IHC-based TME classifier using CT images. The input image to the model consists of three channels: the original full CT image, CT image with tumor mask, and the binary tumor mask, all with a size of 64 × 64. To leverage the respective advantages of knowledge-based radiomics and data-driven deep learning approaches, we combined the two components in a unifying model (Figure S2A). This idea is motivated by prior work showing that either approach can extract useful information for assessing TME.[12,13,33,35] The proposed model consists of two parts: feature representation learning via convolutional neural network and radiomic feature extraction. HR-Rad-Net draws on HR-Net's ability to effectively learn reliable high-resolution representations. Here, we incorporate a squeeze and excitation (SE) module into HR-Net to better extract multi-scale image features (Figure S2B).[46,47] The squeeze operation utilizes average pooling to extract the global information from each channel of the feature; while the excitation operation adopts a multi-layer perceptron to learn channel-wise weights to reweight each channel.

For radiomics analysis, we computed a total of 361 radiomics features, including 9 shape features, 72 first-order statistical features, 96 Gray Level Cooccurrence Matrix (GLCM) features, 64 Gray Level Run Length Matrix (GLRLM) features, 64 Gray Level Size Zone Matrix (GLSZM) features, and 56 Gray Level Dependence Matrix (GLDM) features.[48] Then, the radiomics information of the CT image and annotation information was calculated and embedded into the feature space via a full connection layer. Given the prohibitive cost of manually delineating full 3D tumor contours for over 2600 patients in the study, these radiomics features were computed based on the image slice containing the largest tumor size.

The network learned features and radiomics features are fused in a fully connected layer to generate the final prediction. Both types of features are standardized by the *Z* score method before being fed into the fully connected layer. To demonstrate effectiveness of the proposed approach, we performed ablation experiments and compared the performance with the models trained without radiomics or SE module separately. Finally, to investigate and visualize which areas in the image are important for prediction, we used the gradient-weighted class activation mapping approach.[49]

Model architecture and training

We propose an HR-Radiomics-Net to predict the TME status of gastric tumors from CT images. The proposed model consists of two parts: feature representation learning via convolutional neural network and radiomic feature extraction, which are fused in a fully connected layer to generate the final prediction (Figure S2). We used a cohort of 398 patients from Nanfang Hospital, Southern Medical University, China for training purposes.

To reduce overfitting, we applied data augmentation and learning rate decay. Specifically, the augmentation included image reflection along the patient's anterior/posterior or left/right directions and random rotation with an angle sampled from (−30°, +30°). We used focal loss as the objective function which is defined as:

$$\mathrm{FL}(p_t) = -\alpha(1 - p_t)^{\gamma}\log(p_t)$$

where p_t is the output of the network, α, γ are set to balance the positive and negative samples.

In this study, we set α to 1 and γ to 2. The batch size was set to 16, the learning rate was set to 1e-3 and the learning rates decay half per 10 epochs. The optimizer used for training was the Adam algorithm. The proposed HR-Radiomics-Net was implemented on the open source Pytorch platform and trained using an NVIDIA GTX 1080TI.

Visualization and interpretation of network prediction

After training the network, we visualize the heatmap of the network that shows the more important features of the image for the model prediction. To achieve this, we use the Guided Grad-CAM.[49] Guided Grad-CAM generates a corresponding heatmap of the input image, which indicates how much the position contributes to the classification. In addition, Guided Grad-CAM can also give a guide based on the returned gradient map, which provides a visualization of the network classification at a fine-grained level. (Figure S3)

Model validation and comparison with alternative methods

We tested the model performance in the internal and external validation cohorts. To demonstrate effectiveness of the proposed approach, we performed ablation experiments and compare the performance with the radiomics and deep learning module trained separately, as shown in Figures S5.

Accuracy of the imaging model for TME classification

We evaluated the accuracy of the CT imaging-based model to assess the TME classifier defined by IHC. Metrics including the area under the receiver operating characteristic curve (AUC), overall accuracy, sensitivity, and specificity were computed. The optimal cutoff value for the deep learning radiomics signature (DLRS) was determined using Youden's index in the training cohort, which maximizes the sum of sensitivity and specificity. This analysis was performed for patients in the training cohort, internal validation cohort 1, and external validation cohort 1 for which IHC data was available. Calibration plots were used to graphically represent the agreement between the predicted and actual probability of the TME classifier. A decision curve analysis was performed to evaluate the model's clinical usefulness by quantifying the net benefit at various threshold probabilities.

Imaging model's association with prognosis and chemotherapy response

We assessed the imaging model's association with survival outcomes including disease-free survival (DFS) and overall survival (OS). DFS was defined as the time from surgery to disease progression or death. OS was defined as the time to death from any cause. This analysis was performed for all 2,365 patients in 5 independent cohorts for which outcome data were available. Additionally, we evaluated the prognostic value in patient subgroups as defined by clinicopathological factors.

We further assessed the association between DLRS and adjuvant chemotherapy response in patients with stage II and III gastric cancer. To minimize potential selection bias and confounding effects, we used a matching strategy to balance patients in each DLRS-defined group. Propensity score matching (PSM) was performed for patients who received vs. did not receive chemotherapy using 1:1 nearest matching. The following variables were matched: age, gender, differentiation, CEA, CA19-9, location, depth of invasion (T stage), lymph node metastasis (N stage), tumor size, and Lauren type.

Imaging model's association with immunotherapy response

We finally assessed the image model's relation to clinical response and outcomes using an independent cohort of 321 advanced GC patients treated with anti-PD-1 immune checkpoint blockade. Response to immunotherapy was evaluated according to the irRECIST criteria[50] and defined as complete response (CR), partial response (PR), stable disease (SD), or progressed disease (PD). Objective response was defined for patients who achieved either CR or PR. Progression-free survival (PFS) was calculated from the start of treatment until disease progression, death, or last follow up. The combined positive score (CPS) was defined as the total number of PD-L1 positive cells (tumor, lymphocytes, and macrophages) divided by the number of tumor cells. CPS was categorized as high ($CPS \geq 10$), intermediate ($10 > CPS \geq 1$), and low ($CPS < 1$).

Radiogenomics analysis

We used the TCGA/TCIA-STAD dataset for radiogenomics analysis, which contains publicly available genomic/transcriptomic data and matched CT images for 42 gastric cancer patients. We processed the CT images to compute DLRS and performed gene set enrichment analyses to identify the underlying molecular pathways associated with the DLRS. We identified genes that are significantly correlated with the imaging signature by the Spearman's rank test. Multiple testing will be corrected using the Benjamini-Hochberg method. To elucidate the biological meaning of DLRS, we performed the gene set enrichment analysis (GSEA) and gene ontology (GO) kyoto encyclopedia of genes and genomes (KEGG) analyses using the GSEA software (version 4.1.0).[51] All parameters were set to their default values, and an adjusted P-value of <0.05 was considered statistically significant.

We further assessed the relationship between the DLRS and established biomarkers of immunotherapy response using patients in the TCGA/TCIA dataset. The PD-L1 and GZMB expression values were determined based on the transcriptomic data from the TCGA. Tumor mutation burden (TMB) was computed using the R package "Matfools".[52] Furthermore, two well established T effector signatures were evaluated, namely, OAK-T effector signature (based on PD-L1, CXCL9, IFNG)[53] and POPLAR-T effector signature (based on CD8A, GZMA, GZMB, IFNG, PD-L1, EOMES, CXCL9, CXCL10, and TBX21).[54]

QUANTIFICATION AND STATISTICAL ANALYSIS

Statistical analysis

We compared two groups using the *t*-test for continuous variables and the chi-square test or Fisher exact test for categorical variables, as appropriate. Survival curves were generated according to the Kaplan-Meier method and compared using the log rank test. Univariate and multivariate analyses were performed using the Cox proportional hazards model. Interaction between the imaging model and adjuvant chemotherapy was assessed by means of the Cox model. Statistical analysis was conducted with R software (version 4.1.0) and SPSS software (version 21.0). A two-sided p value <0.05 was considered statistically significant.

Cell Reports Medicine, Volume *4*

Supplemental information

Non-invasive tumor microenvironment evaluation

and treatment response prediction

in gastric cancer using deep learning radiomics

Yuming Jiang, Kangneng Zhou, Zepang Sun, Hongyu Wang, Jingjing Xie, Taojun Zhang, Shengtian Sang, Md Tauhidul Islam, Jen-Yeu Wang, Chuanli Chen, Qingyu Yuan, Sujuan Xi, Tuanjie Li, Yikai Xu, Wenjun Xiong, Wei Wang, Guoxin Li, and Ruijiang Li

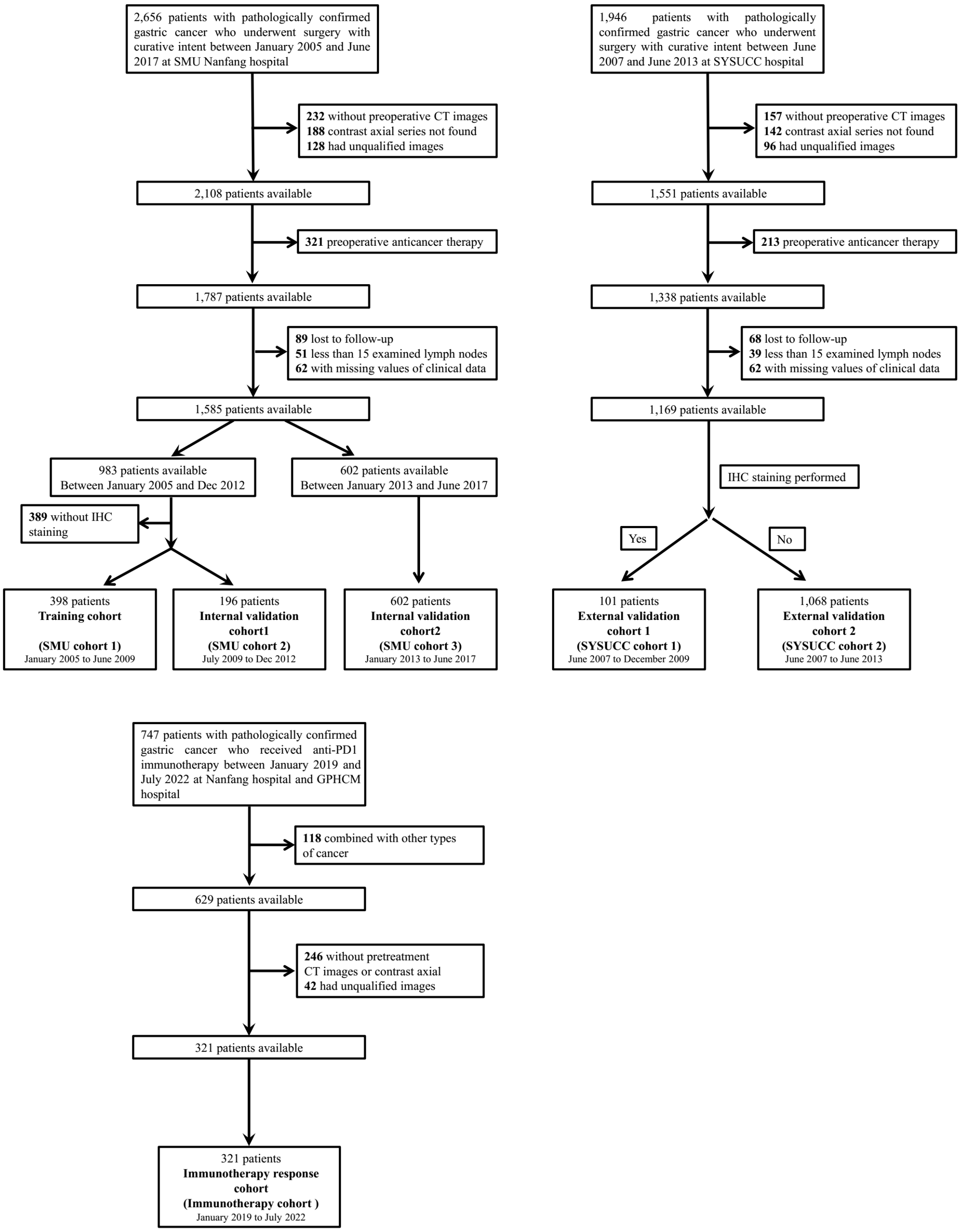

Figure S1. Flow chart of patient inclusion and exclusion. Related to Figure 1.

SMU cohorts: patients from Southern Medical University hospital; SYSUCC cohorts: patients from Sun Yat-sen University Cancer Center; Stanford cohort: patients from Stanford University hospital. Immunotherapy cohort: patients from Guangdong Provincial Hospital of Chinese Medicine (GPHCM).

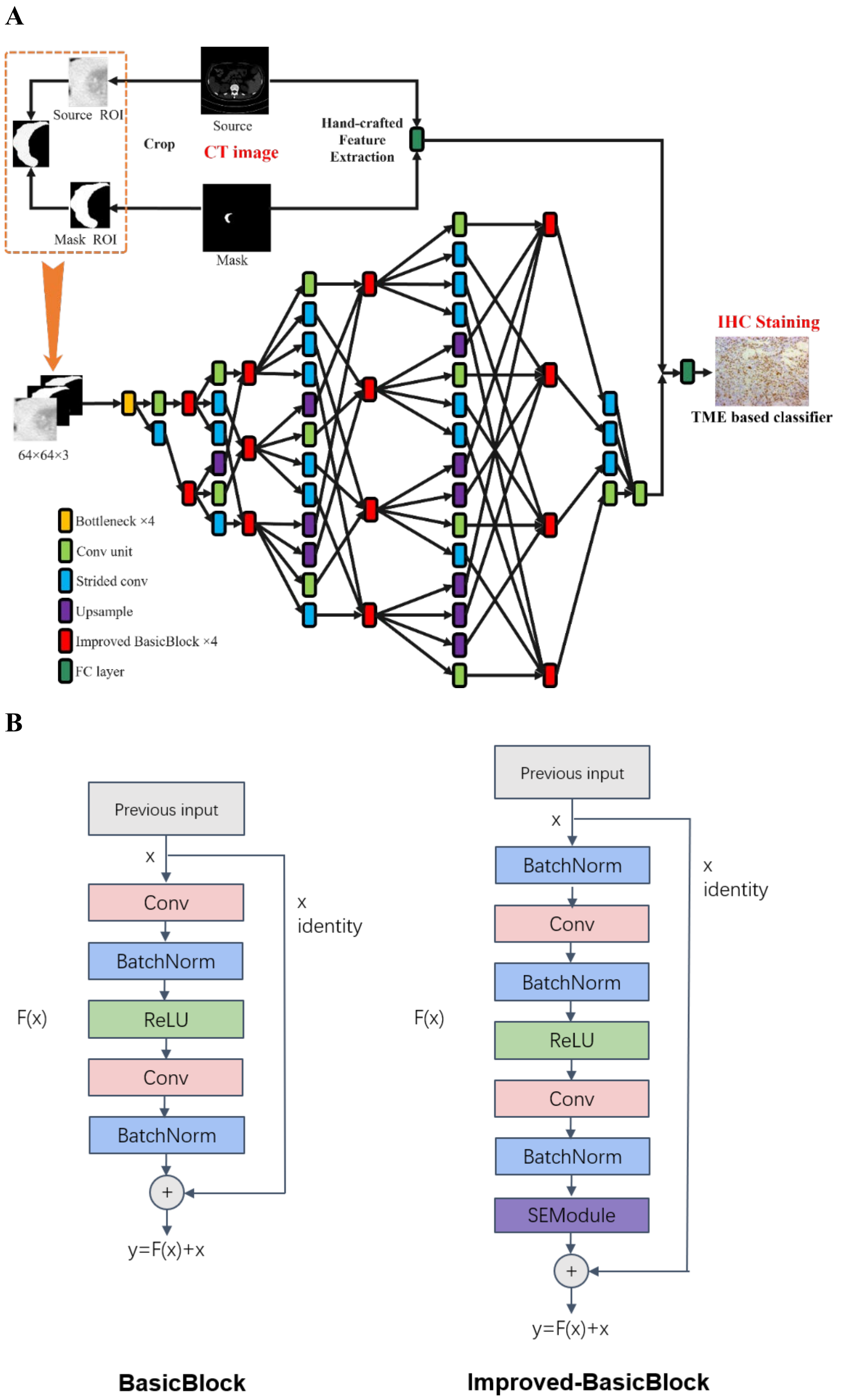

Figure S2. The framework of the proposed network (A) and comparison of basic residual network blocks, SE-based improved residual network blocks, and HR-Rad-Net approach (B). Related to Figure 1.

(A) The radiomics features extracted from the source and mask images are fused with the network

features extracted from improved HR-Net. Different colored, directional arrows represent different processing methods.

(B) The SE-based improvement module incorporates a channel attention mechanism, making the network more focused on learning implicit information between channels.

A

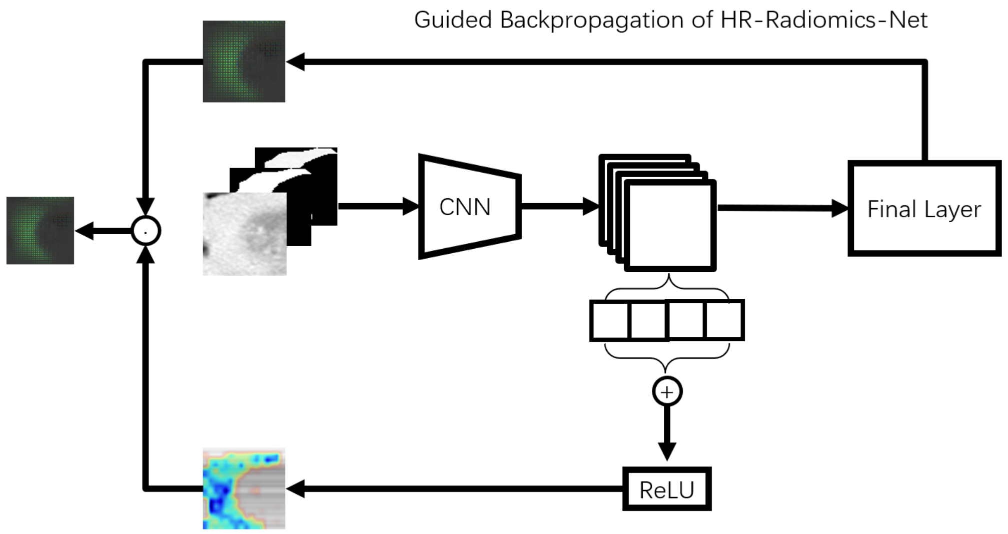

B

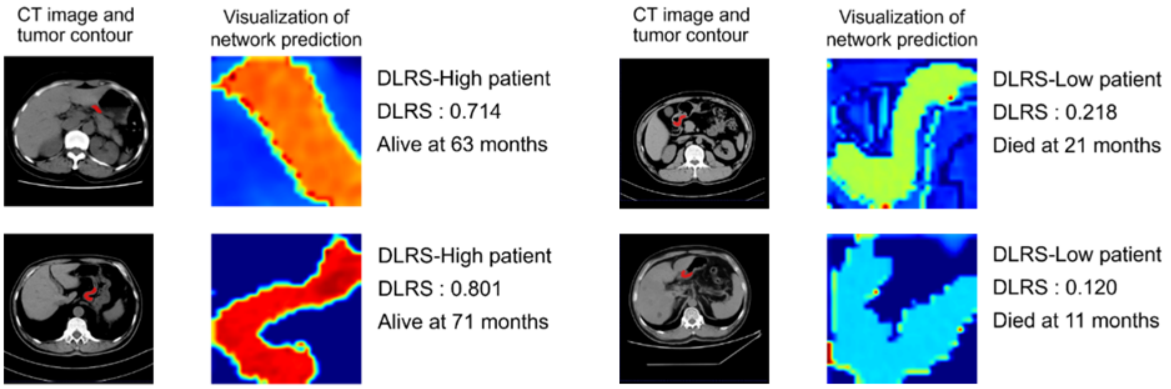

Figure S3. **Guided Grad-CAM for visualization of network prediction. Related to Figure 1.**

(A) The back-propagation of the HR-Radiomics-Net highlights certain areas of the feature map. These feature maps are combined to calculate heatmaps, which show the sub-regions of the network that are focused on learning. At the same time, we combine the guide map of the back-propagation and feature heatmaps to obtain a visualization of the Guided Grad-CAM.

(B) Examples of the CT images, visualization of the HR-Radiomics-Net prediction, and corresponding network prediction (DLRS). Areas in the CT image that are important for prediction of TME class were identified and highlighted by the Grad-CAM approach. Grad-CAM: gradient-weighted class activation mapping.

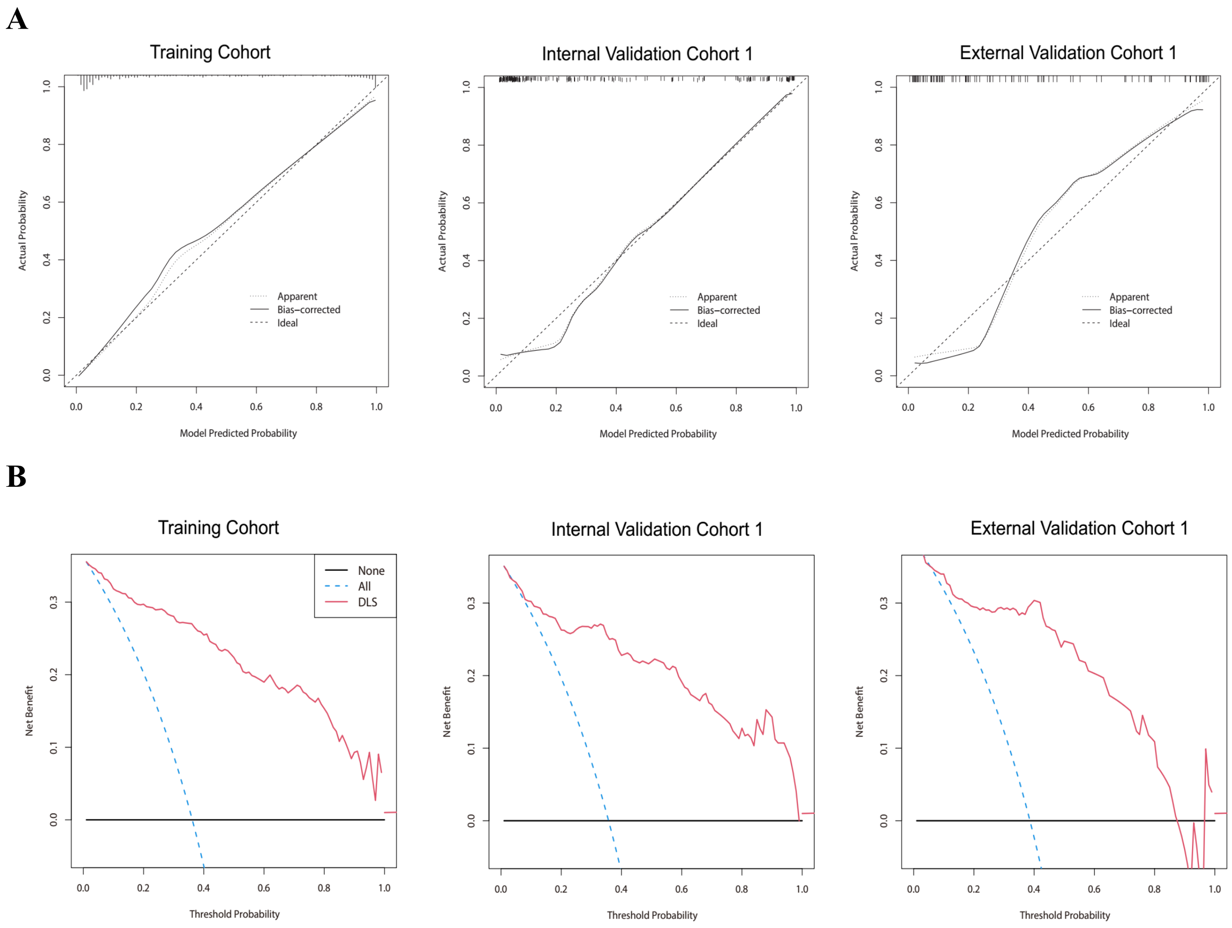

Figure S4. Calibration curves (A) and decision curve (B) analysis for the DLRS model. Related to Figure 2.

(A) The calibration showed the agreement between the estimated and the observed probabilities of the TME classifier. DLRS model-estimated probabilities is plotted on the x-axis; the actual probabilities is plotted on the y-axis. The diagonal dotted line is a perfect estimation by an ideal model, in which the estimated outcome perfectly corresponds to the actual outcome. The solid line is the performance of the DLRS model: a closer alignment with the diagonal dotted line represents a better estimation.

(B) Decision curve analysis of the DLRS model for predicting the TME classifier in the training and validation cohorts. The y-axis measures the net benefit, and the red line represents the DLRS model.

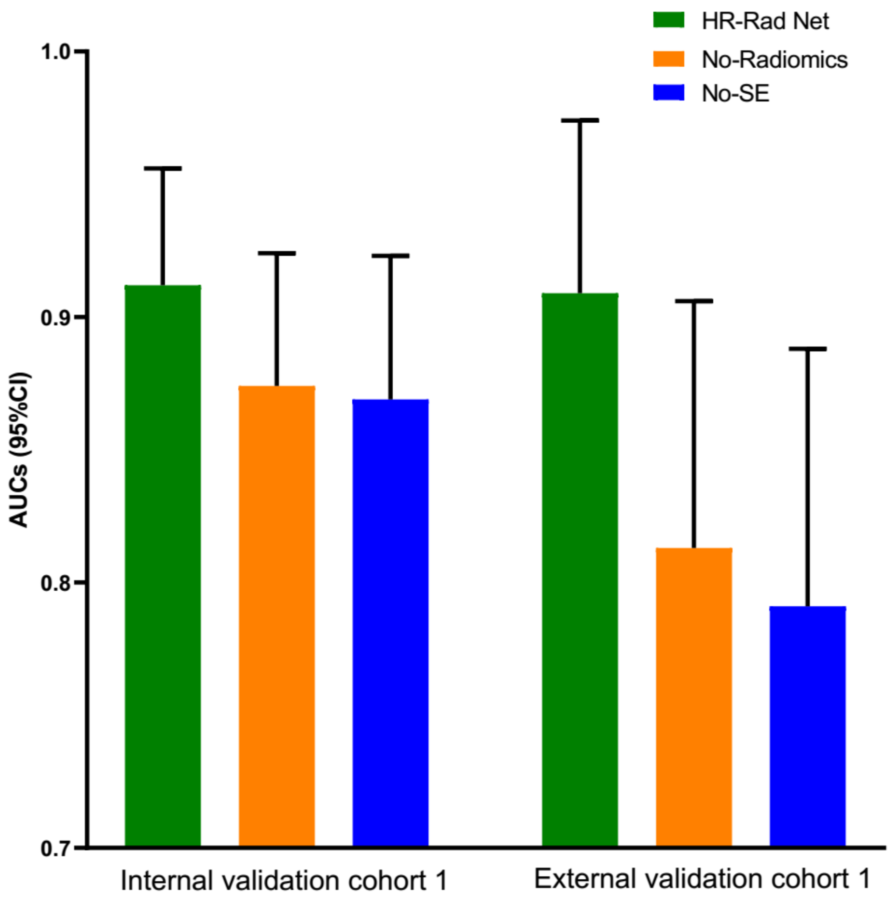

Figure S5. Comparison of the HR-Rad-Net approach with other approaches for prediction of TME-based classifier. Related to Figure 1. No-Radiomics: the model developed without combing with radiomics features. No-SE：the model developed without SE strategy.

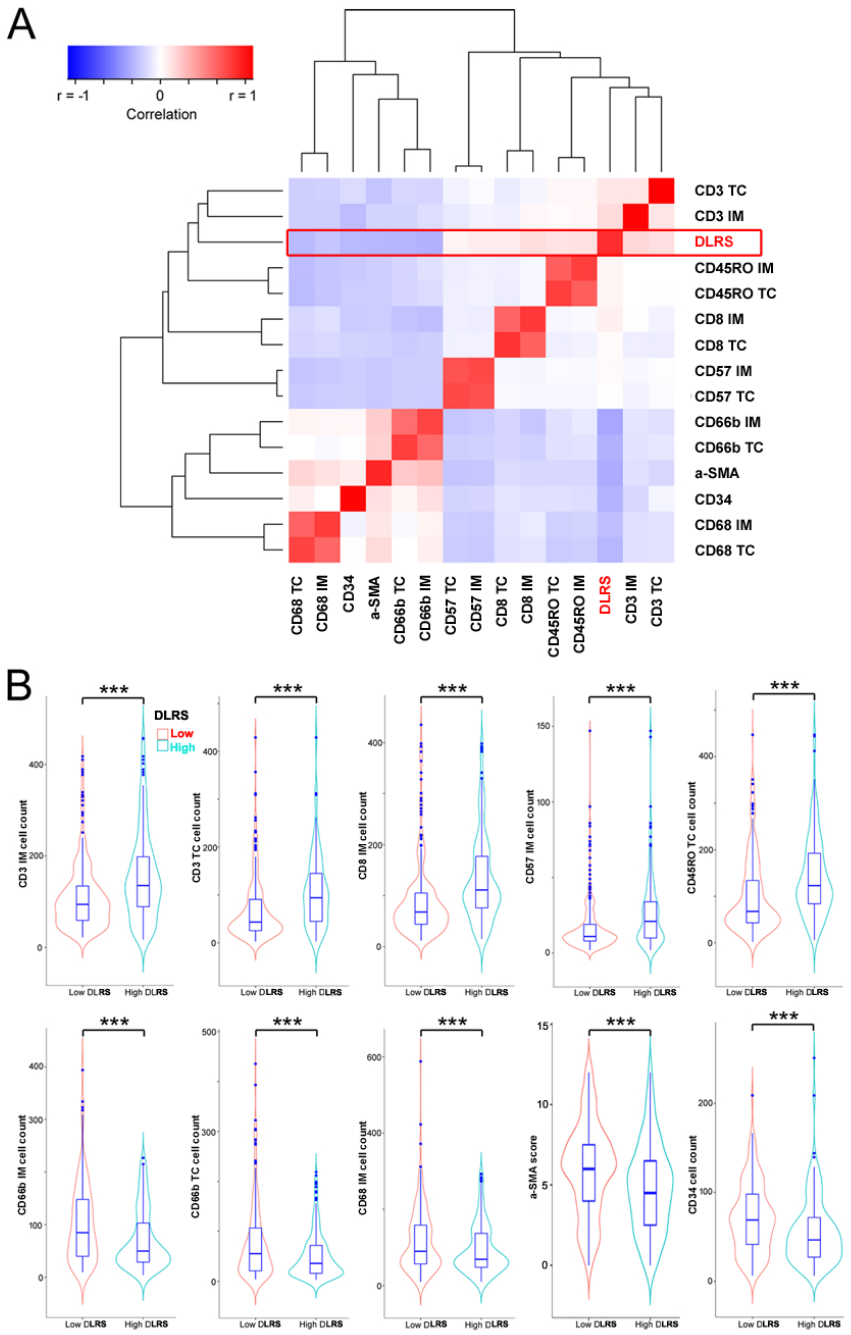

Figure S6. Relationship between the imaging model and individual TME features. Related to Figure 2.

(A) Pairwise correlation between the DLRS and TME features followed by unsupervised hierarchical clustering (Pearson uncentered algorithm).

(B)Violin plots showing association between DLRS group and TME features.

Training cohort

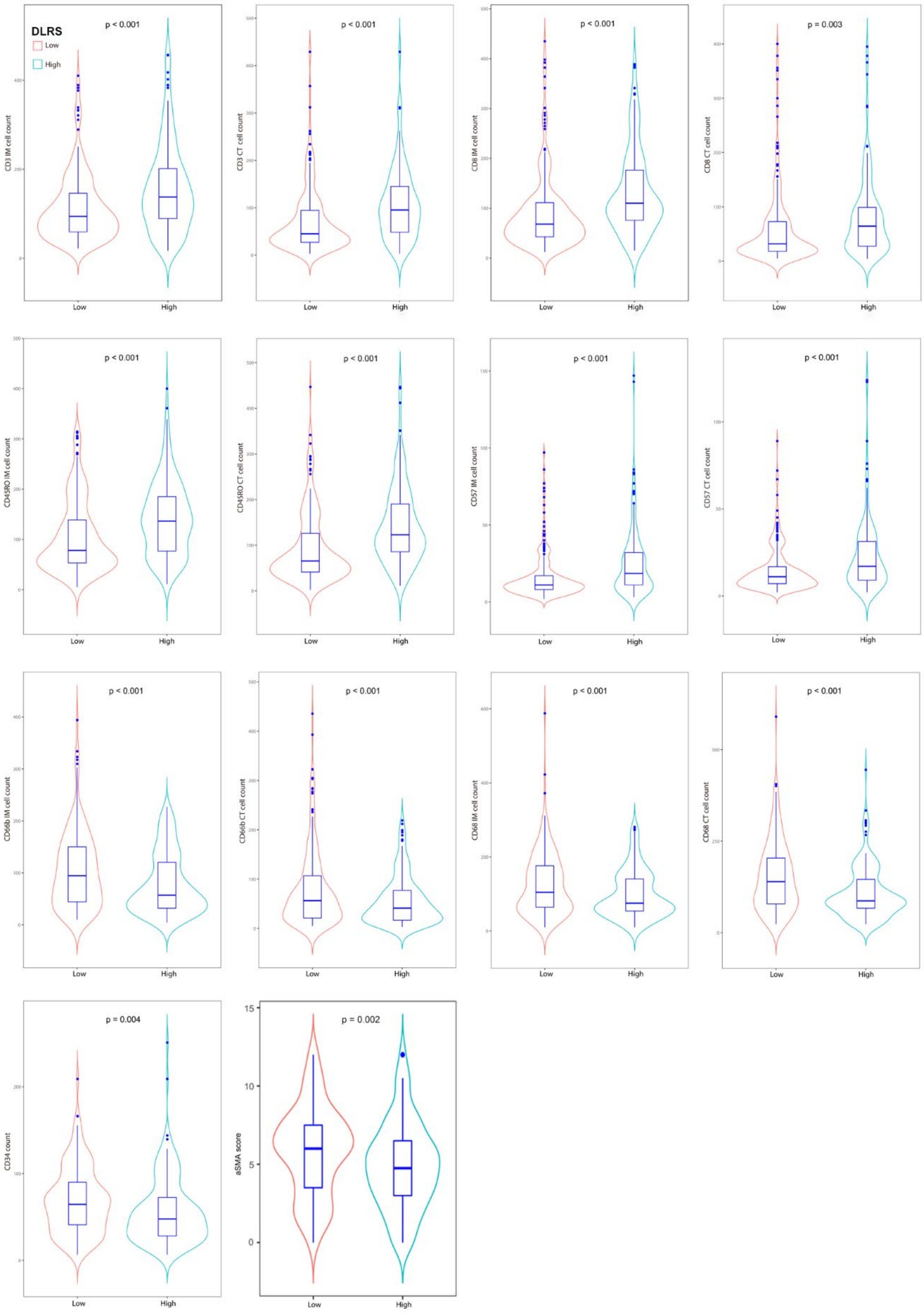

Figure S7. The relationship between DLRS and each TME features in the traing cohort. Related to Figure 2.

Internal validation cohort 1

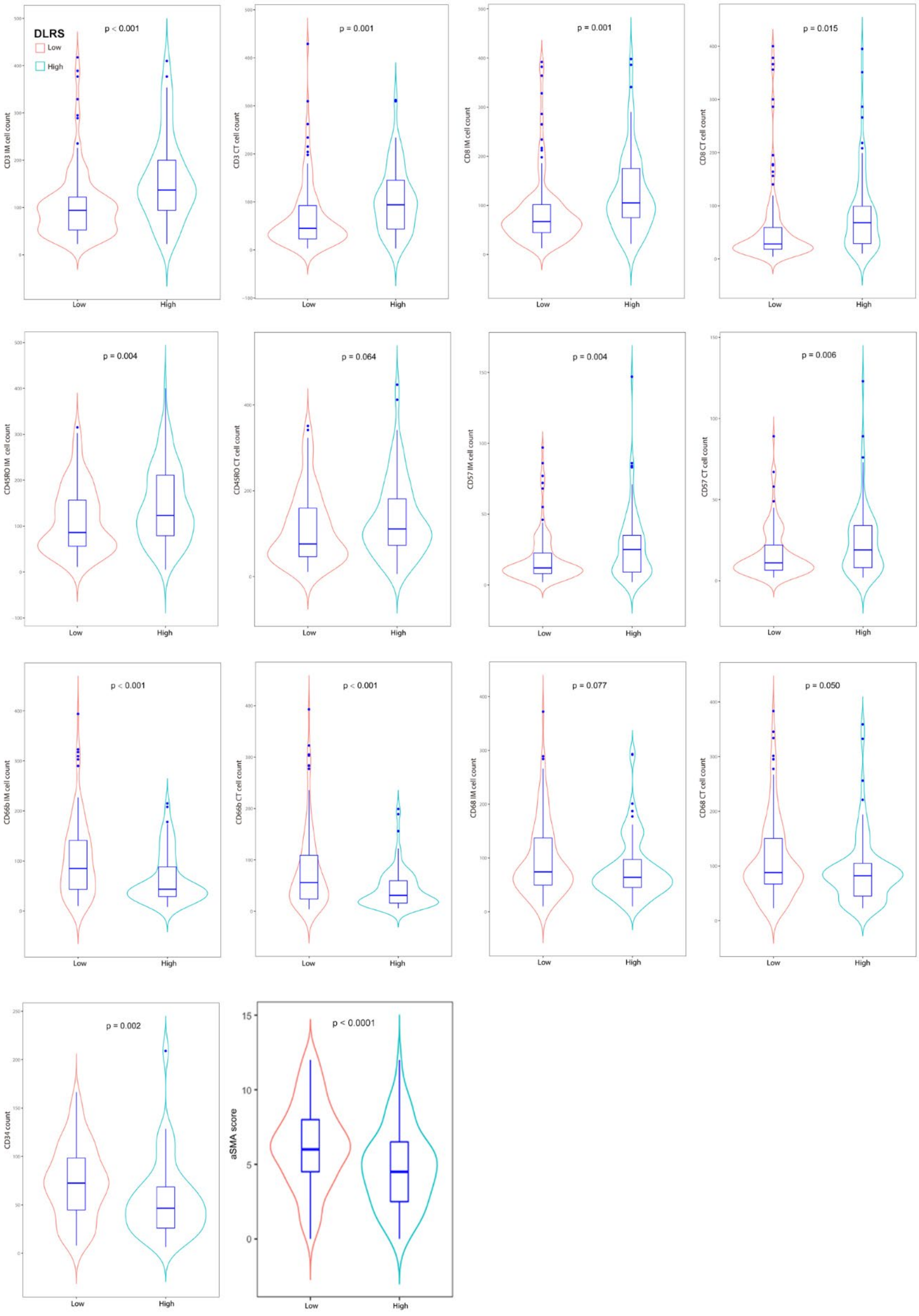

Figure S8. The relationship between DLRS and each TME features in the internal validation cohort 1. Related to Figure 2.

External validation cohort 1

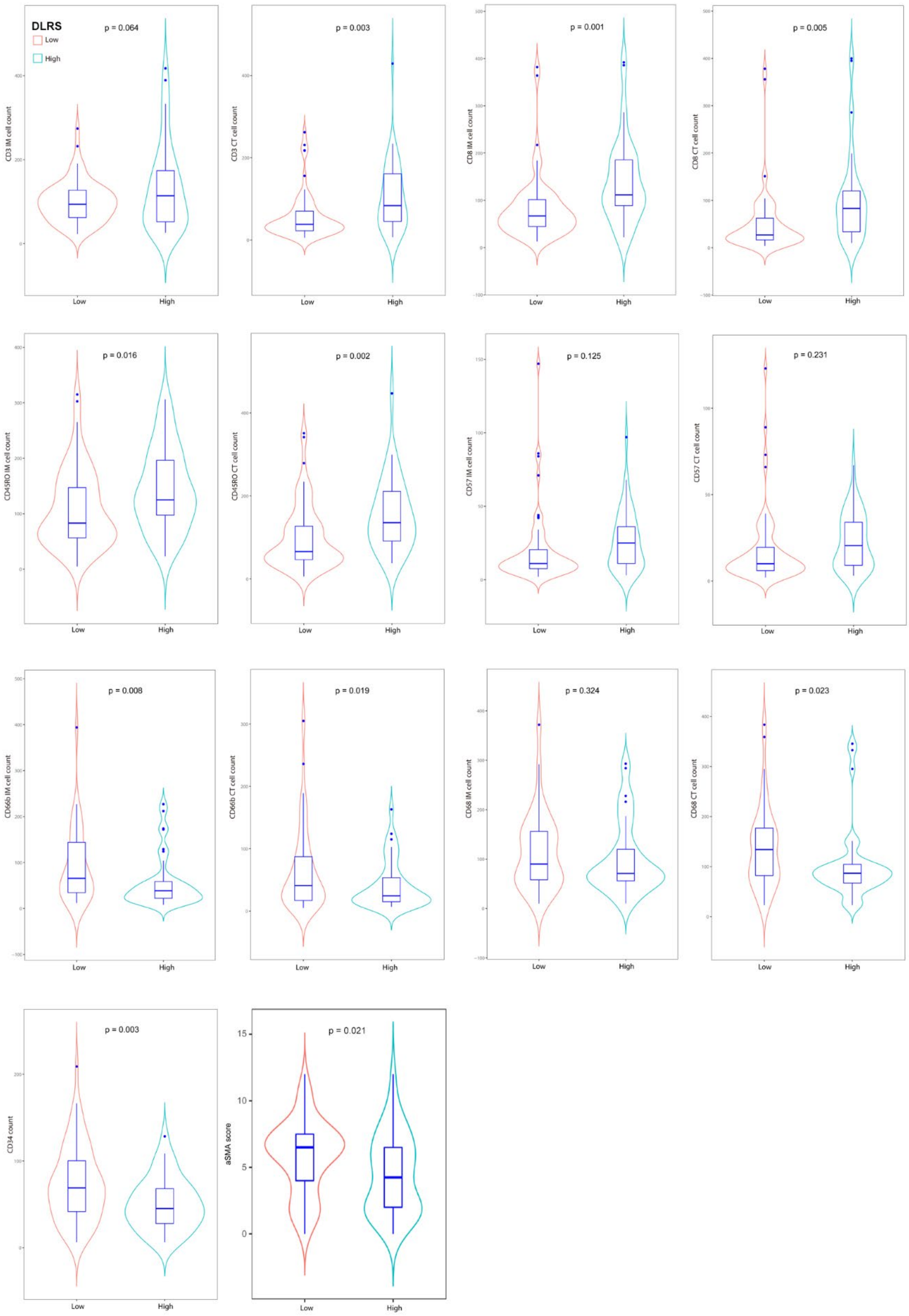

Figure S9. The relationship between DLRS and each TME features in the external validation cohort 1. Related to Figure 2.

Training cohort

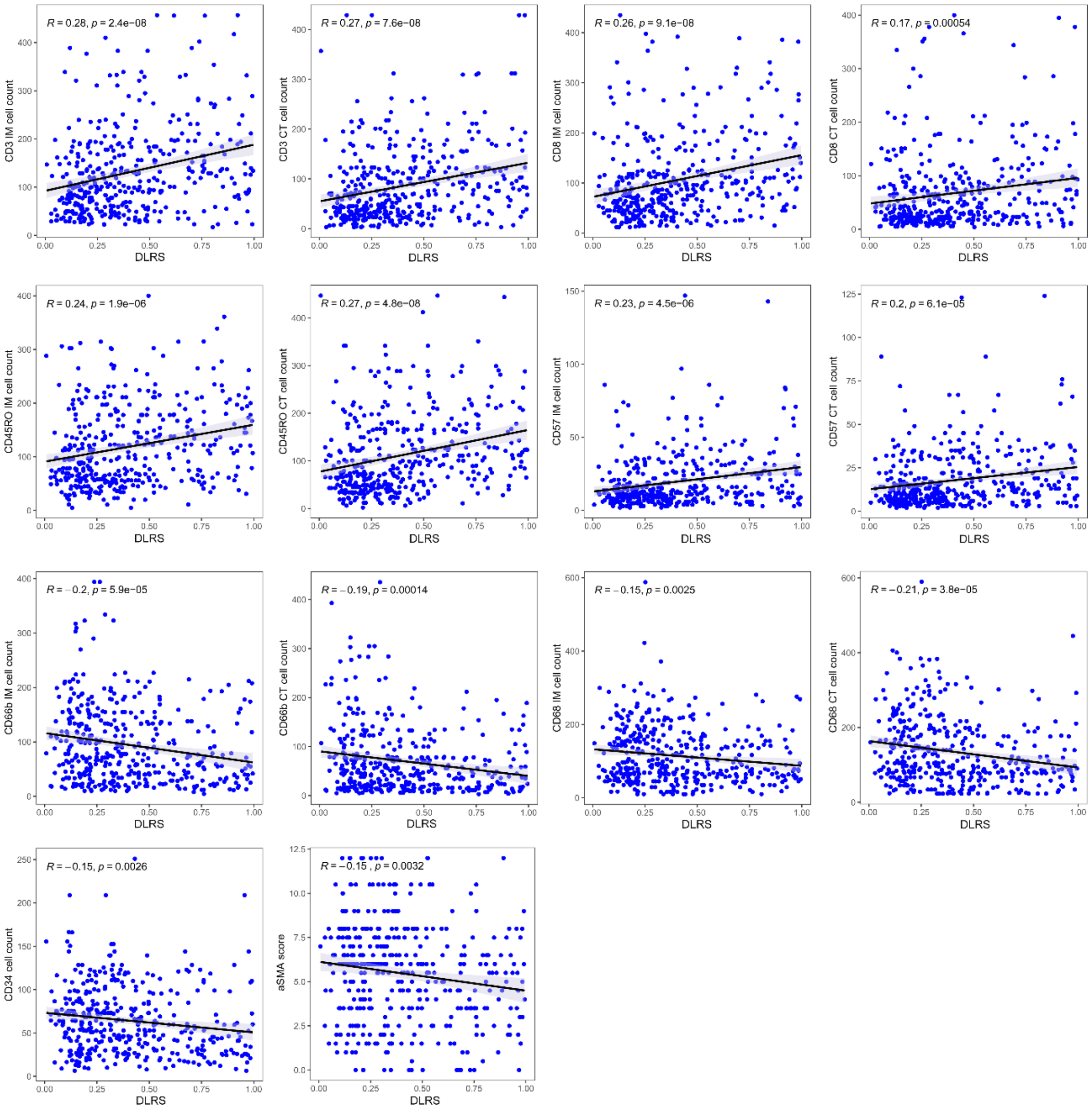

Figure S10. The scatter plot of relationship between DLRS and each TME features in the training cohort. Related to Figure 2.

Internal validation cohort 1

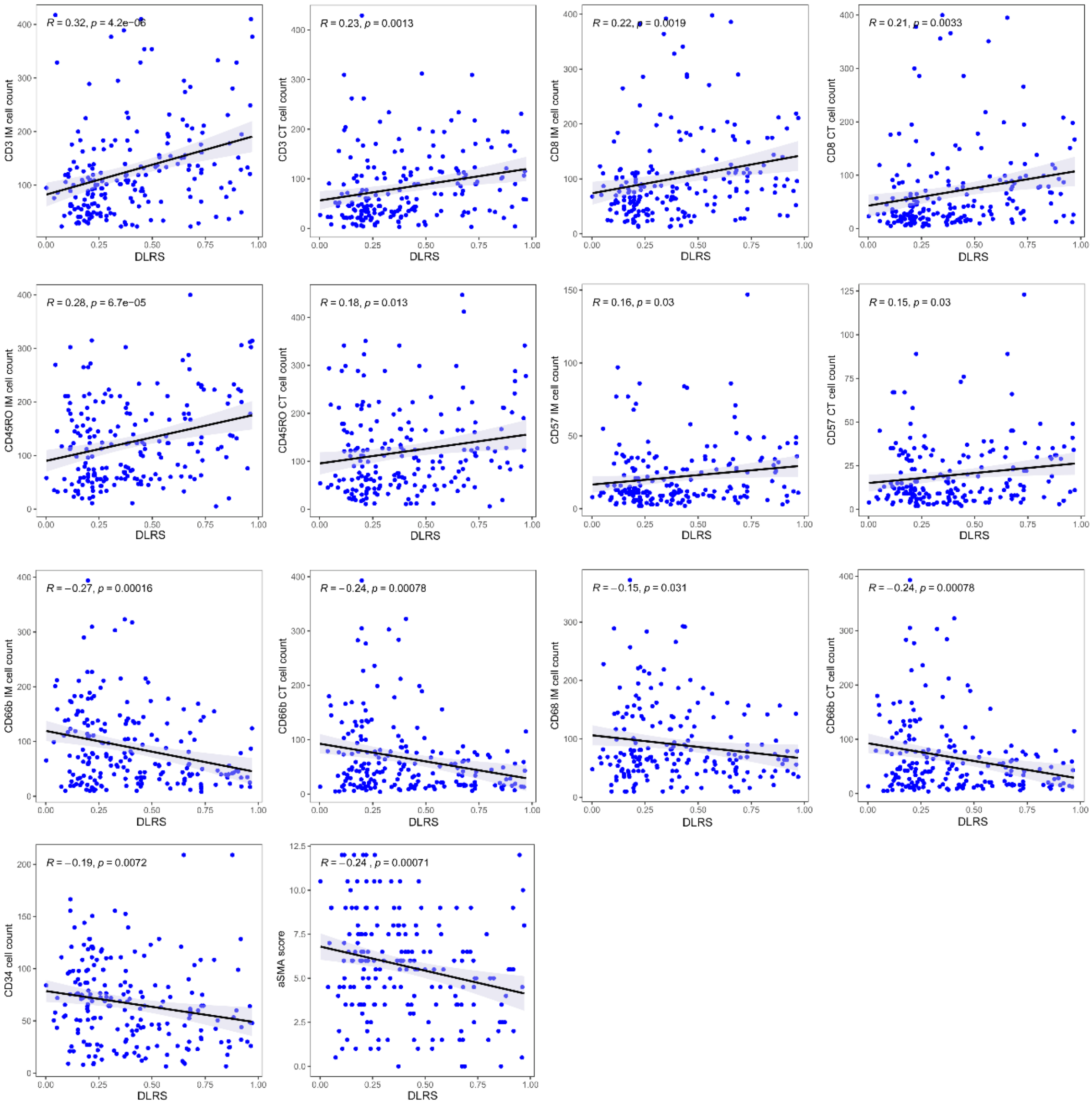

Figure S11. The scatter plot of relationship between DLRS and each TME features in the internal validation cohort 1. Related to Figure 2.

External validation cohort 1

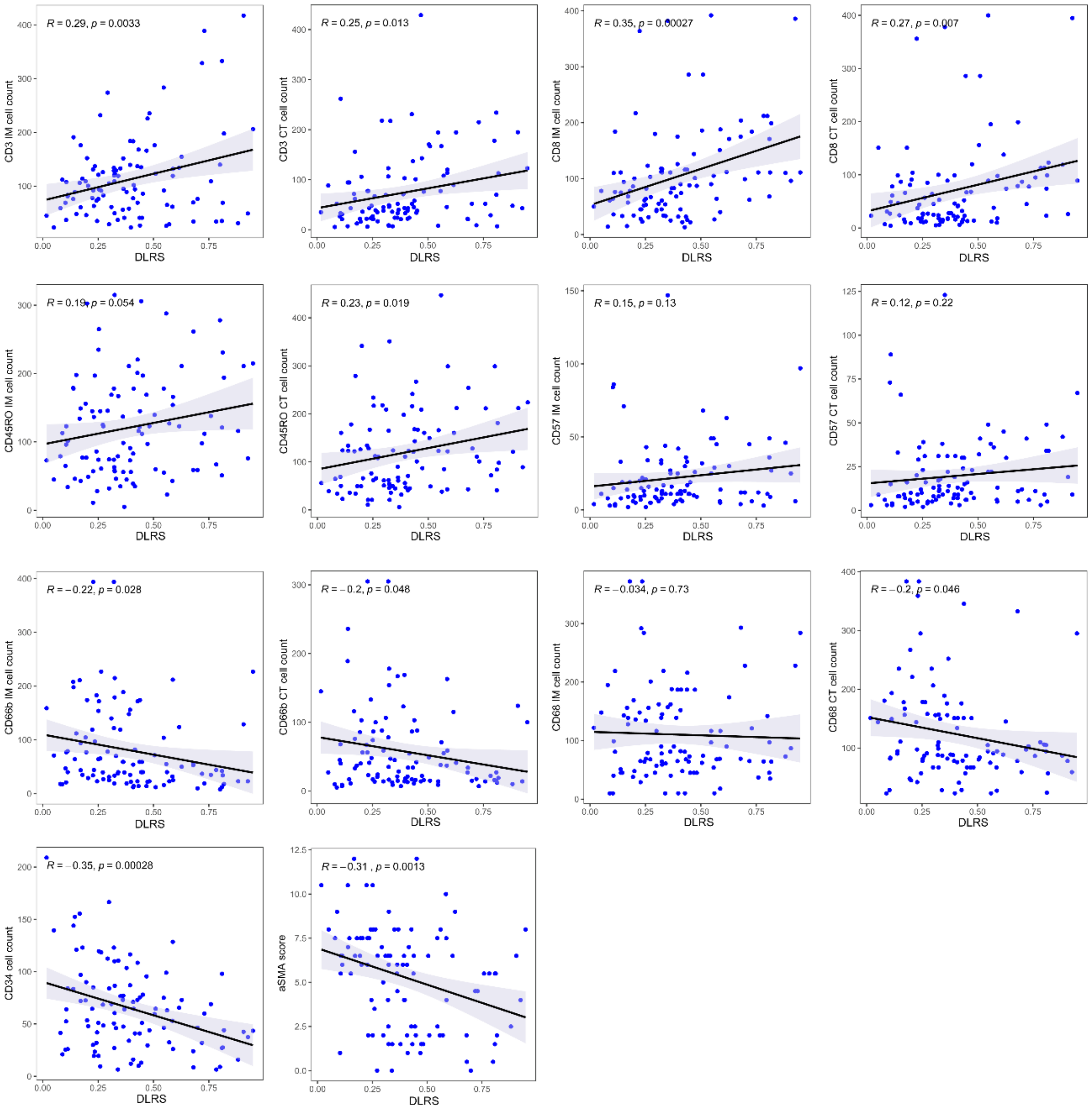

Figure S12. The scatter plot of relationship between DLRS and each TME features in the external validation cohort 1. Related to Figure 2.

Training cohort

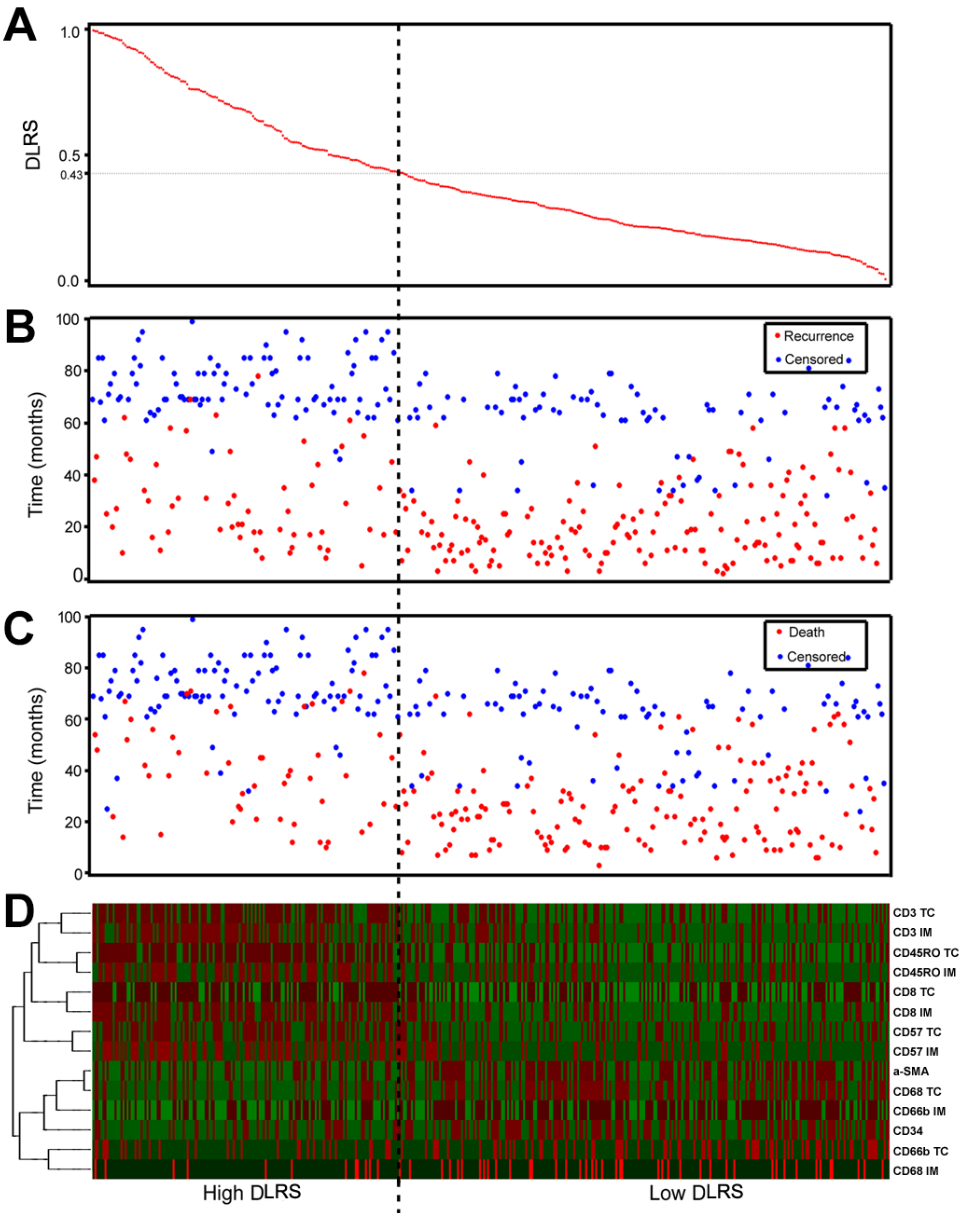

Figure S13. The deep learning score analysis of GC patients in the training cohort (n = 398). Related to Figure 3.

(A) DLRS score of GC patients. (B) Recurrence status of GC patients. (C) Survival status of GC patients. (D) Color-gram of the expression profiles of 14 tumor microenvironment features in GC patients. Rows represent 14 features and columns represent patients. Magenta dotted line represents the DLRS cutoff dividing the patients into high and low DLRS groups.

Internal validation cohort 1

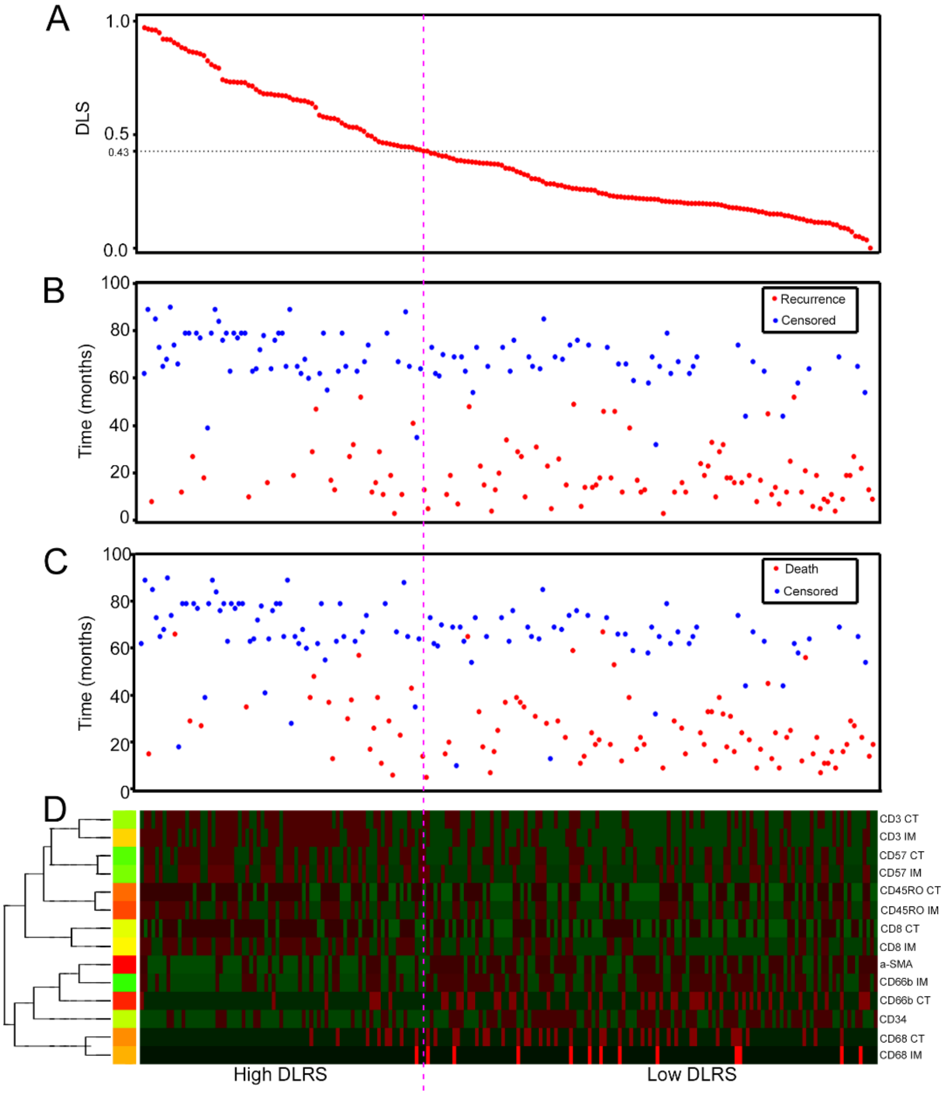

Figure S14. The deep learning score analysis of GC patients in the internal validation cohort 1 (n = 196). Related to Figure 3.

(A) DLRS score of GC patients. (B) Recurrence status of GC patients. (C) Survival status of GC patients. (D) Color-gram of the expression profiles of 14 tumor microenvironment features in GC patients. Rows represent 14 features and columns represent patients. Magenta dotted line represents the DLRS cutoff dividing the patients into high and low DLRS groups.

External validation cohort 1

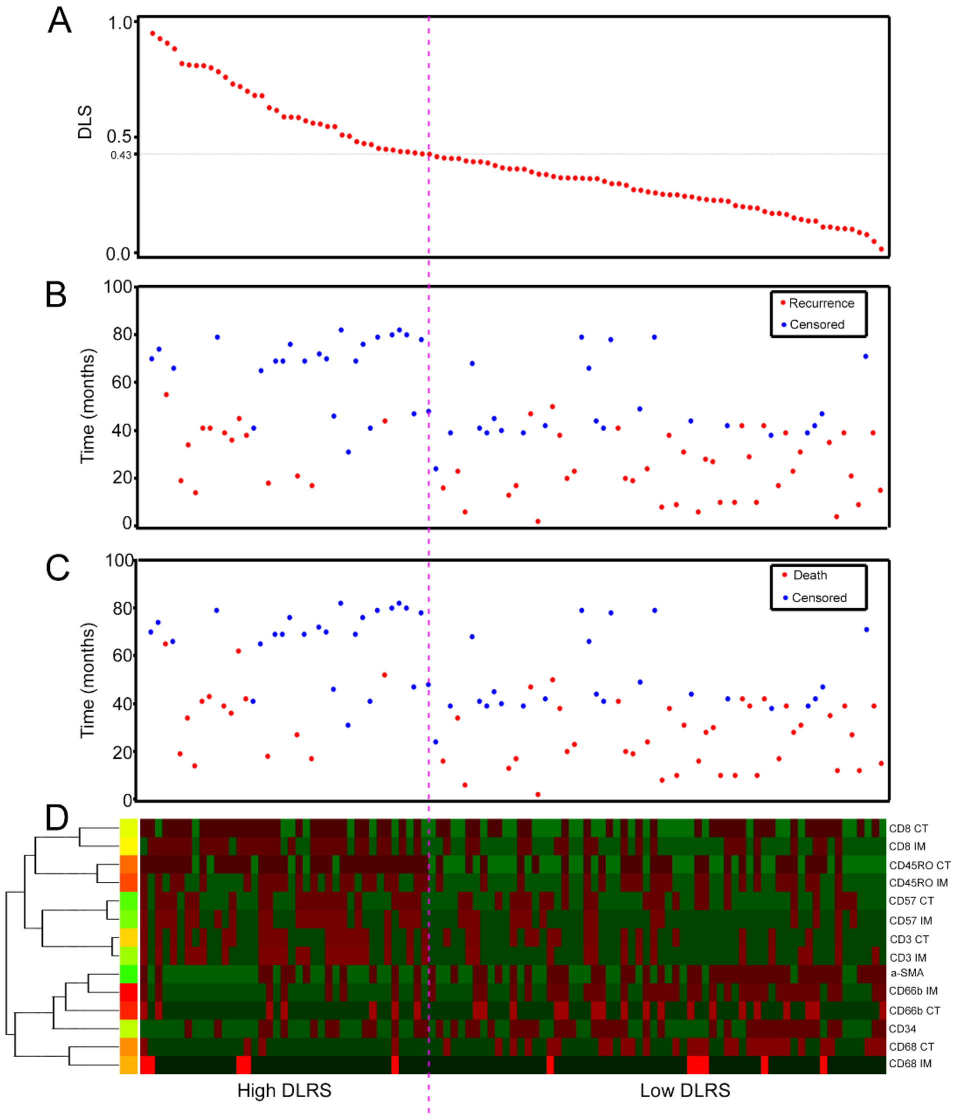

Figure S15. The deep learning score analysis of GC patients in the external validation cohort 1 (n = 101). Related to Figure 3.

(A) DLRS score of GC patients. (B) Recurrence status of GC patients. (C) Survival status of GC patients. (D) Color-gram of the expression profiles of 14 tumor microenvironment features in GC patients. Rows represent 14 features and columns represent patients. Magenta dotted line represents the DLRS cutoff dividing the patients into high and low DLRS groups.

A Disease-free survival

Cohort	Stage	DLRS	Number of patients	HR (95%CI)	P
Internal cohort	I	High VS. Low	76/133	0.325 (0.157 − 0.675)	0.003
Internal cohort	II	High VS. Low	100/214	0.352 (0.223 − 0.556)	<0.001
Internal cohort	III	High VS. Low	290/296	0.363 (0.294 − 0.448)	<0.001
Internal cohort	IV	High VS. Low	37/50	0.197 (0.113 − 0.343)	<0.001
External validation cohort	I	High VS. Low	67/129	0.131 (0.031 − 0.558)	0.006
External validation cohort	II	High VS. Low	103/204	0.376 (0.218 − 0.647)	<0.001
External validation cohort	III	High VS. Low	276/290	0.426 (0.342 − 0.529)	<0.001
External validation cohort	IV	High VS. Low	42/58	0.129 (0.074 − 0.227)	<0.001

B Overall survival

Cohort	Stage	DLRS	Number of patients	HR (95%CI)	P
Internal cohort	I	High VS. Low	76/133	0.285 (0.131 − 0.619)	0.002
Internal cohort	II	High VS. Low	100/214	0.280 (0.168 − 0.465)	<0.001
Internal cohort	III	High VS. Low	290/296	0.351 (0.282 − 0.436)	<0.001
Internal cohort	IV	High VS. Low	37/50	0.458 (0.285 − 0.738)	0.001
External validation cohort	I	High VS. Low	67/129	0.126 (0.030 − 0.538)	0.005
External validation cohort	II	High VS. Low	103/204	0.377 (0.218 − 0.652)	<0.001
External validation cohort	III	High VS. Low	276/290	0.423 (0.340 − 0.527)	<0.001
External validation cohort	IV	High VS. Low	42/58	0.186 (0.109 − 0.317)	<0.001

Figure S16. Forest plots summarize the analysis of disease-free survival and overall survival according to the DLRS signature in subgroups of GC patients in the internal cohort (combined training and internal validation cohorts) and external validation cohort. Related to Figure 3.
Internal cohort (SMU): Stage I (n = 209), Stage II (n = 314), Stage III (n = 586), Stage IV (n = 87).
External cohort (SYSUCC): Stage I (n = 196), Stage II (n = 307), Stage III (n = 566), Stage IV (n = 100).

Disease-free survival

Factors	DLRS	Number of patients	HR (95%CI)	P
T stage				
T1+T2	High VS. Low	223/446	0.254 (0.167 − 0.385)	<0.001
T3+T4	High VS. Low	768/928	0.477 (0.419 − 0.543)	<0.001
N stage				
N0	High VS. Low	291/506	0.507 (0.383 − 0.672)	<0.001
N1+N2+N3	High VS. Low	700/868	0.445 (0.388 − 0.510)	<0.001
Differentiation				
Well+Moderate	High VS. Low	254/398	0.504 (0.395 − 0.642)	<0.001
Poor+Undifferentiation	High VS. Low	737/976	0.506 (0.439 − 0.583)	<0.001
Size				
<4	High VS. Low	400/712	0.469 (0.383 − 0.574)	<0.001
>4	High VS. Low	591/662	0.494 (0.422 − 0.577)	<0.001
CEA				
Low	High VS. Low	836/1162	0.500 (0.435 − 0.573)	<0.001
High	High VS. Low	155/212	0.520 (0.399 − 0.677)	<0.001
CA199				
Low	High VS. Low	809/1137	0.460 (0.399 − 0.531)	<0.001
High	High VS. Low	182/237	0.633 (0.499 − 0.803)	<0.001
Lauren type				
Intestinal	High VS. Low	631/811	0.516 (0.444 − 0.601)	<0.001
Diffuse	High VS. Low	360/563	0.484 (0.393 − 0.595)	<0.001
Location				
Pylorus	High VS. Low	449/696	0.497 (0.411 − 0.601)	<0.001
Others	High VS. Low	542/678	0.498 (0.424 − 0.585)	<0.001
Age				
>60 years	High VS. Low	401/533	0.526 (0.436 − 0.634)	<0.001
<60 years	High VS. Low	491/710	0.462 (0.384 − 0.556)	<0.001
Gender				
Male	High VS. Low	714/901	0.538 (0.466 − 0.622)	<0.001
Female	High VS. Low	277/473	0.440 (0.349 − 0.555)	<0.001

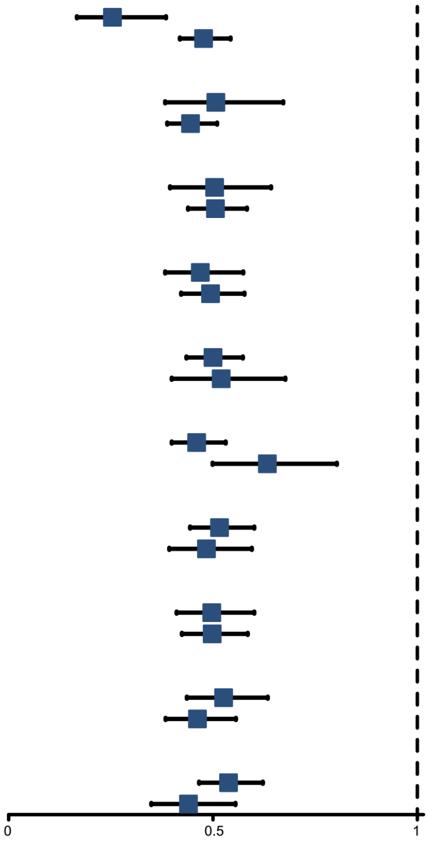

Figure S17. Forest plots summarize the analysis of disease-free survival for all the patients according to the predicted survival score stratified by clinicopathological risk factors. Related to Figure 3.

Overall survival

Factors	DLRS	Number of patients	HR (95%CI)	P
**T stage**				
T1+T2	High VS. Low	223/446	0.202 (0.128 – 0.320)	<0.001
T3+T4	High VS. Low	768/928	0.470 (0.412 – 0.537)	<0.001
**N stage**				
N0	High VS. Low	291/506	0.479 (0.358 – 0.639)	<0.001
N1+N2+N3	High VS. Low	700/868	0.427 (0.371 – 0.491)	<0.001
**Differentiation**				
Well+Moderate	High VS. Low	254/398	0.469 (0.364 – 0.603)	<0.001
Poor+Undifferentiation	High VS. Low	737/976	0.486 (0.420 – 0.561)	<0.001
**Size**				
<4	High VS. Low	400/712	0.441 (0.358 – 0.543)	<0.001
>4	High VS. Low	591/662	0.476 (0.406 – 0.558)	<0.001
**CEA**				
Low	High VS. Low	836/1162	0.500 (0.435 – 0.573)	<0.001
High	High VS. Low	155/212	0.520 (0.399 – 0.677)	<0.001
**CA199**				
Low	High VS. Low	809/1137	0.426 (0.367 – 0.493)	<0.001
High	High VS. Low	182/237	0.672 (0.528 – 0.854)	0.001
**Lauren type**				
Intestinal	High VS. Low	631/811	0.495 (0.424 – 0.578)	<0.001
Diffuse	High VS. Low	360/563	0.455 (0.368 – 0.564)	<0.001
**Location**				
Pylorus	High VS. Low	449/696	0.464 (0.382 – 0.564)	<0.001
Others	High VS. Low	542/678	0.482 (0.482 – 0.568)	<0.001
**Age**				
>60 years	High VS. Low	401/533	0.507 (0.418 – 0.614)	<0.001
<60 years	High VS. Low	491/710	0.440 (0.365 – 0.532)	<0.001
**Gender**				
Male	High VS. Low	714/901	0.519 (0.447 – 0.602)	<0.001
Female	High VS. Low	277/473	0.407 (0.320 – 0.517)	<0.001

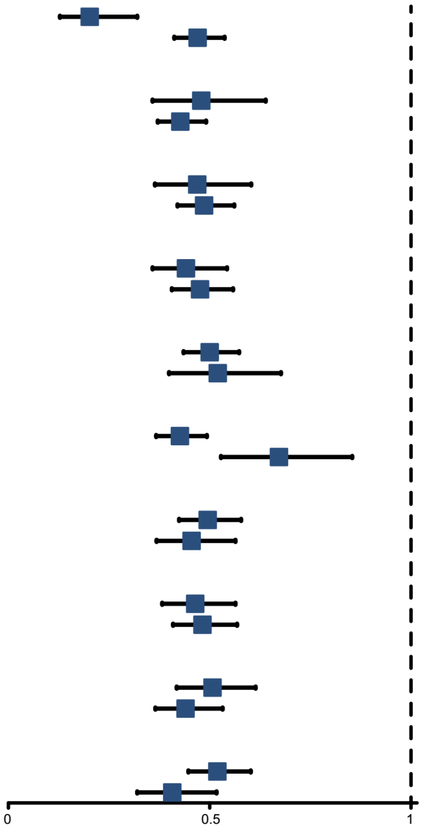

Figure S18. Forest plots summarize the analysis of overall survival for all the patients according to the predicted survival score stratified by clinicopathological risk factors. Related to Figure 3.

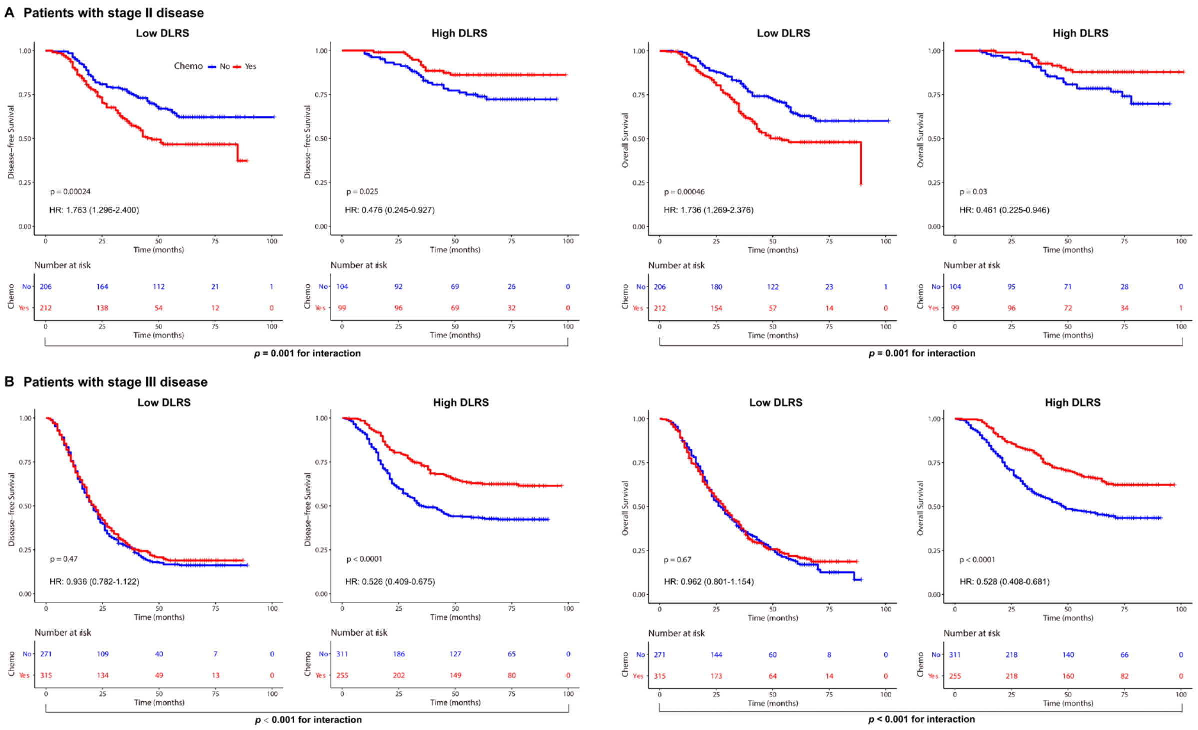

Figure S19. Relationship between the CT imaging-defined DLRS group and disease-free survival and overall survival in unmatched patients who were treated with or without adjuvant chemotherapy. Related to Figure 4 and 5.

A, stage II (n = 621). B, stage III (n = 1,152). Patients were stratified by the receipt of adjuvant chemotherapy. Statistical interaction tests were performed for the following: (A) predicted DLRS classes Low vs. High and adjuvant chemotherapy: $P_{interaction}$ = 0.001 and 0.001 for disease-free survival and overall survival in stage II patients; (2) predicted DLRS classes Low vs High and adjuvant chemotherapy: $P_{interaction}$ <0.001 and <0.001 for disease-free survival and overall survival in stage III patients.

A

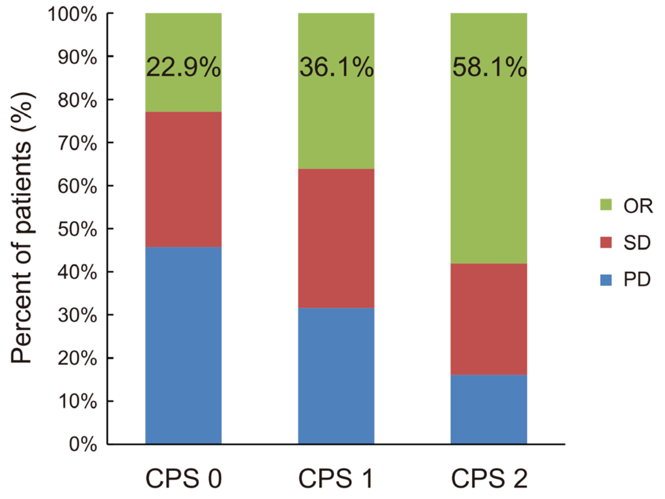

B

Multivariate logistic regression analysis for objective response in patients with different CPS.

Subgroup	Number of patients	OR (95% CI)	P
**CPS High**			
**DLRS High VS. Low**	62/31	2.703 (1.114 − 6.559)	0.028
**CPS Moderate**			
**DLRS High VS. Low**	84/74	17.515 (6.824 − 44.955)	<0.001
**CPS Low**			
**DLRS High VS. Low**	41/29	99999 (0.000 − 999999)	0.998

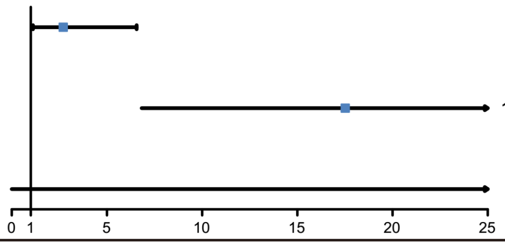

Figure S20. Proportion of response to anti-PD-1 immunotherapy response in different CPS (A), and multivariate logistic regression analysis for objective response in patients with different CPS (B). Related to Figure 6.

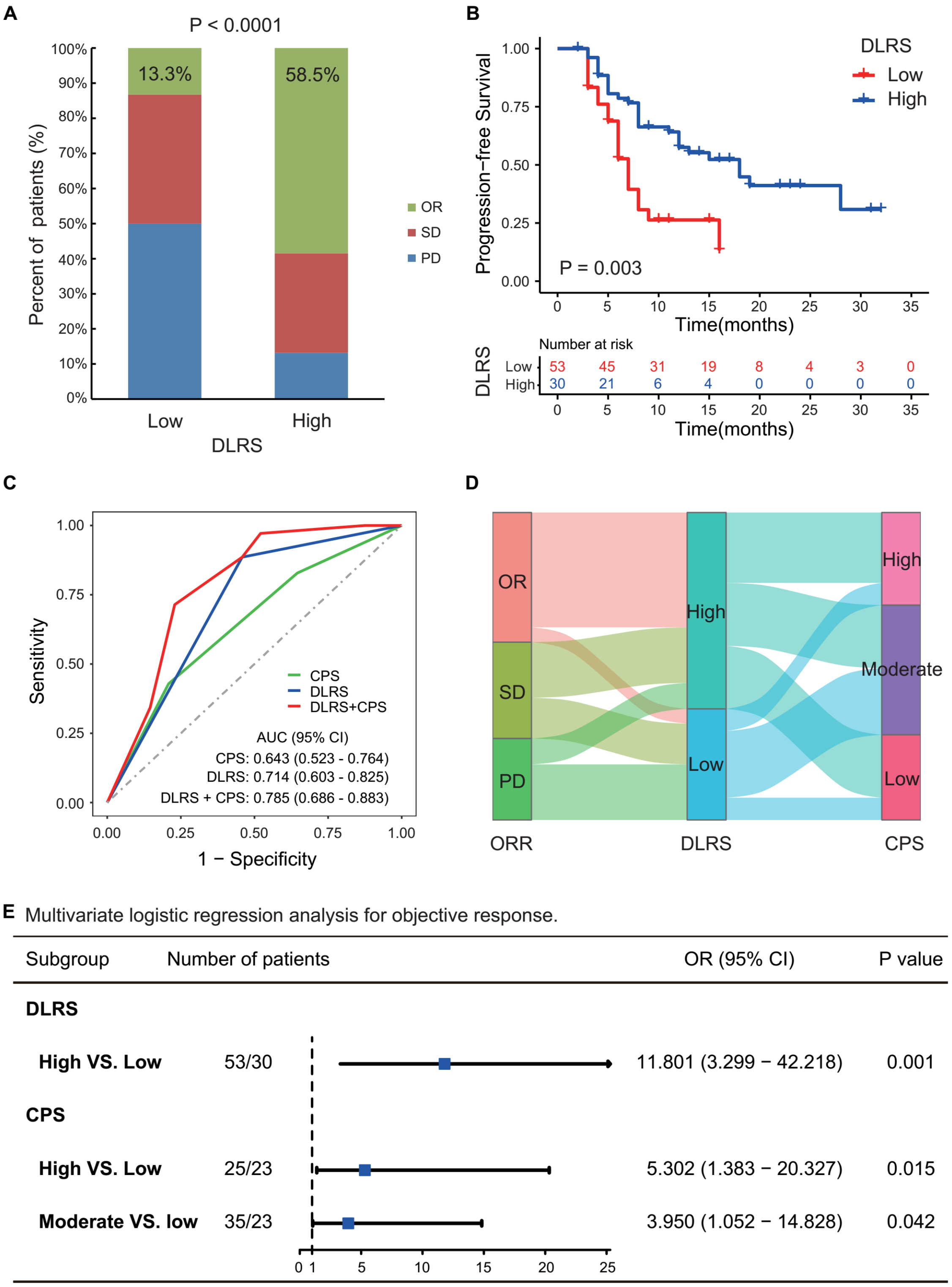

Figure S21. Relationship between the imaging based TME classifier and clinical response and outcomes in patients treated with anti-PD-1 immunotherapy. Related to Figure 6.

(A): Response rates in patients of the DLRS high vs low groups; (B): Progression-free survival in patients of the DLRS high vs low groups; (C), Receiver operator characteristic (ROC) curves of the predicted TME classes, CPS and composite models combining TME classes and CPS for predicting

immunotherapy response (n=83); AUC: DLRS vs. CPS, *P*=0.362; DLRS+CPS vs. CPS, *P*=0.01; DLRS+CPS vs. DLRS, *P*=0.033; (D), Alluvial diagram of the correspondence among patients classified according to the immunotherapy response, DLRS, and CPS in the merged immunotherapy cohorts (n = 83); (E), Forest plot for the multivariate logistic regression analysis for objective response; AUC: area under the receiver operator characteristic curve. CPS: combined positive score of PDL1 expression. OR: objective response (complete and partial response); SD: stable disease; PD: progressive disease.

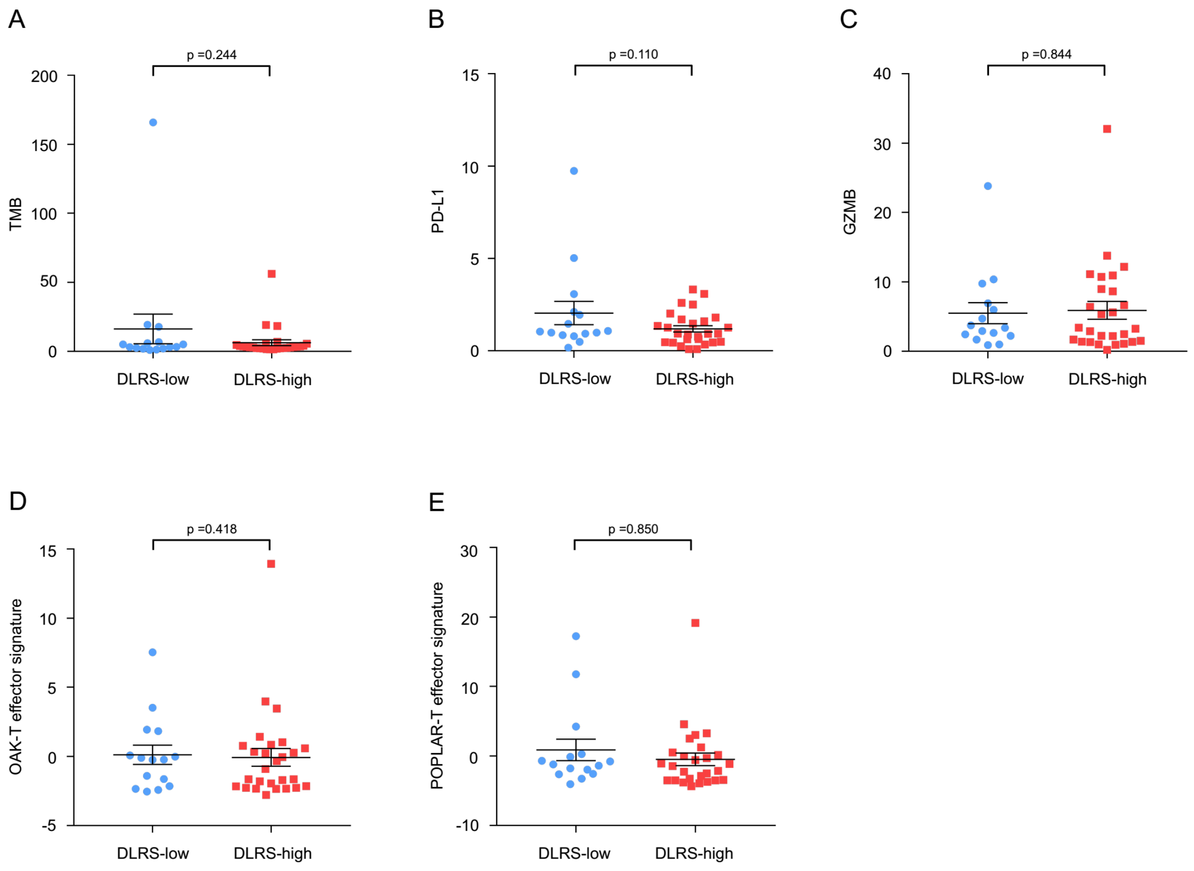

Figure S22. Relationship between the proposed imaging signature DLRS and established genomic and transcriptomic biomarkers, including TMB (A), PD-L1 (B), GZMB (C), OAK-T effector signature (D), and POPLAR-T effector signature (E). Related to Figure 7.
